# Uncovering synthetic lethal interactions for therapeutic targets and predictive markers in lung adenocarcinoma

**DOI:** 10.18632/oncotarget.12046

**Published:** 2016-09-15

**Authors:** Jan-Gowth Chang, Chia-Cheng Chen, Yi-Ying Wu, Ting-Fang Che, Yi-Syuan Huang, Kun-Tu Yeh, Grace S. Shieh, Pan-Chyr Yang

**Affiliations:** ^1^ Department of Laboratory Medicine and Epigenome Research Center, China Medical University Hospital, China Medical University, Taichung, Taiwan; ^2^ Institute of Statistical Science, Academia Sinica, Taipei, Taiwan; ^3^ Graduate Institute of Clinical Medicine, College of Medicine, National Cheng Kung University, Tainan, Taiwan; ^4^ Institute of Biomedical Sciences, Academia Sinica, Taipei, Taiwan; ^5^ Department of Pathology, Changhua Christian Hospital, Changhua, Taiwan; ^6^ Department of Pathology, School of Medicine, Chung Shan Medical University, Taichung, Taiwan; ^7^ Bioinformatics Program, Taiwan International Graduate Program, Academia Sinica, Taipei, Taiwan; ^8^ Genome and Systems Biology Degree Program, Academia Sinica and National Taiwan University, Taipei, Taiwan; ^9^ Center of Genomic Medicine, National Taiwan University, Taipei, Taiwan; ^10^ Department of Internal Medicine, National Taiwan University Hospital, Taipei, Taiwan

**Keywords:** lung adenocarcinoma, TP53, prognosis marker, synthetic lethal, gene expression data

## Abstract

Two genes are called synthetic lethal (SL) if their simultaneous mutation leads to cell death, but mutation of either individual does not. Targeting SL partners of mutated cancer genes can selectively kill cancer cells, but leave normal cells intact. We present an integrated approach to uncover SL gene pairs as novel therapeutic targets of lung adenocarcinoma (LADC). Of 24 predicted SL pairs, PARP1-TP53 was validated by RNAi knockdown to have synergistic toxicity in H1975 and invasive CL1-5 LADC cells; additionally FEN1-RAD54B, BRCA1-TP53, BRCA2-TP53 and RB1-TP53 were consistent with the literature. While metastasis remains a bottleneck in cancer treatment and inhibitors of PARP1 have been developed, this result may have therapeutic potential for LADC, in which TP53 is commonly mutated. We also demonstrated that silencing PARP1 enhanced the cell death induced by the platinum-based chemotherapy drug carboplatin in lung cancer cells (CL1-5 and H1975). IHC of RAD54B↑, BRCA1↓-RAD54B↑, FEN1(N)↑-RAD54B↑ and PARP1↑-RAD54B↑ were shown to be prognostic markers for 131 Asian LADC patients, and all markers except BRCA1↓-RAD54B↑ were further confirmed by three independent gene expression data sets (a total of 426 patients) including The Cancer Genome Atlas (TCGA) cohort of LADC. Importantly, we identified POLB-TP53 and POLB as predictive markers for the TCGA cohort (230 subjects), independent of age and stage. Thus, POLB and POLB-TP53 may be used to stratify future non-Asian LADC patients for therapeutic strategies.

## INTRODUCTION

Lung cancer is the most common cancer worldwide in terms of both incidence and mortality [1]. Nearly 40% of lung cancers are adenocarcinoma. Most cases of adenocarcinoma are associated with smoking, but among never-smokers, adenocarcinoma is the most common subtype of lung cancer. Multiple genes are mutated during tumorigenesis, with those contributing to tumor formation and growth are called cancer genes [2]. Cancer genes can be categorized into oncogenes, tumor suppressor genes and stability genes. Mutant oncogenes and tumor suppressor genes drive cancer cell proliferation while stability genes are involved in maintenance of genome integrity. Although to date some therapeutics directed against oncogenes have led to increases in patient survival, many fail due to intrinsic or adaptive resistance of the cancer cell population to the therapeutics. For example, in lung adenocarcinoma, efficacy of EGFR-inhibition is reduced by downstream KRAS-activating mutation [3].

Two genes are called synthetic lethal (a type of genetic interaction described in [4]) when a simultaneous mutation of both genes leads to cell death, but a single mutation of either does not. Genome-scale mappings of SL genes in *S. cerevisiae* were obtained through high-throughput synthetic genetic array analyses [5–7]. Wong and colleagues [8] successfully predicted synthetic sick or lethal (SSL) interactions in *S. cerevisiae* by integrating multiple types of data, e.g., gene expression, protein-protein interaction and properties of network topology of a gene triple. In *C. elegans*, Zhong and Steinberg [9] computationally integrated interactome, gene expression and phenotype data to predict genome-wide genetic interactions; they further experimentally verified the predictions for two human disease-associated genes.

The concept of synthetic lethality can be applied to exploit cancer-cell specific mutations for therapeutics [10] as follows. Targeting synthetic lethal (SL) partners of mutated cancer genes will selectively kill cancer cells but spare normal cells. Therefore, the synthetic lethality strategy offers an elegant alternative to killing cancer cells with non-druggable mutant tumor suppressor genes and stability genes, for example, *TP53* and *BRCA1*, by targeting their SL partners. The clinical relevance of synthetic lethality has been rapidly recognized. For example, pioneering studies of SL partners in *BRCA1* and *BRCA2-*deficient cancer cells identified PARP1 as a promising drug target [11, 12]. Multiple phase II and III clinical trials of PARP inhibitors have been conducted for breast and ovarian cancer patients with *BRCA1* or *BRCA2* mutation [13, 14]. Over the last few years, genes having SL interactions (SLs) in cancers have been actively studied by individual RNAi experiments or large scale RNAi screenings that uncover multiple SL gene pairs [15–17], which in general were centered on one gene such as *KRAS*. Astsaturov and colleagues identified SLs in humans by combining both computational and experimental approaches [18]. They first combined pathway maps, protein-protein interactions, gene expression data and human orthologs of *Drosophila Egfr* genetic interaction partners, to predict 2,689 SL candidates of *EGFR*. They then selected 683 candidates for RNAi screening, by their appearance in at least two of these information sources or by prior biological knowledge, which resulted in 61 SL genes of *EGFR*. Recently, Jerby-Amon and colleagues [19] developed an extensive computational pipeline to analyze a large volume of genomics data, by detecting events of co-inactive genes that occur less often than expected and analyzing somatic number alterations and DNA mutation, followed by RNAi knockdown experiments to identify SL gene pairs and drug resistant pairs in cell lines. Importantly, various inhibitors of PARP1 for patients with *BRCA1/2* mutation have entered phase III trials in breast, ovarian, pancreatic cancer and glioblastoma [20]).

**Figure 1:**
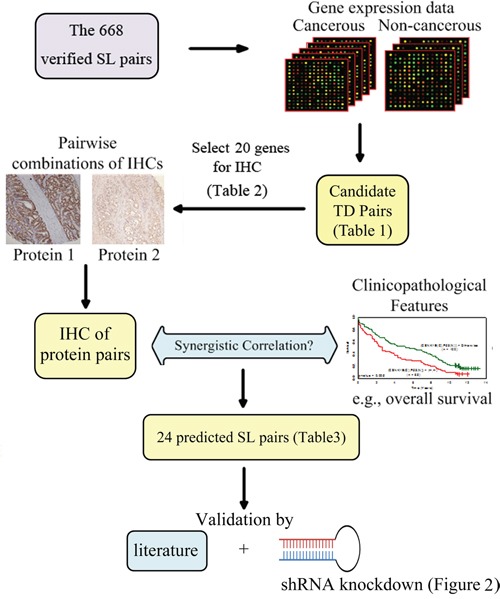
A graphical schematics of our approach Microarray gene expression of 83 paired cancerous and non-cancerous tissues was used to sift tumor dependent gene pairs of lung adenocarcinoma from 668 collected synthetic lethal (SL) pairs, which resulted in 20 genes for immunohistochemistry (IHC). Combining the 23 IHC into pairs and correlating them to the each of five clinical factors yielded 24 predicted SL pairs. Finally, we validated the predicted SL pairs by the literature and shRNA knockdown.

Although large-scale RNAi screening to identify SL pairs has been available, the false positive problem remains to be resolved. To augment these RNAi-based experimental methods, we have developed an integrated approach to uncover SL gene pairs for lung adenocarcinoma (LADC). Our approach incorporates SL pairs in the literature, gene expression data, protein expression (immunohistochemistry (IHC) of LADC tissues) and phenotypic (clinicopathological factors) data, to predict SL gene pairs, then two prioritized pairs are verified by shRNA inhibition in lung cancer cells. Prognostic and predictive markers are also explored, and validated by multiple sets of external data. A graphical representation of our approach is presented in Figure 1.

## RESULTS

### Initial panel of candidate tumor-dependent gene pairs for lung adenocarcinoma

We first collected 668 SL pairs, most of which were validated by genome-wide RNAi screenings in various cancers [15, 16], while the remaining SLs were verified via individual RNAi knockdown experiments using cancer cell lines [11, 12]. By screening across these SL pairs using microarray gene expression data of various cancerous and non-cancerous tissues (a pilot study), we found that some gene pairs, e.g., *FEN1*-*RAD54B*, were simultaneously differentially expressed in high fractions of several cancerous tissues including LADC. This was consistent with the finding that co-expression of gene pairs was a relevant feature for predicting genetic interactions (including SL interactions) genome-wide in *S. cerevisiae* and *C. elegans* [6, 8, 9].

In general, tumor cells depend on the overexpression of oncogenes and/or under-expression of tumor suppressor genes. Validated SL pairs comprised oncogenes, tumor-suppressor genes and stability genes [2]. We hypothesized that tumor cells may depend on differential expression of some of these gene pairs and their protein products for survival and/or proliferation. Further, the more closely functionally related an SL pair is, the more frequently the genes may be simultaneously differentially expressed in cancer tissues. Thus, the collected SL gene pairs were sifted by 83 paired Asian LADC tumor and non-cancerous tissues. The fractions of the co-expression (up, up), (up, down), (down, up) and (down, down) patterns, where up and down denoting up- and down-regulation with the cutoff 1.5-fold, were computed (see Materials and Methods for details). To include as many potential TD pairs as possible, any gene pair with any pattern exceeding one percent was included. Because RNAi knockdown is easier than overexpression of a particular gene, we sorted the gene pairs by the fractions of the (up, up), (up, down), (down, up), and then (down, down) patterns as shown in Table 1, which includes genes highly mutated in Asian LADC such as *EGFR* and *TP53* [21] (see Materials and Methods).

**Table 1:** The initial panel of candidate tumor dependent pairs for lung adenocarcinoma (LADC), with fractions of the four differentially expressed patterns by the threshold 1.5-fold, screened from 668 collected synthetic lethal (SL) gene pairs

Tumor dependent pairs		Fractions of SL gene pairs computed from 83 Asian LADC versus non-cancerous tissues that were expressed 1.5-fold or higher					
Gene 1	Gene 2	(up, up) pattern	(up, down) pattern	(down, up) pattern	(down, down) pattern	Permutation *p*-value	*q*-value
*FEN1*	*RAD54B*	0.31^a^	0.01	0.00	0.01	0.0001	0.0043
*BRCA1*	*PARP1*	0.18	0.00	0.00	0.00	0.0001	0.0043
*MSH2*	*POLB*	0.14	0.01	0.02	0.00	0.0006	0.0156
*BCR*	*WNT5A*	0.11	0.01	0.01	0.00	0.0023	0.0473
*BRCA2*	*PARP1*	0.11	0.00	0.00	0.00	0.0022	0.0473
*TP53*	*SGK2*	0.06	0.00	0.00	0.00	0.0273	0.2367
*MYC*	*AURKB*	0.05	0.00	0.23	0.00	0.0001	0.0043
*ABL1*	*WNT5A*	0.05	0.00	0.06	0.07	0.3871	0.5180
*TP53*	*PAK3*	0.02	0.06	0.02	0.01	0.2279	0.4757
*EGFR*	*SL_EGFR_*^b^	0.01~0.18	0.00	0.00	0.00	0.0001~0.4940	0.0043~0.5180
*EGFR*	*SL_EGFR_*^c^	0.00	0.01~0.20	0.00	0.00	0.0001~0.5084	0.0043~0.5180
*KRAS*	*SL_KRAS_*^d^	0.01~0.07	0.00	0.00	0.00	0.0143~0.7506	0.1642~0.7506
*KRAS*	*SL_KRAS_*^e^	0.00	0.01~0.07	0.00	0.00	0.0298~0.7410	0.2367~0.7448

a The four fractions were computed from gene pairs that were 1.5-fold differentially expressed, thus they might not sum up to 100%.

b 22 verified EGFR SL pairs were identified in the (up, up) pattern.

c 30 verified EGFR SL pairs were identified in the (up, down) pattern.

d 168 verified KRAS SL pairs were identified in the (up, up) pattern.

e 121 verified KRAS SL pairs were identified in the (up, down) pattern.

The *p*-value for the highest fraction of the four patterns was computed by permutation test with 10,000 repeats, and the false discovery rate was estimated by *q*-value.

Although the overexpression of tumor-suppressor and stability gene pairs, for example, DNA repair and checkpoint classes such as FEN1 in Table 1, seem surprising at first glance, it is consistent with the drastic increase in genomic instability and DNA replication caused by mutant oncogenes such as *KRAS* and *MYC*.

### Correlating IHC staining of TD pairs with clinicopathological factors identifies SL pairs

Next, from Table 1 we selected 20 genes, which were from the top-11 gene pairs excluding MET and PAK3, but additionally including CSNK1E and CDH1 (SL to TP53 and EGFR, respectively). IHC of the 20 proteins were stained; most were stained at one cellular location, except BRCA2, FEN1 and MSH2 which were stained at two cellular locations (Table 2; some representative IHC figures are shown in Supplementary Figure S1), using LADC tissues dissected from 131 patients from Changhua Christian Hospital, Changhua City, Taiwan (see Materials and Methods). IHC of these proteins confirmed the trends of their mRNA expression.

**Table 2:** The list of immunohistochemistry (IHC) proteins stained from cancerous tissues which were dissected from 131 local lung adenocarcinoma patients, their criteria for abnormal IHC and the factions of these patients with abnormal IHC

No.	Protein name	Criterion of abnormality	% of patients with abnormal IHC
1	ABL1(C)^a^	≧ 2+	24
2	AURKB(C)	≧ 1+	91
3	BCR(C)	≧ 2+	27
4	BRCA1(C)	**<** 1+	77
5	BRCA2(C)	**<** 2+	67
6	BRCA2(N)^a^	**<** 2+	19
7	CDH1(M)^a^	≧ 70%	62
8	CSNK1E(C)	<2+	94
9	CTNNB1(N)	≧ 1+	6
10	EGFR(C)	≧ 1+	19
11	FEN1(C)	≧ 2+	58
12	FEN1(N)	≧ 2+	50
13	MSH2(C)	< 1+	31
14	MSH2(N)	<1+	20
15	MYC(N)	≧ 2+	42
16	PARP1(N)	≧ 1+	87
17	POLB(N)	≧ 1+	91
18	RAD54B(N)	≧ 1+	33
19	RB1(N)	< 1+	55
20	SGK2(C)	< 2+	96
21	SKP2(C)	≧ 2+	10
22	TP53(N)	≧ 1+	51
23	WNT5A(C)	≧ 1+	60

a The notation (C), (N) and (M) represent cytoplasm, nucleus and membrane, respectively.

To extend the analysis beyond the known SL pairs (Supplementary Table S1), we combined the 23 individual protein IHCs in Table 2 into all the possible distinct pairs (250 in total), excluding the pairs of the same protein stained in different cellular locations. This procedure allows novel SL pairs to be unraveled. The cancer phenotypes observed (for instance, tumor grade) suggested that the tumor cells may depend on each protein pair for viability and, therefore, simultaneously mutating each TD pair may kill tumor cells. Further, protein pairs relevant to tumor cell viability and malignancy may be indicated by their paired correlation with clinical features of LADC patients. To identify SL pairs, we then tested each protein pair for two synergistic effects with five clinical features, overall survival, tumor grade, lymph node metastasis, metastasis and stage (Supplementary Dataset S1). For simplicity, henceforth, we have omitted the location attached to the protein name; for example, CSNK1E(C) is written as CSNK1E, unless ambiguity arises.

### Synergistic correlation

A pair of proteins is said to have a synergistic correlation if their paired abnormal IHC is significantly correlated (*P* < 0.05; Fisher's exact test) with a clinicopathological feature (for example, tumor grade), but the abnormal IHC of each single protein is not. This synergistic effect can be utilized to uncover tumor dependent gene pairs, because an individual gene's mutation is not significantly correlated to a phenotype (clinical feature); however, simultaneous mutation of a gene pair is.

Any protein pair passing this screening for synergy was predicted to be SL, because this synergistic effect implies that abnormal IHC of a protein pair is associated with a cancer phenotype (for example, overall survival), but the abnormal IHC of each individual protein is not. In other words, LADC cells seem to depend on each of these protein pairs, but not on each individual protein, for maintenance of viability. Thus, simultaneous mutation of each TD pair may eliminate the tumor cells, which is synthetic lethality [22]. Although coexpression and phenotype have been shown to be useful in predicting SL interactions in model organisms [6, 8, 9], this synergistic effect is novel in (1) prioritizing the gene pairs co-expressed in cancer cells and (2) treating clinical features of cancer patients as phenotypes.

### Putative synergistic correlation

Next, we relaxed this synergistic correlation to a putative synergistic correlation, which allowed any individual IHC of an IHC pair to be significantly correlated with a clinical feature, but both individual IHCs were less correlated (in terms of *p-*value being larger) with a clinical feature than their IHC pair.

Applying both synergistic effects to the 250 IHC pairs resulted in 24 predicted SL pairs (Table 3). Those marked in light yellow were predicted by the synergistic correlation, while the remaining ones were predicted from the putative synergistic correlation. In each synergistic effect, Fisher's exact test (see Materials and Methods) was applied to test the significance of the correlation (*P* < 0.05), and the false discovery rate of a test was estimated by the q-value (qvalue in R), which measured the proportion of false positives incurred when the test was claimed significant [23]. The complete results are shown in Supplementary Table S2. Although FDR values in Table 3 may seem large at a glance, but their cutoff should refer to similar studies, e.g. 0.467 of CTNNB1-TP53 in [22], which was verified to be SL.

**Table 3:** The predicted synthetic lethal pairs which had (putative) synergistic correlation when tested against five clinical features, where the synergistic correlation was conducted by multiple Fisher's tests; the false positive rate (FDR) was estimated by q-value

Protein1	Protein2	*p*-value			FDR	Log-rank test^a^ (*p*-value)
		Protein1	Protein2	Protein Pairs		
**A. Overall Survival**						
FEN1(N)	RAD54B(N)	0.357	0.002	0.001	0.126	0.000
BRCA1(C)	RAD54B(N)	0.442	0.002	0.001	0.126	0.000
PARP1(N)	RAD54B(N)	0.252	0.002	0.002	0.162	0.000
BRCA1(C)	FEN1(N)	0.442	0.357	0.016	0.356	0.056
**B. Metastasis**						
CSNK1E(C)	TP53(N)	0.682	0.012	0.003	0.369	0.420
BRCA1(C)	TP53(N)	0.281	0.012	0.005	0.369	0.129
POLB(N)	TP53(N)	0.485	0.012	0.007	0.369	0.108
PARP1(N)	TP53(N)	0.299	0.012	0.008	0.369	0.414
RB1(N)	TP53(N)	0.114	0.012	0.008	0.369	0.092
BRCA1(C)	RB1(N)	0.281	0.114	0.037	0.897	0.756
**C. Lymph node Metastasis**						
CDH1(M)	MSH2(C)	0.472	0.084	0.007	0.973	0.280
BRCA2(C)	MSH2(C)	0.245	0.084	0.016	0.973	0.520
FEN1(N)	MSH2(C)	0.212	0.084	0.022	0.973	0.502
BRCA1(C)	EGFR(C)	0.355	0.103	0.033	0.973	0.857
BRCA2(N)	TP53(N)	0.190	0.351	0.045	0.973	0.483
MSH2(C)	RB1(N)	0.084	0.220	0.045	0.973	0.306
**D. Grade**						
ABL1(C)	CTNNB1(N)	0.781	0.169	0.021	0.931	0.842
ARK2(C)	BRCA2(N)	0.380	0.108	0.031	0.931	0.720
BRCA2(N)	POLB(N)	0.108	0.384	0.039	0.931	0.985
MSH2(C)	MYC(N)	0.058	0.448	0.047	0.931	0.347
BRCA1(C)	BRCA2(N)	0.407	0.108	0.049	0.931	0.338
**E. Stage**						
CDH1(M)	RB1(N)	0.346	0.107	0.012	0.974	0.632
EGFR(C)	RB1(N)	0.268	0.107	0.031	0.974	0.882
FEN1(N)	MSH2(C)	0.538	0.395	0.033	0.974	0.502
BRCA1(C)	TP53(N)	0.217	0.144	0.038	0.974	0.129
PARP1(N)	RB1(N)	0.381	0.107	0.048	0.974	0.234

a For each of those pairs with *P* < 0.05, the survival curve of LADC patients with both proteins abnormally expressed is separated from that of the remaining group.

Those marked in yellow were predicted by the original synergistic correlation, while the remaining ones by the putative synergistic effect.

Checking the predicted SL pairs (Table 3) against the literature (searched up to December 2014), we found that *FEN1-RAD54B*, and *BRCA1-TP53* were validated SL pairs [24, 25] in colorectal cancer and cervical cancer cells, respectively. Knockdown of *BRCA1*, *BRCA2* or *BRCA1/2* rendered TP53*-*deficient cervical cancer (HeLa) cells 4- to 7-fold more sensitive to cisplatin treatment than matched wild-type *TP53* cells [25]. These validated pairs demonstrate that our method is able to uncover SL gene pairs without being limited by pathway information or prior knowledge of whether a gene is involved in cancer. Mutation in genes involved in DNA repair (e.g., *BRCA1*) or in DNA damage stress response pathways such as *TP53*, can lead to increased DNA damage and genome instability. Therefore, double mutations in *TP53*-*BRCA1* or *TP53-BRCA2* may kill cells by “stress overload” [26].

Of these predicted SL pairs in Table 3, two verified in the literature were predicted by the synergistic effect and four pairs were predicted by the putative synergistic effect. In particular, checking the putative synergistic effect of the IHC pairs with metastasis led to three verified *TP53* SL pairs. Note that *TP53* has a very large sequence mutation score in LADC (Figure 1 and Supplementary Table S6a in [21]) and it is mutant in half of all cancers. Predicted pairs ranking higher than the known SL pairs in Table 3 suggested six candidate SL pairs. Further, we sifted these candidate pairs using the “2hop SL-SL” concept (Table 1 of [8]), namely if *PARP1* was SL to *BRCA1* and *BRCA1* was SL to *TP53*, then *PARP1* was likely SL to *TP53*; similarly *CSNK1E-RB1* might be SL.

Our rules for prioritizing predicted SL pairs for validation were, (1) incorporating prior knowledge, for example, known SL pairs or/and pathway information; and (2) using biological importance, for example, predicted SL pairs consisting of frequently mutated cancer genes in the cancer of interest.

### *PARP1-TP53* is validated to synergistically kill lung cancer cells by RNAi knockdown

**Figure 2:**
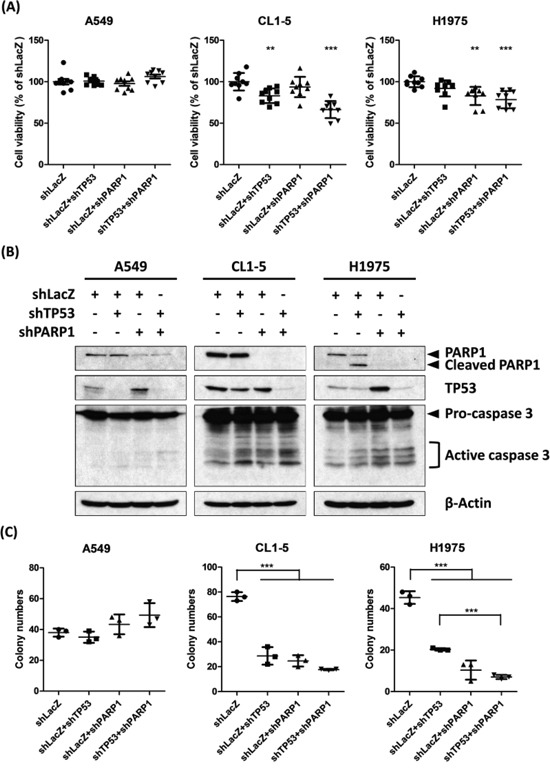
RNAi knockdown of TP53 and PARP1 shows synergistic killing in both CL1-5 and H1975 cells **A.** 2000 of A549, CL1-5 and H1975 cells were infected with lentivirus containing the indicated shRNAs for 72 hours. Cell viability was measured by MTT assay and compared to the control shLacZ group, the number of replicates (n) was nine per group. Values are reported as the mean ± standard error, where ** and *** denote p-value < 0.01 and < 0.001 from two-sample t-test, respectively. **B.** RNAi knockdown of *TP53* and *PARP1* by the indicated shRNA lentivirus in A549, CL1-5 and H1975 cells for 72 hours. Cell lysates were analyzed by western blotting using the indicated antibodies. Cleaved PARP and active caspase 3 were the apoptotic indicators and β-actin was the internal control. **C.** The long-term effects of RNAi knockdown of *TP53* and *PARP1* were examined by colony formation assay. 250 of A549, CL1-5 and H1975 cells infected with lentivirus containing the indicated shRNAs were cultured for 10 days. Colonies were stained by crystal violet and counted. n = 3 per group, and *** denotes p-value < 0.001 from two-sample t-test.

We selected three lung cancer cell lines, A549, CL1-5 and H1975, as *in vitro* models to validate that TP53-PARP1 is SL. A549 cells with the wild types of *EGFR* and *TP53* carry the *KRAS^G12S^* mutant. CL1-5 cells are highly invasive and harbor the *TP53^R248W^* mutant and the wild types of *EGFR* and *KRAS*, while H1975 cells harbor the wild-type *KRAS*, the *TP53^R273H^* mutant and the *EGFR^L858R T790M^* somatic mutation, which is commonly mutated in EGFR-tyrosine kinase inhibitor-resistant Asian LADC. We transiently co-infected A549, CL1-5 and H1975 cells with lentiviral vectors expressing shRNAs targeting *TP53*, *PARP1*, *PARP1-TP53* and control, respectively (Figure 2). Knockdown effect of shRNA was validated by PCR (Supplementary Figure S2) and the western blot analysis. Cell viability was examined by the MTT assay and the colony formation assay for detecting the short-term and the long-term cell killing effects, respectively. The results of the MTT assay (Figure 2A) showed that knockdown of *TP53* or *PARP1* did not affect the cell viability in A549 cells. However, *TP53* knockdown or knockdown of both *TP53* and *PARP1 in CL1-5 cells reduced the cell viability while PAPP1* knockdown or knockdown of both *TP53* and *PARP1 in H1975 cells also decreased it. The p*-values of the viability of cells with knockdown of *TP53* and *PARP1* individually compared to the control are 0.837 and 0.600 in A549 cells, 0.002 and 0.256 in CL1-5, and 0.057 and 0.001 in H1975 cells, respectively. Moreover, knockdown of both genes markedly reduced the cell viability in CL1-5 (*P* < 0.0001) and H1975 cells (*P* < 0.0001), but not in A549 cells (*P* = 0.154). Further, we found that active caspase 3 was increased in CL1-5 and H1975 cells with knockdown of *TP53*, *PARP1* and both (Figure 2B), indicating that knockdown of *TP53* and *PARP1* induced apoptosis in CL1-5 and H1975 cells.

To examine the long-term cell killing effects of gene knockdown, the colony formation assay was performed. Similar to the MTT results, knockdown of *TP53*, *PARP1* or both remarkably and significantly reduced the colony number in CL1-5 and H1975 cells, but not in A549 (Figure 2C). Thus *TP53* and *PARP1* have a synergistic killing effect in CL1-5 and H1975 cells, but not in A549 cells, which may be due to a different mutation background in A549 cells from CL1-5 and H1975 cells. Similarly, we used these three cell lines to verify whether *FEN1-TP53* is SL, however, no SL effect could be validated in these cell lines (data not shown). Nevertheless, our approach successfully uncovered the synthetic lethality of *PARP1-TP53 in highly metastatic lung cancer cells.*

Moreover, we studied the cytotoxic effect of the combination of PARP1 silencing and some chemotherapy drugs for lung cancer, such as carboplatin and pemetrexed, by the MTT assay. We found that PARP1 knockdown enhanced carboplatin and pemetrexed-induced cell death in CL1-5 and H1975 cells (*P* < 0.05; two sample t-test), except one case had marginal significance (CL1-5 cells treated with 100 nM Pemetrexed having *P* = 0.052), but not A549 cells. Compared to pemetrexed, carboplatin may be better for NSCLC treatment (Supplementary Figure S3).

### RAD54B, BRCA1-RAD54B, FEN1(N)-RAD54B, and PARP1-RAD54B are correlated with poor overall survival

**Figure 3:**
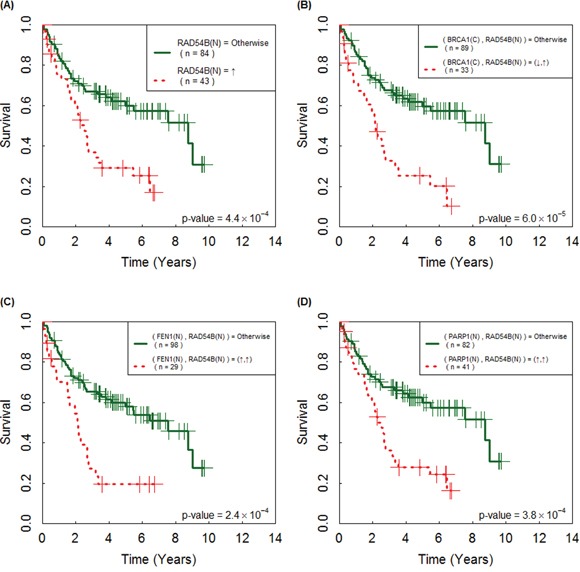
Immunohistochemistry (IHC) of the following individual and paired proteins are correlated with poor prognosis of 131 Asian lung adenocarcinoma patients Their Kaplan-Meier survival curves were significantly separated by **A.** RAD54B, **B.** BRCA1(C)-RAD54B, **C.** FEN1(N)-RAD54B and **D.** PARP1-RAD54B. The curves for the patients with paired abnormal IHC (according to Table 2) are plotted in dashed line and the curves of the remaining patients are in solid line; the symbols ↑ and ↓ denote over- and under-expression of the corresponding IHC.

Prognosis provides patients with information to make decisions about adjuvant treatments, thus it is important in clinical medicine. For each of the 23 individual IHC, we applied the log-rank test to the 131 Asian LADC patients (Supplementary Table S3). The results showed that the estimated survival curve of patients with over-expressed IHC of RAD54B was significantly separated from that of the remaining group (*P* = 0.00044; Figure 3), and overexpressed RAD54B was correlated with poor survival. Next, of the predicted SL pairs in Table 3, patients with abnormal IHC expression (by Table 2) of each of BRCA1-RAD54B, FEN1(N)-RAD54B and PARP1-RAD54B had a significantly different survival curve than that of the remaining group (*P* = 0.00006, 0.00024, 0.00038, respectively, log-rank test; Figure 3). There were another nine IHC pairs of RAD54B, which also significantly separated the patients with poor survival from the remaining group, but their *p*-values were less significant than that of RAD54B alone.

### External confirmation of the association of RAD54B, BRCA1-RAD54B, FEN1-RAD54B and PARP1-RAD54B with poor survival

Ethnicity and geography are known to play a role in the causes of cancer. If the markers uncovered above can be confirmed by independent data sets of LADC patients from diverse geographic regions and ethnicities, then they will be useful in clinical medicine. Thus, we further analyzed two MGED sets of independent cohorts of GSE13213 (117 Japanese patients [27] and GSE68465^†^(HLM, 79 patients [28]) and RNA-seq of 230 LADC patients from the TCGA cohort (TCGA henceforth) [29], although only ~40% of genes had expression levels that correlated with those of their proteins in human. Of these sets studied, 2-fold over-expression of *RAD54B*↑ was confirmed to be significantly correlated with poor survival (*P* = 5.8×10−5, 0.017 and 0.006, respectively). Similarly, *FEN1*↑-*RAD54B*↑ and *PARP1*↑-*RAD54B*↑ (*BRCA1*↓-*RAD54B*↑) were validated by TCGA set with *P* = 0.023 and 0.027 (HLM, *P* = 0.017), respectively, where ↑ and ↓ denote 2-fold over- and under-expression of the corresponding gene. The estimated survival curves are in Supplementary Figure S4. This external validation demonstrates that these markers can be used to screen future LADC patients if their IHC, MGED or RNA-seq are available, thus, they may have wide applications.

^†^Log ratios of the genes analyzed in the UM and CAN/DF sets were also available, but their expression levels did not pass the 2-fold threshold, while there was no control in the MSK set.

### RAD54B, BRCA1-RAD54B, FEN1-RAD54B and PARP1-RAD54B are independent prognostic markers

Prognostic markers, which provide information on the likely course of cancer, can be identified from gene or protein expression data [30–31]. Here we investigated IHC of which individual proteins in Table 2 and which predicted SL pairs (Table 3) were prognostic markers in Asian LADC. A univariate Cox proportional hazard regression analysis, applied to an independent cohort of 131 Taiwanese LADC patients, suggested that IHC of RAD54B↑, BRCA1↓-RAD54B↑, FEN1(N)↑-RAD54B↑ and PARP1↑-RAD54B↑, where ↑ and ↓ denote over- and under-expression of the corresponding IHC, were prognostic markers (hazard ratio (HR) = 2.39, 2.81, 2.61 and 2.40 with *P* = 0.001, 1.2×10−4, 4.0×10−4 and 0.001, respectively; Table 4A); see Supplementary Table S4 for the results of all predicted SL pairs. Moreover, survival analysis of three clinical covariates (age, sex and stage) indicated that they were all significantly correlated with survival (HR= 2.21, 1.99 and 2.69, with *P* = 0.004, 0.006 and 0.001, respectively; Table 4A).

**Table 4:** Overall survival of 131 lung adenocarcinoma patients in relation to clinical covariates and immunohistochemistry of protein pairs

A. Univariate Cox regression			
Variable	Subset	Hazard ratio (95% CI)	*p-*value
Stage	III–IV/I–II	2.69 (1.66 - 4.37)	0.000
Age	age>65/age≦65	2.21 (1.29 - 3.78)	0.004
Sex	male/female	1.99 (1.22 - 3.26)	0.006
RAD54B	↑/↓^a^	2.39 (1.45 - 3.94)	0.001
(BRCA1(C), RAD54B)	(↓,↑)/otherwise	2.81 (1.66 - 4.75)	0.000
(FEN1(N), RAD54B)	(↑,↑)/otherwise	2.61 (1.53 - 4.43)	0.000
(PARP1(N), RAD54B)	(↑,↑)/otherwise	2.40 (1.41 - 4.08)	0.001
**B. Multivariate Cox regression**^b^			
**Variable**	**Subset**	**Hazard ratio (95% CI)**	***p-*value**
**RAD54B and three clinical factors**			
RAD54B	↑/↓	1.82 (1.09 - 3.02)	0.021
Age	age>65/age≦65	2.75 (1.55 - 4.88)	0.001
Stage	III–IV/I–II	3.08 (1.79 - 5.29)	0.000
**BRCA1-RAD54B and three clinical factors**			
(BRCA1, RAD54B)	(↓,↑)/otherwise	2.19 (1.28 - 3.73)	0.004
Age	age>65/age≦65	2.66 (1.50 - 4.75)	0.001
Stage	III–IV/I–II	3.20 (1.80 - 5.68)	0.000
**FEN1(N)-RAD54B and three clinical factors**			
(FEN1(N), RAD54B)	(↑,↑)/otherwise	2.12 (1.22 - 3.68)	0.007
Age	age>65/age≦65	2.60 (1.46 - 4.65)	0.001
Stage	III–IV/I–II	3.24 (1.89 - 5.54)	0.000
**PARP1-RAD54B and three clinical factors**			
(PARP1, RAD54B)	(↑,↑)/otherwise	1.90 (1.13 - 3.19)	0.016
Age	age>65/age≦65	2.49 (1.39 - 4.46)	0.002
Stage	III–IV/I–II	2.88 (1.66 - 4.98)	0.000

a The symbols “↑”and “↓”denote over- and under-expression of IHC, respectively of the corresponding protein.

b Variables were selected by stepwise selection and AIC.

Variance inflation factors indicated that RAD54B, BRCA1-RAD54B, FEN1(N) -RAD54B and PARP1-RAD54B may have confounding effects (collinearity) (Supplementary Table S5A), thus each of the above IHC was entered separately into a multivariate Cox regression analysis with the three clinical covariates. With stepwise variable selection and Akaike Information Criterion (AIC), a multivariate Cox regression showed that BRCA1-RAD54B, age and stage were significantly associated with the survival of LADC patients (HR = 2.19, 2.66 and 3.20 with *P* = 0.004, 0.001 and 7.5×10−5, respectively; Table 4B). Similarly, RAD54B, FEN1(N)-RAD54B and PARP1-RAD54B were also found to be significant (HR = 1.82, 2.12 and 1.90 with *P* = 0.021, 0.007 and 0.016, respectively; Table 4B), independent of age and stage. Taken together, these results showed that RAD54B, BRCA1-RAD54B, FEN1(N)-RAD54B and PARP1-RAD54B are prognostic markers independent of clinical covariates.

### Validation of the prognosis markers using external multi-site data sets

We further validated that RAD54B, BRCA1-RAD54B, FEN1(N)-RAD54B and PARP1-RAD54B were prognosis markers, using gene expression data from three independent cohorts, GSE 13213 (117 Japanese patients), HLM of GSE68465 (79 patients) and RNA-seq of 230 LADC patients from TCGA. Each of these markers with age, sex and stage were analyzed by a Multivariate Cox regression. RAD54B was confirmed by GSE13213 and TCGA (HR =15.87 and 1.94 with *P* = 4.4×10−4 and 0.024, respectively), while FEN1(N)-RAD54B and PARP1-RAD54B were validated by TCGA (HR = 1.74 and 1.83 with *P* = 0.024 and 0.016, respectively); see Supplementary Table S5B for details.

### POLB-TP53 and POLB are predictive markers for LADC patients in TCGA

Finally, we studied which individual proteins in Table 2 and the predicted SL pairs (Table 3) were predictive markers that might help select LADC patients for therapeutic strategies. We followed Shedden and colleagues [28] to preprocess UM, HLM, CAN/DF and MSK of GSE68465* (total of 443 subjects) and integrated UM and HLM as the training set to fit Multivariate Cox regression models without and with clinical covariates, then applied the fitted models to predict risk scores of the subjects in the validation sets MSK and TCGA (total of 103 and 230, respectively); CAN/DF was not applicable due to lack of stage III and IV subjects. We used 1.5-fold as the cutoff for all genes except 1.2-fold for POLB in MSK. Further details can be found in the Supplementary Methods and Results.

Of all individual and paired IHC investigated, POLB-TP53 and POLB without (with) age and stage, M1′ and M2′ (M1 and M2), were significant prognostic markers for the training set. For completeness, we also fitted Multivariate Cox regression models to the four prognostic markers for Asian LADC, namely RAD54B, BRCA1-RAD54B, FEN1-RAD54B and PARP1-RAD54B, without (with) clinical covariates M3′-M6′ (M3-M6), respectively; age, sex and stage were fitted as model M7. These 13 models fitted by the training data are in Supplementary Table S6.

**Figure 4:**
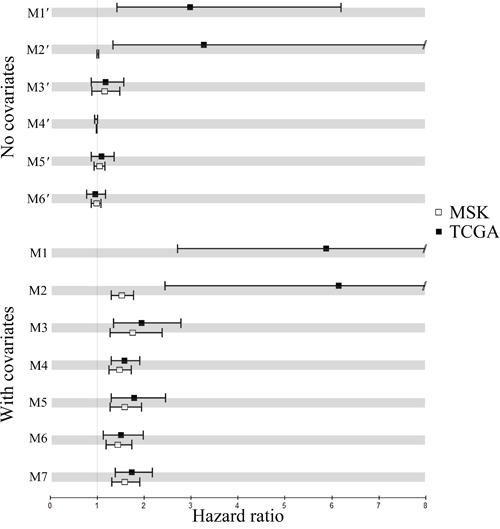
Performance of the fitted multivariate Cox models without (with) clinical covariates M1′-M6′ (M1-M6) and M7 Hazard ratios of the fitted models on two validation sets and the 95% confidence intervals.

Next, POLB-TP53 and POLB without (with) age and stage were found to be significant predictive markers for TCGA. POLB with age and stage were also predictive markers for MSK, while POLB-TP53 was not applicable to MSK, because no subjects therein had simultaneous positive expression of POLB and TP53. Additionally, we calculated the predicted risk scores of the subjects in MSK and TCGA using the fitted models M3′-M6′ and M3-M7. The estimated hazard ratios of the risk scores produced by the 13 fitted models with 95% confidence intervals (Supplementary Table S7) are depicted for MSK and TCGA in Figure 4. Hazard ratios markedly greater than 1 indicate that subjects in the validation sets with high predicted risk scores had poor outcomes. Confidence intervals not containing 1 indicate that the predictive markers performed significantly better than expected by chance.

*Biomarkers vary with race. We could only assess one set of Asian MGED with subject outcomes (GSE 13213), thus we could not study predictive markers for Asian LADC via this machine learning approach.

**Figure 5:**
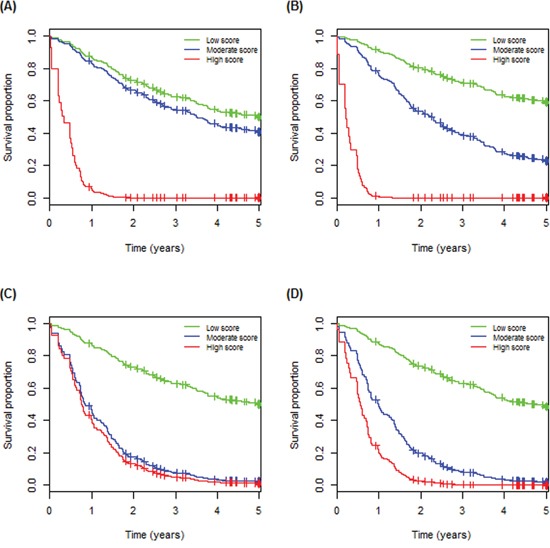
Estimated survival curves for the predictive markers on the TCGA (test) set **A.** POLB-TP53. **B.** POLB-TP53 with age and stage. **C.** POLB. **D.** POLB with age and stage.

The concordance probability estimate (CPE), which measures how well the subject outcomes agree with the predicted risk scores, for the 13 models are in Supplementary Table S8. CPE values close to 1.0 (0.5) indicate good (poor) predictivity. POLB-TP53 and POLB, the top-2 predictive markers of M1-M7, with (without) clinical covariates had CPE values 0.78 (0.71) and 0.74 (0.67) for TCGA, higher than 0.63 for the clinical covariates alone (M7). CPE of POLB with (without) clinical covariates and M7 equaled to 0.62 (0.51) and 0.62 for MSK, respectively. The estimated survival curves for POLB-TP53 and POLB with (without) age and stage on TCGA are plotted in Figure 5.

## DISCUSSION

In this study, we developed an integrated approach to reveal SL gene pairs for cancer-cell specific therapeutics and uncover prognostic and predictive markers of LADC. The novelty of our approach lies in treating clinical features as phenotypes and correlating all paired IHC of sifted proteins to the phenotypes, which resulted in the predicted SL pairs.

Of the two validated gene pairs, *PARP1-TP53* was verified to have a synergistic cytotoxicity by siRNA knockdown in H1975 and highly invasive CL1-5 lung cancer cells. To date, *cancer* recurrence and *metastasis* remain *bottlenecks in cancer treatment and* safety regarding inhibition of *TP53* is controversial [32]. Moreover, inhibitors of PARP1 *have been developed*, t*hus* PARP1 *may be a new therapeutic target for LADC, in which* TP53 *is commonly mutated (~46%) [29].*

Further, we showed that silencing PARP1 enhanced the cell death induced by the platinum-based chemotherapy drug, carboplatin, in lung cancer cells. Several reports have also found that the synthetic lethality was enhanced via combinations of PARP inhibitors and platinum-based drugs in breast cancer [33] and lung cancer [34, 35]. In addition to inhibitors of PARP1, inhibitors targeting FEN1, RAD54B and POLB have been developed, e.g., NSC-281680 and streptonigrin. Thus our results have therapeutic potential for lung cancer.

Additionally, the predicted SL pairs *FEN1-RAD54B*, *BRCA1-TP53* and *BRCA2-TP53* have been verified in colorectal cancer [24] and cervical cancer cells via RNAi knockdown [25]), respectively. Thus those gene pairs with more significant synergistic effects than these known SL pairs are also promising targets, e.g., *EGFR-RB1*, for further siRNA experiments. These results indicate that clinical factors of cancer patients may be used as phenotypes to uncover synthetic lethality between genes.

IHC of RAD54B↑, BRCA1↓-RAD54B↑, FEN1(N)↑-RAD54B↑and PARP1↑-RAD54B↑were shown to be prognostic markers, independent of age and stage, for 131 Asian LADC patients. Importantly, these markers were confirmed by three independent gene expression data sets from diverse geographic regions and ethnicities (a total of 426 LADC patients), a Japanese cohort (GSE13213, n = 117), HLM of GSE68465 and TCGA (n = 230, ~99% non-Asian subjects).

Predictive markers may help select patients for therapeutic strategies. Both POLB-TP53 and POLB were shown to be predictive markers for LADC subjects in the TCGA cohort. These markers may be used to stratify future non-Asian LADC patients.

Our approach shares the same goal as the CTD^2^ Network by NCI [36]. Both methods aim to find patient-based cancer therapeutics via tumor dependence, with the latter incorporating a network-based approach in computation. Aiming for personalized medicine, our approach can begin with pairing a patient's highly mutated genes (e.g., from targeted DNA sequencing data) with the remaining genes in the genome, then the remaining steps follow similarly.

With a rich repertoire of public domain gene expression data (e.g., TCGA), commercially available tissue arrays and matching clinical data, it is conceivable that our approach can be applied to other cancers as well. Finally, our method can be extended in the following directions. First, the initial panel of known SL pairs can be replaced by pairing a gene of interest, e.g., a frequently mutated gene in colorectal cancer such as *TP53*, with all other genes in a genome, and the remaining procedures following similarly. Second, the proposed approach can be applied to several cancers to find their common SL gene pairs for identification of common therapeutic targets.

## MATERIALS AND METHODS

### Collection of a set of 668 SL gene pairs validated in various cancer cells

We first collected 668 gene pairs whose SL interactions were either validated using various human cancer cell lines [11, 12] or genome-wide RNAi knockdown [15, 16] (Supplementary Table S1).

### Computation of gene expression profiles of lung adenocarcinoma versus non-cancerous tissues

We selected gene expression data sets using the following criteria: having both cancer and non-cancerous tissues, no treatments, no metastasis and affymetrix chips (up to Nov. 2010). The LADC gene profiles satisfying the above criteria were downloaded from the Gene Expression Omnibus database (GEO) [37]. Because mutated genes involved in the oncogenesis of a given cancer are known to vary between patients of different ethnic backgrounds [21], we collected gene expression data from Asian patients (GSE 7670 and 19804 in GEO), the same ethnic background as the IHC and clinicopathological data used in later sections. Gene expression profiles from 83 paired cancer and non-cancerous tissues in the dataset were first quantile-normalized (expresso in R), then for a given gene the log ratio of its expression in each cancer tissue versus the paired non-cancerous tissues was computed. The dataset used can be assessed via http://www.stat.sinica.edu.tw/gshieh/ladc/mged.xls.

### Inferring the initial panel of TD gene pairs for lung adenocarcinoma using microarray gene expression data

For each collected SL pair we looked for the simultaneously up-regulated pattern [abbreviated as the (up, up)], one up-regulated and one down-regulated patterns [(up, down) and (down, up)] and the simultaneously down-regulated pattern [(down, down)], where the cutoff of up- and down-regulation was 1.5-fold, owing to the 2-fold cutoff being too stringent for this data set; the fractions of these patterns of the 83 LADC patients were computed using the log ratios of gene expression data.

### Permutation test and false positive rates of the fractions of paired gene expression in Table 1

To assess the statistical significance (*p*-value) of the percentages of the (up, up) or (up, down) patterns of each gene pair in Table 1, we performed a permutation test by computing the fractions of each pattern, where the labels of 83 cancer and non-cancerous tissues were randomly exchanged 10,000 times to construct an empirical distribution, from which the *p*-value of each observed fraction was assessed. Next, we applied q-value [23] (qvalue in R) to estimate false discovery rate of the significance of the gene pairs and the predicted SL pairs in Tables 1 and 3, respectively.

### Preparation of tissue microarrays

One hundred and thirty one representative cancer specimens were chosen from hematoxylin and eosin–stained sections and confirmed by pathologists. Four tissue cores (2 mm in diameter) were obtained from each paraffin block from which three cores of cancerous and one core of noncancerous tissues were cut longitudinally. The tissue cores were set into new paraffin blocks using a fine steel needle to produce tissue microarrays. This study was approved by the Institutional Review Board and Ethics Committee of Changhua Christian Hospital, and Institutional Review Board of Academia Sinica.

### Immunohistochemistry

The sections (4 μm) of tissue microarray were deparaffinized in xylene and hydrated in serial dilutions of alcohol. Endogenous peroxidase activity was quenched by 3% H_2_O_2_. The sections were performed by treatment with boiling citrate buffer (10 mmol/L) for 20 minutes for antigen retrieval. The tissues were then incubated with 23 primary antibodies. Incubation with the primary antibody was carried out for 30 minutes at room temperature, followed by rinsing three-times with phosphate-buffered saline. The slides were incubated with a horseradish peroxidase/Fab polymer conjugate for another 30 minutes. After rinsing, the chromogen was developed with 3,3′-diamino-benzidine tetrahydrochloride as the substrate and hematoxylin as the counterstain. The staining intensity in the cancerous tissue was examined by two pathologists (Kun-Tu Yeh and Jau-Chung Hwang). The scoring criteria were similar to those in [38]. Specifically, the stain intensity was graded as negative-0, indeterminate-±, weak positive-1+, moderate positive-2+ or strong positive-3+. For staining intensity negative-0, there is no expression of the detected protein; for indeterminate-±, the staining is weak and its percentage cannot be accurately counted; for weak positive-1+, there is less than 5% expression of the detected protein in the cancer cells; for moderate positive-2+, there is focal expression in 5-20% of the cancer cells; for moderate positive-3+, there is diffuse expression over 20% of the cancer cells. Tissues with 2+ and 3+ staining of each antibody were classified as the overexpression group.

### Fisher's exact test

Fisher's exact test was conducted to determine independence between IHC expression levels of each predicted SL pair and clinicopathological factors. IHC expression levels of each protein were dichotomized into two classes, abnormal and normal, according to the criteria in Table 2. Further, clinical factors of 131 LADC patients were also dichotomized as follows; survival time < 3 years versus ≥ 3 years; tumor grade poorly-differentiated cells versus well and moderately differentiated cells; metastasis yes versus no; lymph node metastasis N1-2 versus N0; and stage III and IV versus I and II.

### Mantel-Haenszel log-rank test

For each predicted SL pair, we generated Kaplan-Meier survival curves of the high and low risk groups of patients using the software R, where the high and low risk groups comprised patients whose paired IHC expression levels were both abnormal and others, respectively. The log-rank test was used to calculate the significance of the differences between the survival curves of the two groups.

### Cell lines

The lung cancer cell lines CL1-5 were derived from *in vitro* transwell and *in vivo* metastasis selection as previously described [39]. A549 and H1975 cells were obtained from American Type Culture Collection (ATCC) (VA, USA). The CL1-5 and H1975 cells were maintained in RPMI 1640 medium supplemented with 10% fetal bovine serum while A549 cells were maintained in Dulbecco's modified eagle medium supplemented with 10% fetal bovine serum. All of the cell lines were incubated in the humidified chamber with 5% CO2 at 37°C.

### RNAi knockdown experiment

Plasmids each harboring gene-specific or scrambled shRNA were ordered from the RNAi core, Genomics Research Center, Academia Sinica. Lentivirus was prepared in accordance with standard protocols. In brief, HEK293T cells were co-transfected with pAS2neo-shRNA, pCMVΔR8.91, and pMD.G. Virus-containing medium was collected at 24-, 48-, and 72-h post-transfection, and then the virus titer was measured.

The virus titer was estimated with HEK293 cells. The A549, CL1-5 or H1975 cells were seeded in the 96-well dish (2000/well), and these cells were infected with shRNA lentivirus (MOI=2 each shRNA lentivirus, and total MOI=4) in medium containing polybrene (8 μg/ml) following the manufacturer's protocol. Post 24 h-infection, cells were refreshed with the complete medium without virus, then harvested for MTT assay, RT-PCR, or western blot analysis at 72 hours after infection.

### Cell viability assay

Cell viability was examined by MTT assay for detecting short-term cell killing effects and by colony formation assay for detecting long-term cell killing effects. Cell viability of 2000 cells with gene knockdown for 72 hours was examined by MTT assay, performed using manufacturer's protocol (Invitrogen, Eugene, OR). For colony formation assay, 250 cells with gene knockdown were cultured for 10 days. Then colonies were stained with 0.5% crystal violet in 70% ethanol and counted.

### Western blot analysis

Cells were lysed on ice for 30 min in RIPA buffer (0.5% Sodium deoxycholate, 0.1% SDS and 1% Triton X-100 in 1 x TBS) with 100 μM Na3VO4, 50 mM NaF, 30 mM Na pyrophosphate, and a protease inhibitor cocktail (Roche Diagnostics, Basel, Switzerland). Total proteins were separated by SDS-PAGE and transferred to polyvinylidene membranes (Millipore, Billerica, MA) and probed with primary antibodies. The anti-β-actin monoclonal antibody was purchased from Sigma (St. Louis, MO), anti-PARP and anti-caspase 3 antibodies were from Cell Signaling Technology (Beverly, MA), anti-p53 antibody was from Santa Cruz Biotechnology (Santa Cruz, CA). Antibodies were diluted in TBS (pH 7.5) containing 0.05% (v/v) Tween 20 and 5% non-fat milk. Blots were incubated with the appropriate horseradish peroxidase-conjugated secondary antibodies (GE Healthcare Life Sciences, Piscataway, NJ), and the bound antibodies were visualized using ECL staining.

### Reverse transcription-polymerase chain reaction

The cells infected with shLentivirus were harvested for mRNA extraction with TRIzol reagent (Thermo Fisher Scientific), and then the reverse transcription was performed. The cDNA was used to perform polymerase chain reaction. The primers used to confirm TP53 mRNA level were 5′-ACCTATGGAAACTACTTCCTGAAA-3′ and 5′-ACCATCGCTATCTGAGCAGC-3′. The primers used to confirm PARP1 mRNA level were 5′-AGCGTGTTTCTAGGTCGTGG-3′ and 5′-CCCCTTGCACGTACTTCTGT-3′.

## SUPPLEMENTARY MATERIALS AND METHODS, TABLES AND FIGURES






