# Supplementary material for: Uncovering synthetic lethal interactions for therapeutic targets and predictive markers in lung adenocarcinoma
Source: Oncotarget. 2016 Sep 15;7(45):73664–80. doi: 10.18632/oncotarget.12046 (PMC5342006; doi:10.18632/oncotarget.12046)
Supplement: Supplementary file 1 [file oncotarget-07-73664-s001.pdf]

Uncovering synthetic lethal interactions for therapeutic targets and predictive markers in lung adenocarcinoma

SUPPLEMENTARY DATA

This file includes the methods and results of (1) synergistic effect of silencing PARP1 and chemotherapy drugs by MTT assay, and (2) identification of predictive markers, which results in Supplementary Tables S6-S8 and Supplementary Figures S5-S6.

The cytotoxic effect of the combination of PARP1 silencing and some chemotherapy drugs

As suggested by a reviewer, we studied the cytotoxic effect of the combinations of PARP1 silencing and some chemotherapy drugs for lung cancer, such as carboplatin and pemetrexed. A549, CL1-5 and H1975 cells were infected with lentivirus containing shLacZ or shPARP1. Post infection, 2500 cells were treated with carboplatin or pemetrexed for 72 hours. The cell viability was analyzed by the MTT assay, and each treated shPARP1 group was compared to the shPARP1 group in Mock (after adjusted to the associated shLacZ); n = 3 per group. We found that PARP1 knockdown enhanced carboplatin and pemetrexed-induced cell death in CL1-5 and H1975 cells ($P < 0.05$; two-sample t-test), except one case had marginal significance (p-value of CL1-5 cells treated with 100 nM Pemetrexed = 0.052), but not A549 cells. Compared to pemetrexed, carboplatin may be better for NSCLC treatment (Supplementary Figure S3).

Identifying predictive markers

We followed Shedden et al. (2008) to preprocess UM, HLM, CAN/DF and MSK of GSE 68465 (total of 443 samples). Namely, we inputted .cel files to dChip, utilized quantile-normalization with GSM1672481_NCI_U133A_61L (from MSK) as control and selected default for the rest processing. We integrated UM and HLM as training data to fit six Multivariate Cox regression models without (with) clinical covariates M1′-M6′ (M1-M6); age, sex and stage were fitted as the model M7. Then we applied the fitted models to predict risk scores of subjects in two validation sets MSK and TCGA; 1.5 fold was the cutoff for differential expression of all genes in both training and test sets, except that 1.2-fold was used for POLB in MSK. CAN/DF was not used due to lack of stage III-IV patients. Concordance probability estimate (CPE), which measures how well the predicted risk scores agree with the subject outcomes, are also tabulated. Kaplan-Meier estimates of the survival function for the fitted models (M1-M7) on the test sets are plotted at the end.

SUPPLEMENTARY FIGURES AND TABLES

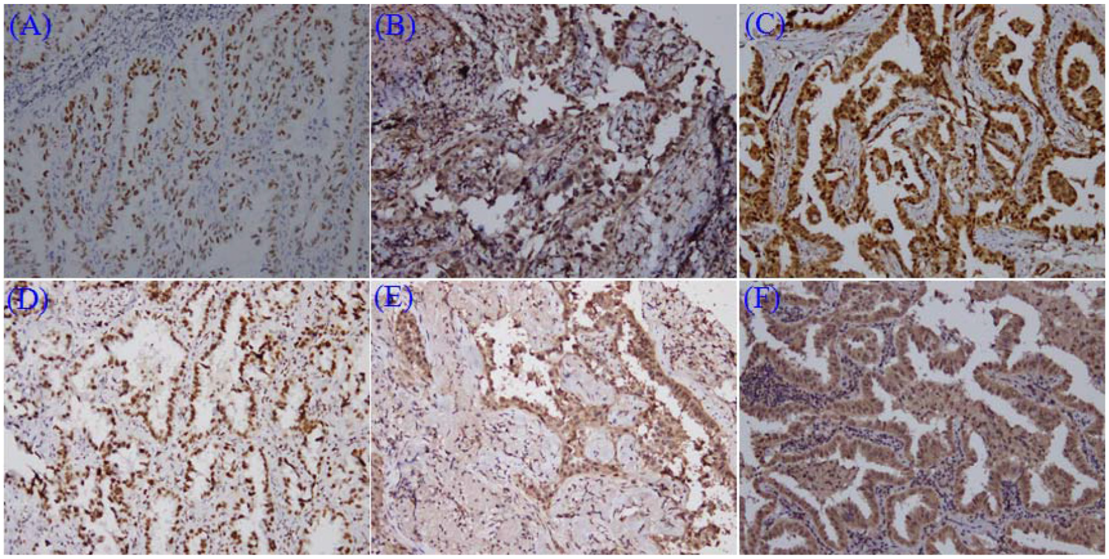

Supplementary Figure S1: Representative immunohistochemistry images of A. P53, B. RAD54B, C. FEN1, D. PARP1, E. BRCA1 and F. CSNK1E in lung adenocarcinoma (200x). A-F. Positive expression of these proteins from tumor tissues.

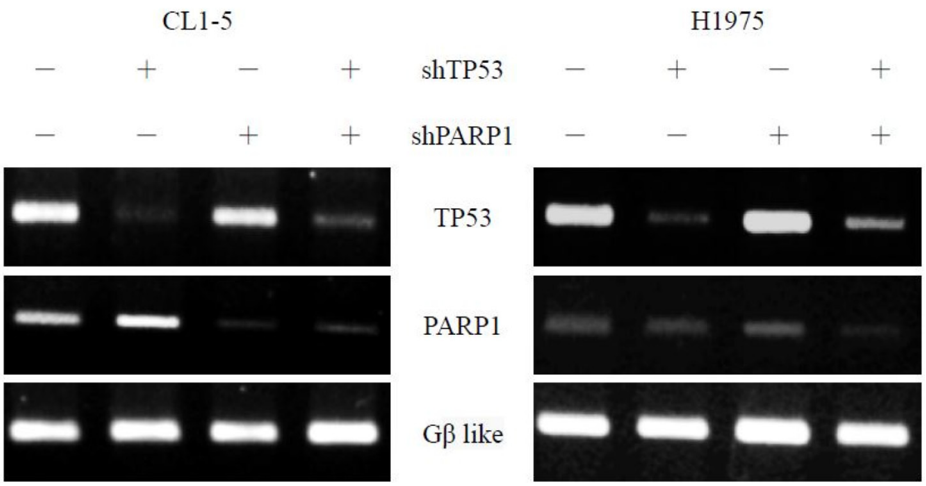

Supplementary Figure S2: The PCR graphs of two cell lines in RNAi experiments. The mRNA of CL1-5 and H1975 infected with the indicated shLentivirus was extracted, and then reversely transcribed to cDNA. The expression level of TP53 and PARP1 was detected by PCR. The upper, middle and lower panels represent the level of TP53, PARP1, and Gβ-like serving as an internal control in each cell line.

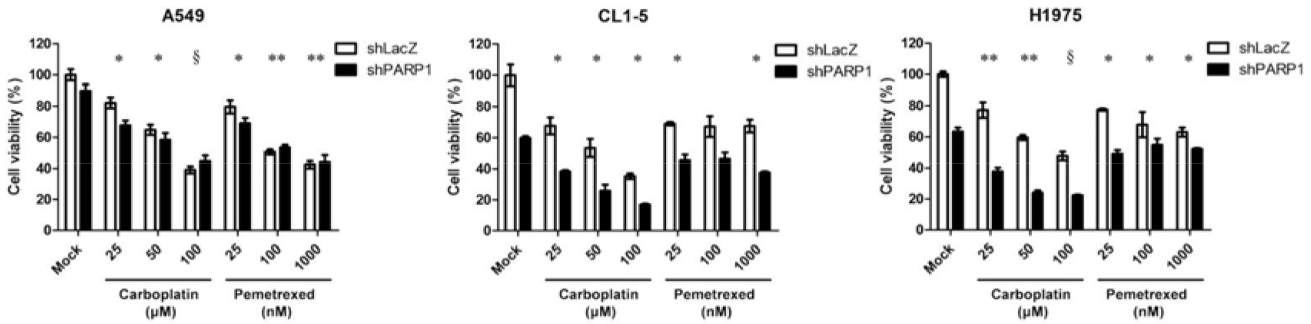

Supplementary Figure S3: PARP1 knockdown promotes the cell death induced by carboplatin and pemetrexed in CL1-5 and H1975 cells. A549, CL1-5 and H1975 cells were infected with lentivirus containing shLacZ or shPARP1. Post infection, 2500 cells were treated with carboplatin or pemetrexed for 72 hours. The cell viability was analyzed by the MTT assay and compared to the untreated shLacZ group; n = 3 per group. $^{*}P < 0.05$, $^{**}P < 0.001$ and $^{\S}P < 0.0001$ (2-sample t-test).

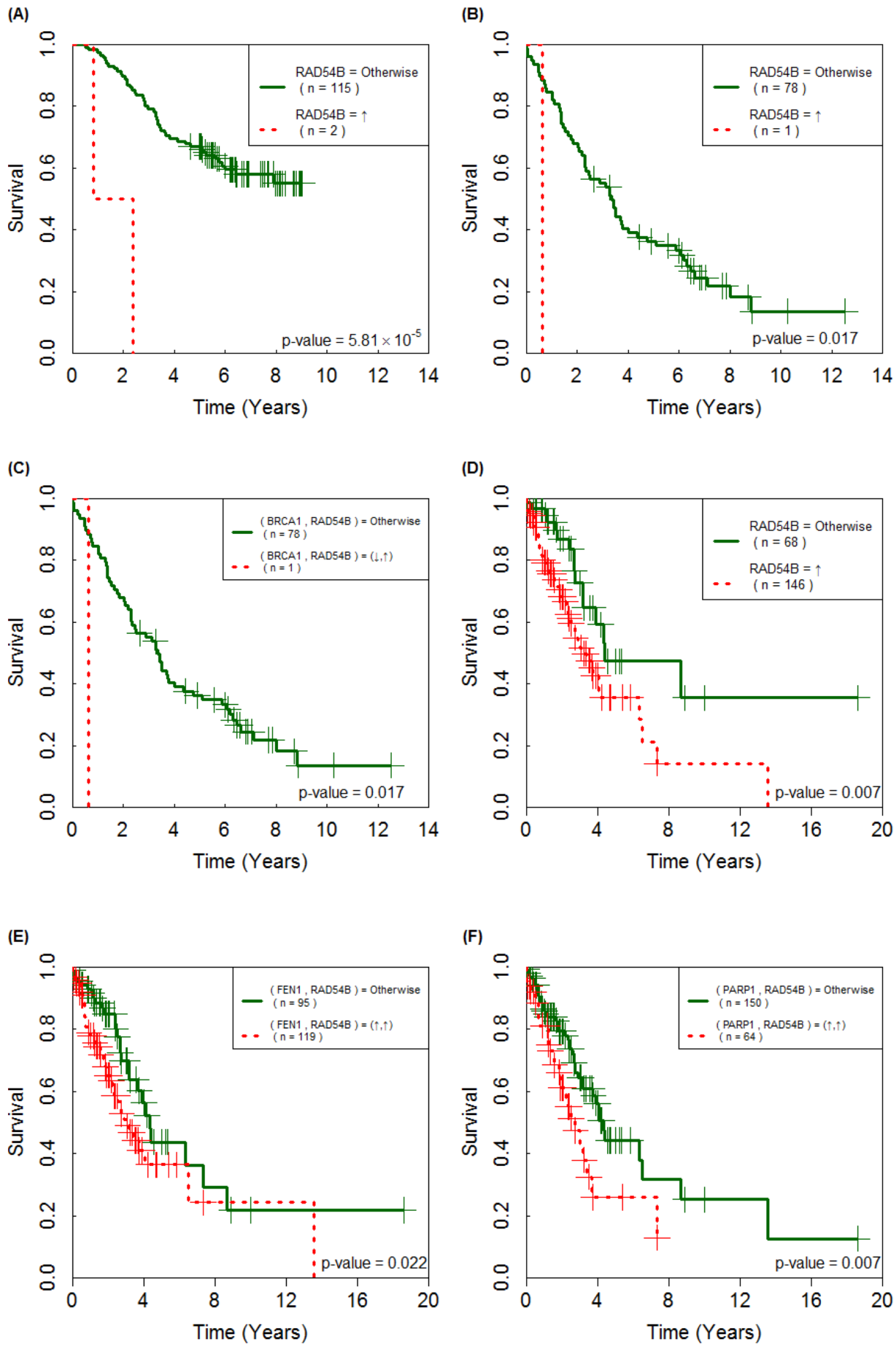

Supplementary Figure S4: Confirmation of RAD54B, BRCA1(C)-RAD54B, FEN1(N)-RAD54B and PARP1-RAD54B being prognostic markers, by three external cohorts of A. GSE 13213, **B-C.** HLM of GSE 68465 and **D-F.** TCGA data sets.

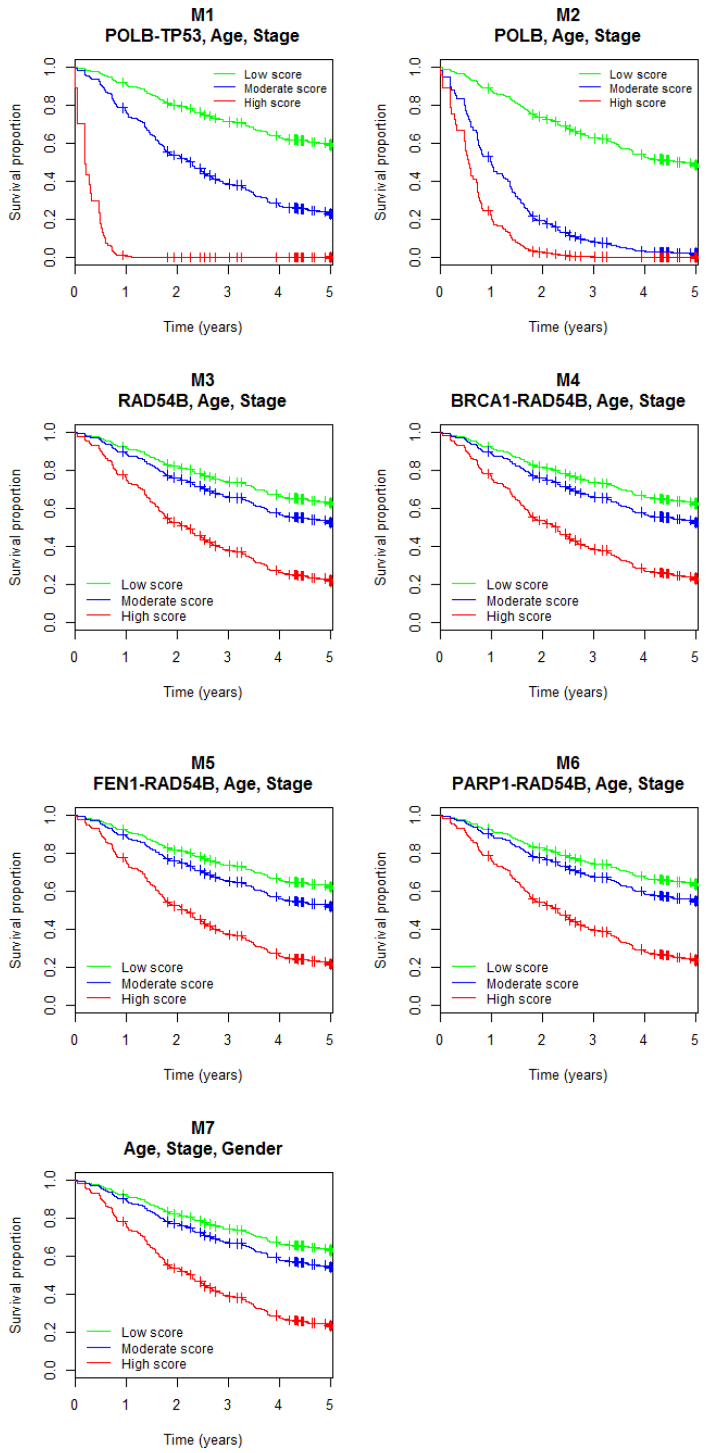

Supplementary Figure S5: Estimated survival curves for the seven fitted models (M1-M7) on TCGA subjects.

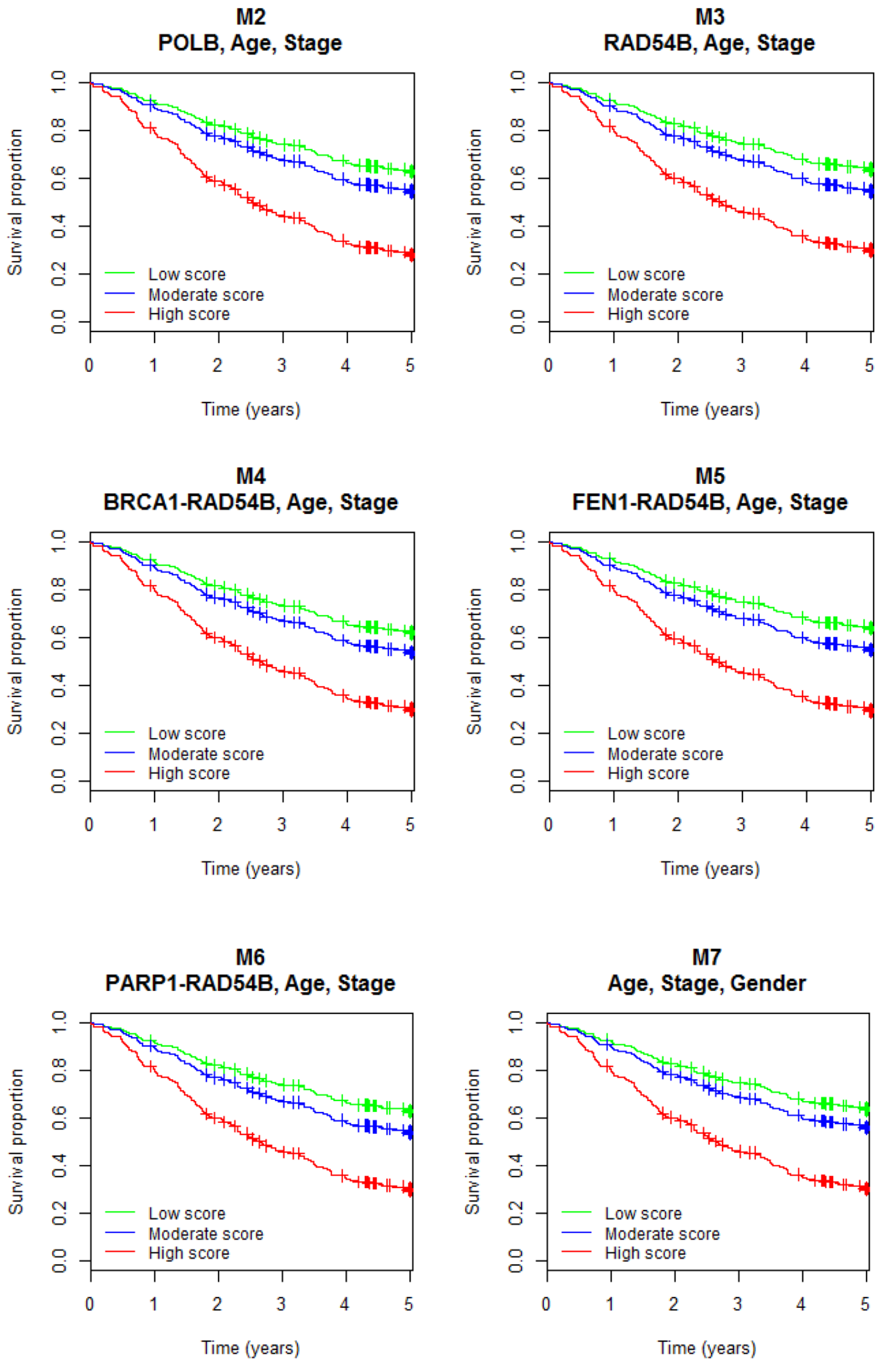

Supplementary Figure S6: Estimated survival curves for the six fitted models (M2-M7) on MSK subjects. Note that M1 could not be applied to MSK because no subject had POLB and TP53 simultaneously 1.0 fold or higher expression.

Supplementary Table S1: The 668 collected known synthetic lethal pairs

See Supplementary File S1

Supplementary Table S2: Testing 250 protein pairs for the putative synergistic correlation with the five clinical factors (sorted by p-values)

See Supplementary File S1

Supplementary Table S3: The sorted p-values of log-rank-test for the predicted synthetic lethal pairs of lung adenocarcinoma

See Supplementary File S1

Supplementary Table S4: Overall survival of 131 lung adenocarcinoma patients in relation to immunohistochemistry of the predicted synthetic lethal pairs analyzed by Univariate Cox regression; three IHC pairs predicted clinical outcome of the patients significantly ($P < 0.05$)

See Supplementary File S1

Supplementary Table S5: (A) The variance inflation factors between any two of the four markers, and (B) Adjusted hazard ratios (HRs) of the four markers by three clinical covariates, in the three external datasets

See Supplementary File S1

Supplementary Table S6: Overall survival of 256 subjects in UM and HLM in relation to GED of some predictive SL pairs and clinical covariates

A. The six fitted univariate Cox-regression models by UM and HLM

Models	Subset	Hazard ratio (95% CI)	*p*-value
**M1′:** (POLB, TP53)	(↑,↑)/otherwise	20.75 (2.70 – 159.55)	0.004
**M2′:** POLB	↑ / ↓	6.46 (1.58 – 26.50)	0.010
**M3′:** RAD54B	↑ / ↓	1.24 (0.85 – 1.80)	0.269
**M4′:** (BRCA1, RAD54B)	(↓,↑)/otherwise	0.45 (0.14 - 1.43)	0.177
**M5′:** (FEN1(N), RAD54B)	(↑,↑)/otherwise	1.14 (0.83 – 1.56)	0.419
**M6′:** (PARP1, RAD54B)	(↑,↑)/otherwise	0.93 (0.66 – 1.32)	0.695

B. The Seven fitted Multivariate Cox regression models (M1-M7)

Variable	Subset	Hazard ratio (95% CI)	*p*-value
**M1*: POLB-TP53 and two clinical covariates**			
(POLB, TP53)	(↑,↑)/otherwise	36.46 (4.67 – 284.4)	0.001
Age	age>65/age≦65	1.41 (1.02 – 1.93)	0.044
Stage	III–IV/I–II	3.43 (2.40 – 4.91)	1.5×10^{-11}
**M2: POLB and two clinical covariates**			
POLB	↑ / ↓	8.10 (1.96 – 33.54)	0.004
Age	age>65/age≦65	1.36 (0.99 – 1.87)	0.055
Stage	III–IV/I–II	3.47 (2.42 – 4.97)	1.1×10^{-11}
**M3: RAD54B and two clinical covariates**			
RAD54B	↑ / ↓	1.25 (0.86 – 1.82)	0.244
Age	age>65/age≦65	1.41 (1.03 – 1.94)	0.034
Stage	III–IV/I–II	3.37 (2.36 – 4.83)	2.9×10^{-11}
**M4: BRCA1-RAD54B and two clinical covariates**			
(BRCA1, RAD54B)	(↓,↑)/otherwise	0.59 (0.19 – 1.89)	0.378
Age	age>65/age≦65	1.37 (0.99 – 1.87)	0.054
Stage	III–IV/I–II	3.34 (2.34 – 4.78)	4.0×10^{-11}
**M5: FEN1(N)-RAD54B and two clinical covariates**			
(FEN1(N), RAD54B)	(↑,↑)/otherwise	1.13 (0.82 – 1.56)	0.441
Age	age>65/age≦65	1.42 (1.03 – 1.97)	0.032
Stage	III–IV/I–II	3.34 (2.33 – 4.78)	5.1×10^{-11}
**M6: PARP1-RAD54B and two clinical covariates**			
(PARP1, RAD54B)	(↑,↑)/otherwise	0.88 (0.62 – 1.25)	0.474
Age	age>65/age≦65	1.38 (1.01 – 1.90)	0.044
Stage	III–IV/I–II	3.42 (2.39 – 4.89)	1.8×10^{-11}
**M7: Three clinical covariates**			
Age	age>65/age≦65	1.38 (1.01 – 1.89)	0.045
Stage	III–IV/I–II	3.26 (2.27 – 4.69)	1.6×10^{-10}
sex	Male/Female	1.21 (0.88 – 1.66)	0.251

*Note that sex was not significant in M1-M6, thus it was removed.

Next, we used the fitted models (M1-M7 and M1′-M6′) to predict risk scores of subjects in two test sets (TCGA and MSK). In TCGA, 214 samples had RNA-seq and overall survival, but only 208 (103 of MSK) had additional clinical covariates age, sex and stage.

Supplementary Table S7: The hazard ratios (HRs) and its 95% confidence interval (CI) of the predicted models on two test sets (TCGA and MSK)

The formula of 95% CI for HR: $\exp\left(\log(\mathrm{HR}) \pm 1.96\sqrt{\hat{\mathrm{var}}\left(\log(\mathrm{HR})\right)}\right)$

HR (CI)	TCGA	MSK	HR (CI)	TCGA	MSK
M1	5.88 (2.71 – 12.77)	–	M1′	2.98 (1.43 – 6.20)	–
M2	6.15 (2.44 – 15.50)	1.52* (1.30 – 1.78)	M2′	3.27 (1.34 – 8.01)	1.02* (1.00 – 1.03)
M3	1.95 (1.36 – 2.79)	1.75 (1.28 – 2.39)	M3′	1.18 (0.88 – 1.58)	1.15 (0.89 – 1.49)
M4	1.58 (1.31 – 1.92)	1.47 (1.25 – 1.73)	M4′	0.98 (0.96 – 1.04)	0.99 (0.98 – 1.00)
M5	1.79 (1.31 – 2.45)	1.58 (1.28 – 1.95)	M5′	1.09 (0.88 – 1.36)	1.05 (0.94 – 1.18)
M6	1.50 (1.14 – 1.98)	1.44 (1.19 – 1.74)	M6′	0.96 (0.78 – 1.18)	0.98 (0.88 – 1.09)
M7	1.74 (1.39 – 2.18)	1.58 (1.31 – 1.91)	–	–	–

*1.2 fold was used as the cutoff for differential expression.

Supplementary Table S8: Concordance probability estimate (CPE) and standard error (se) of the fitted models (with and without clinical covariates) on two validation sets

CPE (se)	TCGA	MSK	CPE (se)	TCGA	MSK
M1	0.78 (0.02)	–	M1′	0.71 (0.02)	–
M2	0.74 (0.04)	0.62* (0.02)	M2′	0.67 (0.04)	0.51* (0.01)
M3	0.64 (0.02)	0.62 (0.02)	M3′	0.52 (0.02)	0.52 (0.02)
M4	0.63 (0.02)	0.61 (0.02)	M4′	0.51 (0.01)	0.50 (0.004)
M5	0.63 (0.02)	0.62 (0.02)	M5′	0.51 (0.02)	0.51 (0.02)
M6	0.63 (0.02)	0.62 (0.02)	M6′	0.51 (0.02)	0.51 (0.02)
M7	0.63 (0.02)	0.62 (0.02)	–	–	–

*We used 1.2 fold as the cutoff for differential expression. '–' denotes that M1 did not apply to MSK, because none in MSK had POLB and TP53 simultaneously 1.0-fold or higher expression.
CPE measures how well the subject outcomes agree with the predicted risk scores.

Supplementary Dataset S1: The clinical data and immunohistochemistry of 23 proteins of 131 lung adenocarcinoma patients in Taiwan

See Supplementary Dataset S1
