# Supplementary material for: Uncovering synthetic lethal interactions for therapeutic targets and predictive markers in lung adenocarcinoma
Source: Oncotarget. 2016 Sep 15;7(45):73664–80. doi: 10.18632/oncotarget.12046 (PMC5342006; doi:10.18632/oncotarget.12046)
Supplement: Supplementary file 3 [file oncotarget-07-73664-s003.doc]

| **Table S1.** The 668 collected known synthetic lethal pairs. | | | | | | | | | | |
| --- | --- | --- | --- | --- | --- | --- | --- | --- | --- | --- |
|
| No. | Gene 1 | Gene 2 | | | No. | Gene 1 | Gene 2 | No. | Gene 1 | Gene 2 |
| 1 | KRAS | 0RK1 | | | 40 | KRAS | BMPR1B | 79 | KRAS | CAMK2G |
| 2 | KRAS | A2M | | | 41 | KRAS | BMPR2 | 80 | KRAS | CAMK4 |
| 3 | KRAS | ABCB5 | | | 42 | KRAS | BRAF | 81 | KRAS | CARM1 |
| 4 | KRAS | ACPT | | | 43 | KRAS | BRCA1 | 82 | KRAS | CASC5 |
| 5 | KRAS | ACSM4 | | | 44 | KRAS | BRD2 | 83 | KRAS | CATSPER1 |
| 6 | KRAS | ACTR2 | | | 45 | KRAS | BTK | 84 | KRAS | CCDC112 |
| 7 | KRAS | ACVR2A | | | 46 | KRAS | BUB1 | 85 | KRAS | CCDC120 |
| 8 | KRAS | ACVRL1 | | | 47 | KRAS | BUB1B | 86 | KRAS | CCIN |
| 9 | KRAS | ADAM22 | | | 48 | KRAS | BXDC1 | 87 | KRAS | CCNA2 |
| 10 | KRAS | ADAMTSL5 | | | 49 | KRAS | BXDC2 | 88 | KRAS | CCT2 |
| 11 | KRAS | ADCK4 | | | 50 | KRAS | BXDC5 | 89 | KRAS | CDC16 |
| 12 | KRAS | ADCY8 | | | 51 | KRAS | C11orf17 | 90 | KRAS | CDC2 |
| 13 | KRAS | ADK | | | 52 | KRAS | C11ORF36 | 91 | KRAS | CDC27 |
| 14 | KRAS | ADRBK2 | | | 53 | KRAS | C11orf56 | 92 | KRAS | CDC2L1 |
| 15 | KRAS | AK2 | | | 54 | KRAS | C11orf72 | 93 | KRAS | CDC2L5 |
| 16 | KRAS | AKAP9 | | | 55 | KRAS | C12orf56 | 94 | KRAS | CDC7 |
| 17 | KRAS | AKT2 | | | 56 | KRAS | C13orf15 | 95 | KRAS | CDC73 |
| 18 | KRAS | AKT3 | | | 57 | KRAS | C13orf28 | 96 | KRAS | CDCA8 |
| 19 | KRAS | ALKBH3 | | | 58 | KRAS | C19orf2 | 97 | KRAS | CDH1 |
| 20 | KRAS | ALKBH7 | | | 59 | KRAS | C1orf14 | 98 | KRAS | CDH5 |
| 21 | KRAS | ALPK1 | | | 60 | KRAS | C20orf121 | 99 | KRAS | CDK2 |
| 22 | KRAS | ANAPC1 | | | 61 | KRAS | C21orf45 | 100 | KRAS | CDK4 |
| 23 | KRAS | ANAPC4 | | | 62 | KRAS | C21ORF67 | 101 | KRAS | CDK5R2 |
| 24 | KRAS | ANXA5 | | | 63 | KRAS | C3orf25 | 102 | KRAS | CDK6 |
| 25 | KRAS | APPBP1 | | | 64 | KRAS | C5orf40 | 103 | KRAS | CDK7 |
| 26 | KRAS | ARD1A | | | 65 | KRAS | C6 | 104 | KRAS | CDK8 |
| 27 | KRAS | ARHGEF2 | | | 66 | KRAS | C6orf48 | 105 | KRAS | CDKL3 |
| 28 | KRAS | ARID1B | | | 67 | KRAS | C7orf42 | 106 | KRAS | CDKN1C |
| 29 | KRAS | ARL4A | | | 68 | KRAS | C9orf23 | 107 | KRAS | CDKN2C |
| 30 | KRAS | ARPC2 | | | 69 | KRAS | C9orf84 | 108 | KRAS | CDKN2D |
| 31 | KRAS | ARPC3 | | | 70 | KRAS | C9orf93 | 109 | KRAS | CHEK1 |
| 32 | KRAS | ASB16 | | | 71 | KRAS | CA12 | 110 | KRAS | CHKB |
| 33 | KRAS | ATR | | | 72 | KRAS | CACNA2D2 | 111 | KRAS | CHN1 |
| 34 | KRAS | AURKC | | | 73 | KRAS | CALM1 | 112 | KRAS | CIB3 |
| 35 | KRAS | AXL | | | 74 | KRAS | CALM2 | 113 | KRAS | CIT |
| 36 | KRAS | AXUD1 | | | 75 | KRAS | CALR | 114 | KRAS | CLCN1 |
| 37 | KRAS | BCR | | | 76 | KRAS | CAMK1 | 115 | KRAS | CLK3 |
| 38 | KRAS | BFSP1 | | | 77 | KRAS | CAMK2B | 116 | KRAS | CMPK |
| 39 | KRAS | BMP2K | | | 78 | KRAS | CAMK2D | 117 | KRAS | CNOT1 |
| No. | Gene 1 | Gene 2 | | | No. | Gene 1 | Gene 2 | No. | Gene 1 | Gene 2 |
| 118 | KRAS | CNP | | | 159 | KRAS | DYRK1B | 200 | KRAS | FLJ25006 |
| 119 | KRAS | COL4A3BP | | | 160 | KRAS | DYRK4 | 201 | KRAS | FLJ34747 |
| 120 | KRAS | COPS2 | | | 161 | KRAS | DYSF | 202 | KRAS | FLJ42709 |
| 121 | KRAS | COPS4 | | | 162 | KRAS | EDA2R | 203 | KRAS | FLJ90680 |
| 122 | KRAS | COPS5 | | | 163 | KRAS | EEF1A1 | 204 | KRAS | FLRT2 |
| 123 | KRAS | COPS8 | | | 164 | KRAS | EFNB2 | 205 | KRAS | FLT3LG |
| 124 | KRAS | COX4I2 | | | 165 | KRAS | EGLN3 | 206 | KRAS | FN3K |
| 125 | KRAS | CPNE1 | | | 166 | KRAS | EIF2AK2 | 207 | KRAS | FOXR1 |
| 126 | KRAS | CREG1 | | | 167 | KRAS | EIF3S10 | 208 | KRAS | FREM1 |
| 127 | KRAS | CRNKL1 | | | 168 | KRAS | EIF3S4 | 209 | KRAS | FRMPD1 |
| 128 | KRAS | CRNN | | | 169 | KRAS | EIF3S8 | 210 | KRAS | FXN |
| 129 | KRAS | CSNK1A1L | | | 170 | KRAS | EIF4A3 | 211 | KRAS | GAK |
| 130 | KRAS | CSNK1E | | | 171 | KRAS | ELAC2 | 212 | KRAS | GATA6 |
| 131 | KRAS | CTNNAL1 | | | 172 | KRAS | EMID1 | 213 | KRAS | GATAD2A |
| 132 | KRAS | CTNS | | | 173 | KRAS | ENPP4 | 214 | KRAS | GCM2 |
| 133 | KRAS | CTTNBP2 | | | 174 | KRAS | ENPP5 | 215 | KRAS | GDF10 |
| 134 | KRAS | CXCL16 | | | 175 | KRAS | EPHA1 | 216 | KRAS | GLI3 |
| 135 | KRAS | CXORF10 | | | 176 | KRAS | EPHA3 | 217 | KRAS | GMPPB |
| 136 | KRAS | CXORF40A | | | 177 | KRAS | EPHA6 | 218 | KRAS | GNB2L1 |
| 137 | KRAS | CYLD | | | 178 | KRAS | EPHB4 | 219 | KRAS | GNL3L |
| 138 | KRAS | CYP11B1 | | | 179 | KRAS | EPRS | 220 | KRAS | GON4L |
| 139 | KRAS | DAG1 | | | 180 | KRAS | ERBB3 | 221 | KRAS | GPR56 |
| 140 | KRAS | DAPK2 | | | 181 | KRAS | ERBB4 | 222 | KRAS | GPR81 |
| 141 | KRAS | DCLK2 | | | 182 | KRAS | ERH | 223 | KRAS | GPSM2 |
| 142 | KRAS | DCTN4 | | | 183 | KRAS | ERN1 | 224 | KRAS | GRAP2 |
| 143 | KRAS | DDX43 | | | 184 | KRAS | EXOSC10 | 225 | KRAS | GRIP2 |
| 144 | KRAS | DGCR14 | | | 185 | KRAS | EXOSC4 | 226 | KRAS | GRK1 |
| 145 | KRAS | DGKA | | | 186 | KRAS | F2RL3 | 227 | KRAS | GRK4 |
| 146 | KRAS | DGKE | | | 187 | KRAS | FAM139A | 228 | KRAS | GRK6 |
| 147 | KRAS | DGKG | | | 188 | KRAS | FANCA | 229 | KRAS | GSPT1 |
| 148 | KRAS | DGKK | | | 189 | KRAS | FASTK | 230 | KRAS | GZMB |
| 149 | KRAS | DHRS7C | | | 190 | KRAS | FBL | 231 | KRAS | HCK |
| 150 | KRAS | DHX38 | | | 191 | KRAS | FBXL11 | 232 | KRAS | HIGD2A |
| 151 | KRAS | DIDO1 | | | 192 | KRAS | FBXL20 | 233 | KRAS | HIPK1 |
| 152 | KRAS | DKC1 | | | 193 | KRAS | FBXO36 | 234 | KRAS | HIPK2 |
| 153 | KRAS | DKFZP313P036 | | | 194 | KRAS | FBXW7 | 235 | KRAS | HIPK3 |
| 154 | KRAS | DLG1 | | | 195 | KRAS | FCGR1A | 236 | KRAS | HMGN2 |
| 155 | KRAS | DNM3 | | | 196 | KRAS | FEZ2 | 239 | KRAS | HOXD1 |
| 156 | KRAS | DNTTIP1 | | | 197 | KRAS | FGFR2 | 240 | KRAS | HSD3B2 |
| 157 | KRAS | DOCK4 | | | 198 | KRAS | FIGN | 241 | KRAS | HSD3B7 |
| 158 | KRAS | DYNLL2 | | | 199 | KRAS | FLJ10213 | 242 | KRAS | HSDL2 |
| No. | Gene 1 | Gene 2 | | | No. | Gene 1 | Gene 2 | No. | Gene 1 | Gene 2 |
| 243 | KRAS | HSPA14 | | | 284 | KRAS | LCE5A | 325 | KRAS | MADD |
| 244 | KRAS | HSPA5 | | | 285 | KRAS | LCK | 326 | KRAS | MAP2K4 |
| 245 | KRAS | HSPA9 | | | 286 | KRAS | LOC100101117 | 327 | KRAS | MAP2K5 |
| 246 | KRAS | HSPB8 | | | 287 | KRAS | LOC120318 | 328 | KRAS | MAP3K11 |
| 247 | KRAS | HSPD1 | | | 288 | KRAS | LOC121906 | 329 | KRAS | MAP3K5 |
| 248 | KRAS | HUNK | | | 289 | KRAS | LOC130773 | 330 | KRAS | MAP3K8 |
| 249 | KRAS | HUWE1 | | | 290 | KRAS | LOC148145 | 331 | KRAS | MAP4K1 |
| 250 | KRAS | IARS | | | 291 | KRAS | LOC150297 | 332 | KRAS | MAPK14 |
| 251 | KRAS | IARS2 | | | 292 | KRAS | LOC196913 | 333 | KRAS | MAPK15 |
| 252 | KRAS | IBTK | | | 293 | KRAS | LOC255654 | 334 | KRAS | MAPK8IP1 |
| 253 | KRAS | IGF1R | | | 294 | KRAS | LOC283194 | 335 | KRAS | MAPK9 |
| 254 | KRAS | IKBKAP | | | 295 | KRAS | LOC283412 | 336 | KRAS | MAPKAP1 |
| 255 | KRAS | IL1RAP | | | 296 | KRAS | LOC283575 | 337 | KRAS | MAPKAPK3 |
| 256 | KRAS | ILK | | | 297 | KRAS | LOC283738 | 338 | KRAS | MAPKAPK5 |
| 257 | KRAS | INPP5D | | | 298 | KRAS | LOC284344 | 339 | KRAS | MAPKBP1 |
| 258 | KRAS | INSRR | | | 299 | KRAS | LOC284379 | 340 | KRAS | MATN2 |
| 259 | KRAS | IPMK | | | 300 | KRAS | LOC284408 | 341 | KRAS | MCPH1 |
| 260 | KRAS | IREB2 | | | 301 | KRAS | LOC285556 | 342 | KRAS | METAP2 |
| 261 | KRAS | ITK | | | 302 | KRAS | LOC339766 | 343 | KRAS | MID1 |
| 262 | KRAS | ITM2A | | | 303 | KRAS | LOC375133 | 344 | KRAS | MKNK2 |
| 263 | KRAS | ITPK1 | | | 304 | KRAS | LOC387882 | 345 | KRAS | MLF2 |
| 264 | KRAS | JAK1 | | | 305 | KRAS | LOC389435 | 346 | KRAS | MLH3 |
| 265 | KRAS | JMJD1B | | | 306 | KRAS | LOC401197 | 347 | KRAS | MLL |
| 266 | KRAS | KALRN | | | 307 | KRAS | LOC441018 | 348 | KRAS | MLZE |
| 267 | KRAS | KARS | | | 308 | KRAS | LOC441246 | 349 | KRAS | MNDA |
| 268 | KRAS | KCMF1 | | | 309 | KRAS | LOC644402 | 350 | KRAS | MOBKL2A |
| 269 | KRAS | KCNH7 | | | 310 | KRAS | LOC644717 | 351 | KRAS | MORG1 |
| 270 | KRAS | KCTD12 | | | 311 | KRAS | LOC645100 | 352 | KRAS | MPDZ |
| 271 | KRAS | KDELC1 | | | 312 | KRAS | LOC645808 | 353 | KRAS | MRPL30 |
| 272 | KRAS | KIAA0101 | | | 313 | KRAS | LOC646762 | 354 | KRAS | MRPL49 |
| 273 | KRAS | KIAA0258 | | | 314 | KRAS | LOC647039 | 355 | KRAS | MRPS36P4 |
| 274 | KRAS | KIAA0509 | | | 315 | KRAS | LOC648987 | 356 | KRAS | MSH2 |
| 275 | KRAS | KIAA1160 | | | 316 | KRAS | LOC651430 | 357 | KRAS | MSH5 |
| 276 | KRAS | KIAA1856 | | | 317 | KRAS | LOC652549 | 358 | KRAS | MST1R |
| 277 | KRAS | KIF2C | | | 318 | KRAS | LOC652826 | 359 | KRAS | MTX2 |
| 278 | KRAS | KIT | | | 319 | KRAS | LOC729890 | 360 | KRAS | MVP |
| 279 | KRAS | KLHL25 | | | 320 | KRAS | LOC730000 | 361 | KRAS | MYLIP |
| 280 | KRAS | KPRP | | | 321 | KRAS | LOC730411 | 362 | KRAS | NADK |
| 281 | KRAS | KRAS | | | 322 | KRAS | LRRC10 | 363 | KRAS | NAGS |
| 282 | KRAS | KRT32 | | | 323 | KRAS | LRRC32 | 364 | KRAS | NAP1L6 |
| 283 | KRAS | LATS1 | | | 324 | KRAS | LSM5 | 365 | KRAS | NARG1 |
| No. | Gene 1 | Gene 2 | | | No. | Gene 1 | Gene 2 | No. | Gene 1 | Gene 2 |
| 366 | KRAS | NAV3 | | | 407 | KRAS | PIM2 | 448 | KRAS | PXK |
| 367 | KRAS | NBR2 | | | 408 | KRAS | PIN1 | 449 | KRAS | QARS |
| 368 | KRAS | NCAPD2 | | | 409 | KRAS | PINK1 | 450 | KRAS | RAF1 |
| 369 | KRAS | NCL | | | 410 | KRAS | PIP4K2A | 451 | KRAS | RBKS |
| 370 | KRAS | NDUFA9 | | | 411 | KRAS | PIP5K2A | 452 | KRAS | RCHY1 |
| 371 | KRAS | NEK1 | | | 412 | KRAS | PIP5K2C | 453 | KRAS | REL |
| 372 | KRAS | NEK11 | | | 413 | KRAS | PKD2 | 454 | KRAS | RELN |
| 373 | KRAS | NF1 | | | 414 | KRAS | PKLR | 455 | KRAS | RET |
| 374 | KRAS | NLK | | | 415 | KRAS | PKN2 | 456 | KRAS | RFX4 |
| 375 | KRAS | NME3 | | | 416 | KRAS | PKN3 | 457 | KRAS | RHBDL2 |
| 376 | KRAS | NME6 | | | 417 | KRAS | PLA2G2A | 458 | KRAS | RIMS1 |
| 377 | KRAS | NME7 | | | 418 | KRAS | PLCB2 | 459 | KRAS | RIPK5 |
| 378 | KRAS | NMNAT1 | | | 419 | KRAS | PLCZ1 | 460 | KRAS | RLF |
| 379 | KRAS | NOL3 | | | 420 | KRAS | PLK1 | 461 | KRAS | RNASEL |
| 380 | KRAS | NOL5A | | | 421 | KRAS | PMS1 | 462 | KRAS | RNF40 |
| 381 | KRAS | NOL8 | | | 422 | KRAS | PNCK | 463 | KRAS | RPA1 |
| 382 | KRAS | NOLA3 | | | 423 | KRAS | PNPLA4 | 464 | KRAS | RPA2 |
| 383 | KRAS | NPM1P13 | | | 424 | KRAS | POLA1 | 465 | KRAS | RPGRIP1 |
| 384 | KRAS | NQO2 | | | 425 | KRAS | POLM | 466 | KRAS | RPS6KA1 |
| 385 | KRAS | NR1I2 | | | 426 | KRAS | POLR3B | 467 | KRAS | RPS6KB1 |
| 386 | KRAS | NR1I3 | | | 427 | KRAS | POLR3F | 468 | KRAS | RPS6KB2 |
| 387 | KRAS | NR5A1 | | | 428 | KRAS | PON2 | 469 | KRAS | RPS6KL1 |
| 388 | KRAS | NUAK1 | | | 429 | KRAS | PPARA | 470 | KRAS | RQCD1 |
| 389 | KRAS | NUAK2 | | | 430 | KRAS | PPARD | 471 | KRAS | RRP9 |
| 390 | KRAS | NUP205 | | | 431 | KRAS | PPP1R14D | 472 | KRAS | RRS1 |
| 391 | KRAS | NUP93 | | | 432 | KRAS | PPP1R8 | 473 | KRAS | RSBN1 |
| 392 | KRAS | NXF1 | | | 433 | KRAS | PPP2R5A | 474 | KRAS | RTN2 |
| 393 | KRAS | OIP5 | | | 434 | KRAS | PRKAR2B | 475 | KRAS | RXRB |
| 394 | KRAS | OR2H2 | | | 435 | KRAS | PRKCSH | 476 | KRAS | SAE1 |
| 395 | KRAS | OR4K1 | | | 436 | KRAS | PRKG1 | 477 | KRAS | SDCCAG8 |
| 396 | KRAS | OSBPL9 | | | 437 | KRAS | PRKRA | 478 | KRAS | SEC14L3 |
| 397 | KRAS | PAK1 | | | 438 | KRAS | PROM1 | 479 | KRAS | SERPINB5 |
| 398 | KRAS | PANK2 | | | 439 | KRAS | PSEN1 | 480 | KRAS | SH2D3A |
| 399 | KRAS | PANK3 | | | 440 | KRAS | PSKH2 | 481 | KRAS | SH3PXD2B |
| 400 | KRAS | PANK4 | | | 441 | KRAS | PSMA5 | 482 | KRAS | SHFM1 |
| 401 | KRAS | PARP12 | | | 442 | KRAS | PSMB5 | 483 | KRAS | SKP2 |
| 402 | KRAS | PASK | | | 443 | KRAS | PSMB6 | 484 | KRAS | SLC11A2 |
| 403 | KRAS | PCK1 | | | 444 | KRAS | PSMD12 | 485 | KRAS | SLITRK2 |
| 404 | KRAS | PHF7 | | | 445 | KRAS | PSMD14 | 486 | KRAS | SNAI2 |
| 405 | KRAS | PHKBP2 | | | 446 | KRAS | PTCH2 | 487 | KRAS | SNAP23 |
| 406 | KRAS | PIK3R2 | | | 447 | KRAS | PTPN1 | 488 | KRAS | SNRPN |
| No. | Gene 1 | Gene 2 | | | No. | Gene 1 | Gene 2 | No. | Gene 1 | Gene 2 |
| 489 | KRAS | SNW1 | | | 530 | KRAS | TRIM39 | 571 | KRAS | ZBTB5 |
| 490 | KRAS | SNX2 | | | 531 | KRAS | TRIM44 | 572 | KRAS | ZC3H10 |
| 491 | KRAS | SPAST | | | 532 | KRAS | TRPV5 | 573 | KRAS | ZC3H13 |
| 492 | KRAS | SPATA17 | | | 533 | KRAS | TSC1 | 574 | KRAS | ZC3H15 |
| 493 | KRAS | SPTBN5 | | | 534 | KRAS | TSPAN15 | 575 | KRAS | ZC3H18 |
| 494 | KRAS | SPTY2D1 | | | 535 | KRAS | TSSK1B | 576 | KRAS | ZC3HAV1L |
| 495 | KRAS | SRC | | | 536 | KRAS | TSSK2 | 577 | KRAS | ZGPAT |
| 496 | KRAS | STK17A | | | 537 | KRAS | TSSK6 | 578 | KRAS | ZMAT1 |
| 497 | KRAS | STK19 | | | 538 | KRAS | TTC21B | 579 | KRAS | ZNF260 |
| 498 | KRAS | STK25 | | | 539 | KRAS | TUBGCP4 | 580 | KRAS | ZNF584 |
| 499 | KRAS | STK35 | | | 540 | KRAS | TWIST1 | 581 | ABL1 | NFATC1 |
| 500 | KRAS | STK39 | | | 541 | KRAS | TYRO3 | 582 | ABL1 | WNT5A |
| 501 | KRAS | STK4 | | | 542 | KRAS | UBAC2 | 583 | BCR | NFATC1 |
| 502 | KRAS | STT3A | | | 543 | KRAS | UBE1 | 584 | BCR | WNT5A |
| 503 | KRAS | SUV39H2 | | | 544 | KRAS | UBE2D1 | 585 | BRCA1 | PARP1 |
| 504 | KRAS | SYPL2 | | | 545 | KRAS | UBE2I | 586 | BRCA2 | PARP1 |
| 505 | KRAS | SYT16 | | | 546 | KRAS | UBE2M | 587 | CSNK1E | CTNNB1 |
| 506 | KRAS | SYT17 | | | 547 | KRAS | UBE2R2 | 588 | FEN1 | RAD54B |
| 507 | KRAS | SYT5 | | | 548 | KRAS | UBE3B | 589 | FLT1 | CTNNB1 |
| 508 | KRAS | SYTL2 | | | 549 | KRAS | UBTF | 590 | MLH1 | POLG |
| 509 | KRAS | TAF1L | | | 550 | KRAS | UCRC | 591 | MSH2 | POLB |
| 510 | KRAS | TAS2R62P | | | 551 | KRAS | UHRF2 | 592 | MYC | AURKB |
| 511 | KRAS | TBCD | | | 552 | KRAS | ULK2 | 593 | NOTCH1 | EGFR |
| 512 | KRAS | TBK1 | | | 553 | KRAS | USP30 | 594 | PINK1 | MLH1 |
| 513 | KRAS | TEAD2 | | | 554 | KRAS | USP6 | 595 | PINK1 | MSH6 |
| 514 | KRAS | TEC | | | 555 | KRAS | USP9Y | 596 | POLG | MLH1 |
| 515 | KRAS | TGFA | | | 556 | KRAS | VHL | 597 | PTEN | PARP1 |
| 516 | KRAS | TGIF2 | | | 557 | KRAS | VNN1 | 598 | RAD51 | FRTS |
| 517 | KRAS | THOC1 | | | 558 | KRAS | VPRBP | 599 | RAD51 | HELQ |
| 518 | KRAS | TIAM1 | | | 559 | KRAS | VRK1 | 600 | RB1 | SKP2 |
| 519 | KRAS | TIMM22 | | | 560 | KRAS | VRK3 | 601 | TP53 | CSNK1E |
| 520 | KRAS | TK2 | | | 561 | KRAS | W01 | 602 | TP53 | CTNNB1 |
| 521 | KRAS | TMED10 | | | 562 | KRAS | WDR36 | 603 | TP53 | MET |
| 522 | KRAS | TMEM177 | | | 563 | KRAS | WDR37 | 604 | TP53 | MK2 |
| 523 | KRAS | TMEM48 | | | 564 | KRAS | WDSOF1 | 605 | TP53 | PAK3 |
| 524 | KRAS | TMEM49 | | | 565 | KRAS | WEE1 | 606 | TP53 | RB1 |
| 525 | KRAS | TOMM20 | | | 566 | KRAS | WFDC12 | 607 | TP53 | SGK2 |
| 526 | KRAS | TPP2 | | | 567 | KRAS | WWC1 | 608 | EGFR | ABL1 |
| 527 | KRAS | TRIB3 | | | 568 | KRAS | WWP2 | 609 | EGFR | AKT2 |
| 528 | KRAS | TRIM3 | | | 569 | KRAS | XPO1 | 610 | EGFR | ANXA6 |
| 529 | KRAS | TRIM36 | | | 570 | KRAS | YSK4 | 611 | EGFR | ARF4 |
| No. | Gene 1 | Gene 2 | | | No. | Gene 1 | Gene 2 |  |  |  |
| 612 | EGFR | ARF5 | | | 653 | EGFR | PRKCE |  |  |  |
| 613 | EGFR | ASCL2 | | | 654 | EGFR | PRKCZ |  |  |  |
| 614 | EGFR | BCAR1 | | | 655 | EGFR | PTPRF |  |  |  |
| 615 | EGFR | CALM1 | | | 656 | EGFR | RAC1 |  |  |  |
| 616 | EGFR | CBLC | | | 657 | EGFR | RAPGEF1 |  |  |  |
| 617 | EGFR | CCND1 | | | 658 | EGFR | RASA3 |  |  |  |
| 618 | EGFR | CD59 | | | 659 | EGFR | RET |  |  |  |
| 619 | EGFR | CDH3 | | | 660 | EGFR | RPS6KA5 |  |  |  |
| 620 | EGFR | CXCL12 | | | 661 | EGFR | SC4MOL |  |  |  |
| 621 | EGFR | DCN | | | 662 | EGFR | SH2D3C |  |  |  |
| 622 | EGFR | DDR2 | | | 663 | EGFR | SHC1 |  |  |  |
| 623 | EGFR | DIXDC1 | | | 664 | EGFR | SMAD2 |  |  |  |
| 624 | EGFR | DLG4 | | | 665 | EGFR | SOS2 |  |  |  |
| 625 | EGFR | DUSP4 | | | 666 | EGFR | STAT3 |  |  |  |
| 626 | EGFR | DUSP6 | | | 667 | EGFR | TBL1Y |  |  |  |
| 627 | EGFR | DUSP7 | | | 668 | EGFR | VAV3 |  |  |  |
| 628 | EGFR | EPHA5 | | |  |  |  |  |  |  |
| 629 | EGFR | ERBB3 | | |  |  |  |  |  |  |
| 630 | EGFR | FER | | |  |  |  |  |  |  |
| 631 | EGFR | FGFR2 | | |  |  |  |  |  |  |
| 632 | EGFR | FLNA | | |  |  |  |  |  |  |
| 633 | EGFR | GRB7 | | |  |  |  |  |  |  |
| 634 | EGFR | HSPA9 | | |  |  |  |  |  |  |
| 635 | EGFR | INPPL1 | | |  |  |  |  |  |  |
| 636 | EGFR | KLF10 | | |  |  |  |  |  |  |
| 637 | EGFR | LOC284393 | | |  |  |  |  |  |  |
| 638 | EGFR | LOC63920 | | |  |  |  |  |  |  |
| 639 | EGFR | LTK | | |  |  |  |  |  |  |
| 640 | EGFR | MAP3K1 | | |  |  |  |  |  |  |
| 641 | EGFR | MAPK1 | | |  |  |  |  |  |  |
| 642 | EGFR | MATK | | |  |  |  |  |  |  |
| 643 | EGFR | NEDD9 | | |  |  |  |  |  |  |
| 644 | EGFR | NOTCH2 | | |  |  |  |  |  |  |
| 645 | EGFR | PIK3R1 | | |  |  |  |  |  |  |
| 646 | EGFR | | PIK3R2 |  | | | | | | |
| 647 | EGFR | | PIN1 |  | | | | | | |
| 648 | EGFR | | PKN2 |  | | | | | | |
| 649 | EGFR | | PLSCR1 |  | | | | | | |
| 650 | EGFR | | PPIA |  | | | | | | |
| 651 | EGFR | | PRKACB |  | | | | | | |
| 652 | EGFR | | PRKCD |  | | | | | | |

**Table S2.** Testing 250 protein pairs for the putative synergistic correlation with the five clinical factors (sorted by p-values).

| Protein1 | Protein2 | *p*-value | | | Log Rank | Total Number |
| --- | --- | --- | --- | --- | --- | --- |
|  |  | Protein1 | Protein2 | Protein.Pairs |  |  |
| A. Overall Survival (<3 years versus >=3 years) | | | | | | |
| FEN1(N) | RAD54B(N) | 0.357 | 0.002 | 0.001 | 0.000 | 117 |
| BRCA1(C) | RAD54B(N) | 0.442 | 0.002 | 0.001 | 0.000 | 112 |
| PARP1(N) | RAD54B(N) | 0.252 | 0.002 | 0.002 | 0.000 | 113 |
| POLB(N) | RAD54B(N) | 0.136 | 0.002 | 0.004 | 0.001 | 113 |
| ARK2(C) | RAD54B(N) | 0.261 | 0.002 | 0.005 | 0.002 | 112 |
| RAD54B(N) | SGK2(C) | 0.002 | 0.594 | 0.006 | 0.001 | 110 |
| BRCA2(C) | RAD54B(N) | 0.805 | 0.002 | 0.010 | 0.008 | 112 |
| CSNK1E(C) | RAD54B(N) | 0.986 | 0.002 | 0.010 | 0.001 | 108 |
| CDH1(M) | RAD54B(N) | 0.541 | 0.002 | 0.010 | 0.002 | 112 |
| RAD54B(N) | WNT5A(C) | 0.002 | 0.276 | 0.011 | 0.007 | 114 |
| BRCA1(C) | FEN1(N) | 0.442 | 0.357 | 0.016 | 0.056 | 113 |
| POLB(N) | SGK2(C) | 0.136 | 0.594 | 0.066 | 0.156 | 112 |
| BRCA1(C) | POLB(N) | 0.442 | 0.136 | 0.091 | 0.163 | 111 |
| RAD54B(N) | TP53(N) | 0.002 | 0.374 | 0.094 | 0.005 | 115 |
| FEN1(C) | RAD54B(N) | 0.956 | 0.002 | 0.094 | 0.048 | 117 |
| BRCA1(C) | TP53(N) | 0.442 | 0.374 | 0.098 | 0.097 | 113 |
| ARK2(C) | POLB(N) | 0.261 | 0.136 | 0.108 | 0.323 | 114 |
| ABL1(C) | SKP2(C) | 0.522 | 0.341 | 0.114 | 0.085 | 112 |
| SGK2(C) | WNT5A(C) | 0.594 | 0.276 | 0.118 | 0.333 | 112 |
| RAD54B(N) | RB1(N) | 0.002 | 0.700 | 0.123 | 0.024 | 111 |
| ARK2(C) | WNT5A(C) | 0.261 | 0.276 | 0.128 | 0.301 | 114 |
| ABL1(C) | RAD54B(N) | 0.522 | 0.002 | 0.129 | 0.009 | 112 |
| BCR(C) | SKP2(C) | 0.510 | 0.341 | 0.129 | 0.161 | 112 |
| BRCA1(C) | PARP1(N) | 0.442 | 0.252 | 0.132 | 0.163 | 111 |
| PARP1(N) | POLB(N) | 0.252 | 0.136 | 0.142 | 0.318 | 113 |
| BCR(C) | RAD54B(N) | 0.510 | 0.002 | 0.144 | 0.047 | 113 |
| CDH1(M) | FEN1(N) | 0.541 | 0.357 | 0.145 | 0.184 | 114 |
| ABL1(C) | CDH1(M) | 0.522 | 0.541 | 0.149 | 0.081 | 113 |
| TP53(N) | WNT5A(C) | 0.374 | 0.276 | 0.156 | 0.128 | 116 |
| BRCA1(C) | WNT5A(C) | 0.442 | 0.276 | 0.168 | 0.292 | 112 |
| PARP1(N) | SGK2(C) | 0.252 | 0.594 | 0.174 | 0.236 | 110 |
| ARK2(C) | PARP1(N) | 0.261 | 0.252 | 0.179 | 0.246 | 112 |
| CDH1(M) | WNT5A(C) | 0.541 | 0.276 | 0.181 | 0.627 | 114 |
| CTNNB1(N) | SKP2(C) | 0.800 | 0.341 | 0.189 | 0.198 | 112 |
| BRCA2(N) | CTNNB1(N) | 0.648 | 0.800 | 0.197 | 0.061 | 112 |
| POLB(N) | TP53(N) | 0.136 | 0.374 | 0.197 | 0.204 | 115 |
| FEN1(N) | POLB(N) | 0.357 | 0.136 | 0.197 | 0.458 | 115 |
| POLB(N) | WNT5A(C) | 0.136 | 0.276 | 0.201 | 0.545 | 115 |
| BCR(C) | CTNNB1(N) | 0.510 | 0.800 | 0.202 | 0.214 | 115 |
| PARP1(N) | SKP2(C) | 0.252 | 0.341 | 0.215 | 0.470 | 111 |
| ABL1(C) | BRCA2(C) | 0.522 | 0.805 | 0.217 | 0.282 | 110 |
| RAD54B(N) | SKP2(C) | 0.002 | 0.341 | 0.221 | 0.300 | 111 |
| FEN1(N) | WNT5A(C) | 0.357 | 0.276 | 0.221 | 0.986 | 116 |
| SGK2(C) | SKP2(C) | 0.594 | 0.341 | 0.225 | 0.480 | 112 |
| MSH2(C) | RAD54B(N) | 0.665 | 0.002 | 0.241 | 0.696 | 117 |
| ARK2(C) | SGK2(C) | 0.261 | 0.594 | 0.242 | 0.239 | 112 |
| PARP1(N) | WNT5A(C) | 0.252 | 0.276 | 0.268 | 0.720 | 113 |
| BCR(C) | SGK2(C) | 0.510 | 0.594 | 0.269 | 0.811 | 111 |
| ARK2(C) | BRCA1(C) | 0.261 | 0.442 | 0.272 | 0.307 | 111 |
| BRCA1(C) | CDH1(M) | 0.442 | 0.541 | 0.272 | 0.341 | 110 |
| PARP1(N) | RB1(N) | 0.252 | 0.700 | 0.272 | 0.140 | 111 |
| FEN1(N) | SGK2(C) | 0.357 | 0.594 | 0.276 | 0.558 | 112 |
| RB1(N) | WNT5A(C) | 0.700 | 0.276 | 0.286 | 0.351 | 113 |
| ARK2(C) | TP53(N) | 0.261 | 0.374 | 0.293 | 0.159 | 114 |
| FEN1(N) | TP53(N) | 0.357 | 0.374 | 0.294 | 0.638 | 117 |
| RB1(N) | TP53(N) | 0.700 | 0.374 | 0.299 | 0.173 | 113 |
| FEN1(N) | RB1(N) | 0.357 | 0.700 | 0.299 | 0.152 | 113 |
| ARK2(C) | FEN1(N) | 0.261 | 0.357 | 0.306 | 0.809 | 114 |
| BCR(C) | RB1(N) | 0.510 | 0.700 | 0.313 | 0.939 | 112 |
| BCR(C) | CDH1(M) | 0.510 | 0.541 | 0.313 | 0.943 | 113 |
| BCR(C) | BRCA2(C) | 0.510 | 0.805 | 0.325 | 0.760 | 112 |
| ARK2(C) | SKP2(C) | 0.261 | 0.341 | 0.328 | 0.717 | 112 |
| POLB(N) | SKP2(C) | 0.136 | 0.341 | 0.341 | 0.746 | 113 |
| CSNK1E(C) | FEN1(N) | 0.986 | 0.357 | 0.341 | 0.597 | 110 |
| BRCA2(C) | WNT5A(C) | 0.805 | 0.276 | 0.347 | 0.989 | 111 |
| BRCA2(C) | SKP2(C) | 0.805 | 0.341 | 0.348 | 0.976 | 109 |
| FEN1(N) | PARP1(N) | 0.357 | 0.252 | 0.353 | 0.869 | 114 |
| SKP2(C) | WNT5A(C) | 0.341 | 0.276 | 0.356 | 0.722 | 113 |
| MSH2(C) | TP53(N) | 0.665 | 0.374 | 0.357 | 0.538 | 117 |
| BRCA1(C) | SGK2(C) | 0.442 | 0.594 | 0.375 | 0.405 | 109 |
| FEN1(N) | MSH2(C) | 0.357 | 0.665 | 0.384 | 0.347 | 120 |
| ARK2(C) | BCR(C) | 0.261 | 0.510 | 0.385 | 0.935 | 113 |
| BRCA2(C) | FEN1(N) | 0.805 | 0.357 | 0.391 | 0.782 | 113 |
| BRCA2(N) | SKP2(C) | 0.648 | 0.341 | 0.396 | 0.275 | 109 |
| ABL1(C) | SGK2(C) | 0.522 | 0.594 | 0.397 | 0.191 | 112 |
| MYC(N) | SKP2(C) | 0.979 | 0.341 | 0.398 | 0.544 | 111 |
| ABL1(C) | BRCA2(N) | 0.522 | 0.648 | 0.404 | 0.275 | 110 |
| CDH1(M) | POLB(N) | 0.541 | 0.136 | 0.404 | 0.602 | 114 |
| FEN1(C) | WNT5A(C) | 0.956 | 0.276 | 0.405 | 0.799 | 116 |
| BRCA2(N) | RAD54B(N) | 0.648 | 0.002 | 0.406 | 0.266 | 112 |
| ABL1(C) | BCR(C) | 0.522 | 0.510 | 0.407 | 0.383 | 113 |
| ARK2(C) | BRCA2(N) | 0.261 | 0.648 | 0.408 | 0.748 | 110 |
| ABL1(C) | CTNNB1(N) | 0.522 | 0.800 | 0.413 | 0.777 | 113 |
| FEN1(N) | SKP2(C) | 0.357 | 0.341 | 0.413 | 0.868 | 113 |
| ABL1(C) | POLB(N) | 0.522 | 0.136 | 0.419 | 0.311 | 113 |
| CSNK1E(C) | WNT5A(C) | 0.986 | 0.276 | 0.432 | 0.844 | 110 |
| BCR(C) | BRCA1(C) | 0.510 | 0.442 | 0.437 | 0.957 | 112 |
| BRCA2(N) | PARP1(N) | 0.648 | 0.252 | 0.447 | 0.916 | 111 |
| ARK2(C) | CDH1(M) | 0.261 | 0.541 | 0.455 | 0.697 | 113 |
| POLB(N) | RB1(N) | 0.136 | 0.700 | 0.458 | 0.435 | 113 |
| CSNK1E(C) | SKP2(C) | 0.986 | 0.341 | 0.459 | 0.852 | 110 |
| BCR(C) | PARP1(N) | 0.510 | 0.252 | 0.460 | 0.763 | 113 |
| ABL1(C) | BRCA1(C) | 0.522 | 0.442 | 0.465 | 0.149 | 111 |
| CDH1(M) | TP53(N) | 0.541 | 0.374 | 0.475 | 0.720 | 114 |
| BRCA1(C) | FEN1(C) | 0.442 | 0.956 | 0.481 | 0.771 | 113 |
| SGK2(C) | TP53(N) | 0.594 | 0.374 | 0.481 | 0.494 | 112 |
| BCR(C) | POLB(N) | 0.510 | 0.136 | 0.485 | 0.717 | 114 |
| CDH1(M) | PARP1(N) | 0.541 | 0.252 | 0.491 | 0.766 | 112 |
| ABL1(C) | CSNK1E(C) | 0.522 | 0.986 | 0.493 | 0.270 | 110 |
| ABL1(C) | ARK2(C) | 0.522 | 0.261 | 0.499 | 0.313 | 113 |
| MYC(N) | RAD54B(N) | 0.979 | 0.002 | 0.508 | 0.378 | 110 |
| PARP1(N) | TP53(N) | 0.252 | 0.374 | 0.516 | 0.617 | 114 |
| CDH1(M) | SGK2(C) | 0.541 | 0.594 | 0.520 | 0.895 | 112 |
| BRCA2(N) | RB1(N) | 0.648 | 0.700 | 0.521 | 0.682 | 109 |
| BCR(C) | WNT5A(C) | 0.510 | 0.276 | 0.522 | 0.865 | 114 |
| BRCA2(N) | POLB(N) | 0.648 | 0.136 | 0.524 | 0.975 | 111 |
| ARK2(C) | RB1(N) | 0.261 | 0.700 | 0.525 | 0.479 | 113 |
| BRCA2(C) | POLB(N) | 0.805 | 0.136 | 0.530 | 0.643 | 111 |
| CDH1(M) | SKP2(C) | 0.541 | 0.341 | 0.545 | 0.801 | 113 |
| BCR(C) | CSNK1E(C) | 0.510 | 0.986 | 0.548 | 0.682 | 110 |
| BCR(C) | BRCA2(N) | 0.510 | 0.648 | 0.553 | 0.721 | 112 |
| BRCA2(C) | PARP1(N) | 0.805 | 0.252 | 0.566 | 0.721 | 111 |
| CSNK1E(C) | POLB(N) | 0.986 | 0.136 | 0.568 | 0.698 | 110 |
| ABL1(C) | FEN1(N) | 0.522 | 0.357 | 0.571 | 0.923 | 114 |
| BCR(C) | MYC(N) | 0.510 | 0.979 | 0.574 | 0.704 | 112 |
| BRCA1(C) | SKP2(C) | 0.442 | 0.341 | 0.576 | 0.890 | 109 |
| ABL1(C) | PARP1(N) | 0.522 | 0.252 | 0.577 | 0.439 | 112 |
| BRCA2(C) | TP53(N) | 0.805 | 0.374 | 0.591 | 0.761 | 112 |
| RB1(N) | SGK2(C) | 0.700 | 0.594 | 0.595 | 0.633 | 112 |
| EGFR(C) | RAD54B(N) | 0.931 | 0.002 | 0.602 | 0.408 | 111 |
| CDH1(M) | CSNK1E(C) | 0.541 | 0.986 | 0.615 | 0.909 | 110 |
| ABL1(C) | WNT5A(C) | 0.522 | 0.276 | 0.623 | 0.630 | 113 |
| CTNNB1(N) | FEN1(C) | 0.800 | 0.956 | 0.623 | 0.858 | 116 |
| FEN1(C) | SKP2(C) | 0.956 | 0.341 | 0.627 | 0.867 | 113 |
| BRCA2(N) | CSNK1E(C) | 0.648 | 0.986 | 0.631 | 0.780 | 107 |
| CSNK1E(C) | TP53(N) | 0.986 | 0.374 | 0.641 | 0.628 | 110 |
| MSH2(C) | POLB(N) | 0.665 | 0.136 | 0.641 | 0.980 | 115 |
| ARK2(C) | BRCA2(C) | 0.261 | 0.805 | 0.650 | 0.670 | 110 |
| CSNK1E(C) | PARP1(N) | 0.986 | 0.252 | 0.670 | 0.763 | 108 |
| BRCA1(C) | RB1(N) | 0.442 | 0.700 | 0.673 | 0.767 | 110 |
| ARK2(C) | CTNNB1(N) | 0.261 | 0.800 | 0.675 | 0.559 | 113 |
| CTNNB1(N) | PARP1(N) | 0.800 | 0.252 | 0.675 | 0.562 | 113 |
| BRCA2(N) | FEN1(C) | 0.648 | 0.956 | 0.675 | 0.935 | 113 |
| CTNNB1(N) | POLB(N) | 0.800 | 0.136 | 0.685 | 0.545 | 114 |
| BRCA2(N) | MSH2(C) | 0.648 | 0.665 | 0.687 | 0.893 | 113 |
| SKP2(C) | TP53(N) | 0.341 | 0.374 | 0.691 | 0.551 | 113 |
| BRCA2(N) | SGK2(C) | 0.648 | 0.594 | 0.693 | 0.835 | 108 |
| MSH2(N) | RAD54B(N) | 0.840 | 0.002 | 0.693 | 0.086 | 117 |
| BRCA1(C) | CSNK1E(C) | 0.442 | 0.986 | 0.694 | 0.907 | 108 |
| CDH1(M) | RB1(N) | 0.541 | 0.700 | 0.700 | 0.479 | 113 |
| ARK2(C) | CSNK1E(C) | 0.261 | 0.986 | 0.705 | 0.908 | 110 |
| BCR(C) | TP53(N) | 0.510 | 0.374 | 0.712 | 0.920 | 115 |
| ARK2(C) | MSH2(C) | 0.261 | 0.665 | 0.715 | 0.654 | 114 |
| ABL1(C) | TP53(N) | 0.522 | 0.374 | 0.718 | 0.449 | 114 |
| BRCA2(N) | MYC(N) | 0.648 | 0.979 | 0.720 | 0.408 | 109 |
| ABL1(C) | RB1(N) | 0.522 | 0.700 | 0.728 | 0.970 | 113 |
| BRCA1(C) | CTNNB1(N) | 0.442 | 0.800 | 0.730 | 0.488 | 112 |
| CTNNB1(N) | RB1(N) | 0.800 | 0.700 | 0.730 | 0.569 | 112 |
| RB1(N) | SKP2(C) | 0.700 | 0.341 | 0.730 | 0.520 | 112 |
| BCR(C) | FEN1(C) | 0.510 | 0.956 | 0.732 | 0.666 | 115 |
| MYC(N) | WNT5A(C) | 0.979 | 0.276 | 0.735 | 0.889 | 112 |
| MSH2(C) | PARP1(N) | 0.665 | 0.252 | 0.738 | 0.960 | 114 |
| BRCA2(C) | MSH2(C) | 0.805 | 0.665 | 0.739 | 0.860 | 113 |
| BRCA2(C) | CTNNB1(N) | 0.805 | 0.800 | 0.744 | 0.516 | 112 |
| CTNNB1(N) | SGK2(C) | 0.800 | 0.594 | 0.757 | 0.389 | 111 |
| CSNK1E(C) | CTNNB1(N) | 0.986 | 0.800 | 0.765 | 0.404 | 110 |
| BRCA1(C) | EGFR(C) | 0.442 | 0.931 | 0.766 | 0.976 | 110 |
| EGFR(C) | FEN1(N) | 0.931 | 0.357 | 0.769 | 0.720 | 113 |
| BRCA2(N) | WNT5A(C) | 0.648 | 0.276 | 0.774 | 0.588 | 111 |
| BRCA2(N) | MSH2(N) | 0.648 | 0.840 | 0.776 | 0.547 | 113 |
| ABL1(C) | FEN1(C) | 0.522 | 0.956 | 0.788 | 0.687 | 114 |
| CDH1(M) | MYC(N) | 0.541 | 0.979 | 0.801 | 0.809 | 112 |
| EGFR(C) | RB1(N) | 0.931 | 0.700 | 0.806 | 0.588 | 111 |
| BRCA2(C) | SGK2(C) | 0.805 | 0.594 | 0.815 | 0.317 | 108 |
| FEN1(C) | RB1(N) | 0.956 | 0.700 | 0.823 | 0.658 | 113 |
| CTNNB1(N) | MYC(N) | 0.800 | 0.979 | 0.826 | 0.438 | 112 |
| MYC(N) | RB1(N) | 0.979 | 0.700 | 0.829 | 0.744 | 112 |
| BCR(C) | EGFR(C) | 0.510 | 0.931 | 0.829 | 0.476 | 111 |
| MSH2(C) | RB1(N) | 0.665 | 0.700 | 0.833 | 0.581 | 113 |
| BRCA2(N) | FEN1(N) | 0.648 | 0.357 | 0.834 | 0.683 | 113 |
| FEN1(C) | PARP1(N) | 0.956 | 0.252 | 0.837 | 0.475 | 114 |
| BRCA2(N) | TP53(N) | 0.648 | 0.374 | 0.839 | 0.480 | 112 |
| CTNNB1(N) | TP53(N) | 0.800 | 0.374 | 0.839 | 0.481 | 115 |
| MSH2(C) | MYC(N) | 0.665 | 0.979 | 0.843 | 0.590 | 112 |
| CTNNB1(N) | FEN1(N) | 0.800 | 0.357 | 0.843 | 0.456 | 116 |
| BRCA1(C) | BRCA2(C) | 0.442 | 0.805 | 0.850 | 0.343 | 110 |
| BRCA2(C) | CSNK1E(C) | 0.805 | 0.986 | 0.856 | 0.218 | 107 |
| MSH2(N) | PARP1(N) | 0.840 | 0.252 | 0.857 | 0.693 | 114 |
| EGFR(C) | WNT5A(C) | 0.931 | 0.276 | 0.860 | 0.277 | 112 |
| CSNK1E(C) | RB1(N) | 0.986 | 0.700 | 0.860 | 0.732 | 110 |
| FEN1(C) | SGK2(C) | 0.956 | 0.594 | 0.863 | 0.426 | 112 |
| MSH2(N) | TP53(N) | 0.840 | 0.374 | 0.865 | 0.608 | 117 |
| BRCA2(C) | RB1(N) | 0.805 | 0.700 | 0.873 | 0.538 | 109 |
| ARK2(C) | MSH2(N) | 0.261 | 0.840 | 0.875 | 0.379 | 114 |
| MSH2(C) | WNT5A(C) | 0.665 | 0.276 | 0.876 | 0.317 | 116 |
| ABL1(C) | MYC(N) | 0.522 | 0.979 | 0.876 | 0.678 | 112 |
| EGFR(C) | FEN1(C) | 0.931 | 0.956 | 0.885 | 0.514 | 113 |
| MSH2(C) | SGK2(C) | 0.665 | 0.594 | 0.887 | 0.505 | 112 |
| BRCA2(N) | CDH1(M) | 0.648 | 0.541 | 0.892 | 0.302 | 110 |
| EGFR(C) | POLB(N) | 0.931 | 0.136 | 0.899 | 0.564 | 111 |
| EGFR(C) | SGK2(C) | 0.931 | 0.594 | 0.899 | 0.805 | 111 |
| EGFR(C) | TP53(N) | 0.931 | 0.374 | 0.902 | 0.927 | 113 |
| CDH1(M) | FEN1(C) | 0.541 | 0.956 | 0.904 | 0.319 | 114 |
| FEN1(C) | TP53(N) | 0.956 | 0.374 | 0.904 | 0.607 | 117 |
| BRCA1(C) | MSH2(C) | 0.442 | 0.665 | 0.905 | 0.422 | 114 |
| BRCA2(C) | MYC(N) | 0.805 | 0.979 | 0.907 | 0.511 | 109 |
| CDH1(M) | CTNNB1(N) | 0.541 | 0.800 | 0.907 | 0.334 | 113 |
| CTNNB1(N) | WNT5A(C) | 0.800 | 0.276 | 0.911 | 0.247 | 114 |
| BRCA1(C) | BRCA2(N) | 0.442 | 0.648 | 0.914 | 0.398 | 110 |
| FEN1(C) | MYC(N) | 0.956 | 0.979 | 0.918 | 0.550 | 112 |
| BRCA2(C) | CDH1(M) | 0.805 | 0.541 | 0.918 | 0.191 | 110 |
| FEN1(C) | POLB(N) | 0.956 | 0.136 | 0.921 | 0.260 | 115 |
| CSNK1E(C) | EGFR(C) | 0.986 | 0.931 | 0.924 | 0.648 | 110 |
| EGFR(C) | PARP1(N) | 0.931 | 0.252 | 0.924 | 0.637 | 110 |
| ARK2(C) | FEN1(C) | 0.261 | 0.956 | 0.930 | 0.203 | 114 |
| ARK2(C) | EGFR(C) | 0.261 | 0.931 | 0.930 | 0.602 | 111 |
| CSNK1E(C) | SGK2(C) | 0.986 | 0.594 | 0.931 | 0.672 | 110 |
| MYC(N) | POLB(N) | 0.979 | 0.136 | 0.937 | 0.375 | 112 |
| BRCA2(C) | MSH2(N) | 0.805 | 0.840 | 0.942 | 0.114 | 113 |
| MYC(N) | TP53(N) | 0.979 | 0.374 | 0.944 | 0.636 | 112 |
| MSH2(N) | POLB(N) | 0.840 | 0.136 | 0.946 | 0.329 | 115 |
| CDH1(M) | MSH2(C) | 0.541 | 0.665 | 0.946 | 0.525 | 114 |
| MYC(N) | SGK2(C) | 0.979 | 0.594 | 0.947 | 0.369 | 111 |
| BRCA2(C) | FEN1(C) | 0.805 | 0.956 | 0.950 | 0.072 | 113 |
| BCR(C) | FEN1(N) | 0.510 | 0.357 | 0.950 | 0.057 | 115 |
| CSNK1E(C) | MSH2(C) | 0.986 | 0.665 | 0.951 | 0.320 | 110 |
| CSNK1E(C) | FEN1(C) | 0.986 | 0.956 | 0.954 | 0.225 | 110 |
| BRCA1(C) | MSH2(N) | 0.442 | 0.840 | 0.959 | 0.080 | 114 |
| BRCA2(C) | EGFR(C) | 0.805 | 0.931 | 0.960 | 0.619 | 108 |
| MSH2(N) | RB1(N) | 0.840 | 0.700 | 0.962 | 0.170 | 113 |
| BRCA1(C) | MYC(N) | 0.442 | 0.979 | 0.963 | 0.402 | 110 |
| ARK2(C) | MYC(N) | 0.261 | 0.979 | 0.969 | 0.289 | 112 |
| MYC(N) | PARP1(N) | 0.979 | 0.252 | 0.974 | 0.315 | 110 |
| MSH2(N) | WNT5A(C) | 0.840 | 0.276 | 0.975 | 0.178 | 116 |
| CDH1(M) | EGFR(C) | 0.541 | 0.931 | 0.976 | 0.443 | 111 |
| FEN1(N) | MYC(N) | 0.357 | 0.979 | 0.980 | 0.067 | 112 |
| MSH2(N) | SGK2(C) | 0.840 | 0.594 | 0.985 | 0.104 | 112 |
| CSNK1E(C) | MYC(N) | 0.986 | 0.979 | 0.988 | 0.161 | 110 |
| FEN1(C) | MSH2(C) | 0.956 | 0.665 | 0.991 | 0.069 | 120 |
| EGFR(C) | MYC(N) | 0.931 | 0.979 | 0.992 | 0.315 | 110 |
| CSNK1E(C) | MSH2(N) | 0.986 | 0.840 | 0.998 | 0.038 | 110 |
| ABL1(C) | EGFR(C) | 0.522 | 0.931 | 1.000 | 0.625 | 112 |
| ABL1(C) | MSH2(C) | 0.522 | 0.665 | 1.000 | 0.223 | 114 |
| ABL1(C) | MSH2(N) | 0.522 | 0.840 | 1.000 | 0.149 | 114 |
| CTNNB1(N) | EGFR(C) | 0.800 | 0.931 | 1.000 | 0.211 | 111 |
| CTNNB1(N) | MSH2(C) | 0.800 | 0.665 | 1.000 | 0.111 | 116 |
| CTNNB1(N) | MSH2(N) | 0.800 | 0.840 | 1.000 | 0.218 | 116 |
| CTNNB1(N) | RAD54B(N) | 0.800 | 0.002 | 1.000 | 0.201 | 113 |
| BCR(C) | MSH2(C) | 0.510 | 0.665 | 1.000 | 0.137 | 115 |
| BCR(C) | MSH2(N) | 0.510 | 0.840 | 1.000 | 0.414 | 115 |
| BRCA2(N) | EGFR(C) | 0.648 | 0.931 | 1.000 | 0.289 | 108 |
| MSH2(N) | MYC(N) | 0.840 | 0.979 | 1.000 | 0.057 | 112 |
| CDH1(M) | MSH2(N) | 0.541 | 0.840 | 1.000 | 0.057 | 114 |
| EGFR(C) | MSH2(C) | 0.931 | 0.665 | 1.000 | 0.547 | 113 |
| EGFR(C) | MSH2(N) | 0.931 | 0.840 | 1.000 | 0.349 | 113 |
| EGFR(C) | SKP2(C) | 0.931 | 0.341 | 1.000 | 0.142 | 111 |
| MSH2(C) | SKP2(C) | 0.665 | 0.341 | 1.000 | 0.412 | 113 |
| FEN1(C) | MSH2(N) | 0.956 | 0.840 | 1.000 | 0.048 | 120 |
| FEN1(N) | MSH2(N) | 0.357 | 0.840 | 1.000 | 0.309 | 120 |
| MSH2(N) | SKP2(C) | 0.840 | 0.341 | NA | 0.746 | 113 |

| Protein1 | Protein2 | *p*-value | | | Log Rank | Total Number |
| --- | --- | --- | --- | --- | --- | --- |
|  |  | Protein1 | Protein2 | Protein.Pairs |  |  |
| B. Metastasis | | | | | | |
| CSNK1E(C) | TP53(N) | 0.682 | 0.012 | 0.003 | 0.628 | 119 |
| BRCA1(C) | TP53(N) | 0.281 | 0.012 | 0.005 | 0.097 | 122 |
| POLB(N) | TP53(N) | 0.485 | 0.012 | 0.007 | 0.204 | 124 |
| PARP1(N) | TP53(N) | 0.299 | 0.012 | 0.008 | 0.617 | 123 |
| RB1(N) | TP53(N) | 0.114 | 0.012 | 0.008 | 0.173 | 122 |
| SGK2(C) | TP53(N) | 0.549 | 0.012 | 0.017 | 0.494 | 121 |
| BRCA2(C) | TP53(N) | 0.778 | 0.012 | 0.019 | 0.761 | 121 |
| ARK2(C) | TP53(N) | 0.395 | 0.012 | 0.032 | 0.159 | 123 |
| BRCA1(C) | RB1(N) | 0.281 | 0.114 | 0.037 | 0.767 | 119 |
| EGFR(C) | RB1(N) | 0.233 | 0.114 | 0.052 | 0.588 | 120 |
| PARP1(N) | RB1(N) | 0.299 | 0.114 | 0.056 | 0.140 | 120 |
| RB1(N) | SGK2(C) | 0.114 | 0.549 | 0.068 | 0.633 | 121 |
| EGFR(C) | TP53(N) | 0.233 | 0.012 | 0.076 | 0.927 | 122 |
| RAD54B(N) | RB1(N) | 0.304 | 0.114 | 0.078 | 0.024 | 120 |
| RAD54B(N) | TP53(N) | 0.304 | 0.012 | 0.084 | 0.005 | 124 |
| FEN1(C) | RAD54B(N) | 0.420 | 0.304 | 0.089 | 0.048 | 126 |
| FEN1(C) | TP53(N) | 0.420 | 0.012 | 0.092 | 0.607 | 126 |
| BRCA1(C) | PARP1(N) | 0.281 | 0.299 | 0.105 | 0.163 | 120 |
| EGFR(C) | FEN1(C) | 0.233 | 0.420 | 0.112 | 0.514 | 122 |
| POLB(N) | RB1(N) | 0.485 | 0.114 | 0.134 | 0.435 | 122 |
| CDH1(M) | TP53(N) | 0.893 | 0.012 | 0.139 | 0.720 | 123 |
| ABL1(C) | BRCA1(C) | 0.436 | 0.281 | 0.141 | 0.149 | 120 |
| MYC(N) | TP53(N) | 0.576 | 0.012 | 0.156 | 0.636 | 121 |
| ARK2(C) | RB1(N) | 0.395 | 0.114 | 0.160 | 0.479 | 122 |
| FEN1(C) | PARP1(N) | 0.420 | 0.299 | 0.171 | 0.475 | 123 |
| BRCA1(C) | FEN1(C) | 0.281 | 0.420 | 0.177 | 0.771 | 122 |
| ARK2(C) | BRCA1(C) | 0.395 | 0.281 | 0.178 | 0.307 | 120 |
| BRCA1(C) | SGK2(C) | 0.281 | 0.549 | 0.182 | 0.405 | 118 |
| PARP1(N) | RAD54B(N) | 0.299 | 0.304 | 0.187 | 0.000 | 122 |
| BRCA1(C) | POLB(N) | 0.281 | 0.485 | 0.191 | 0.163 | 120 |
| PARP1(N) | POLB(N) | 0.299 | 0.485 | 0.197 | 0.318 | 122 |
| EGFR(C) | PARP1(N) | 0.233 | 0.299 | 0.197 | 0.637 | 119 |
| FEN1(C) | RB1(N) | 0.420 | 0.114 | 0.198 | 0.658 | 122 |
| CSNK1E(C) | RB1(N) | 0.682 | 0.114 | 0.200 | 0.732 | 119 |
| CSNK1E(C) | EGFR(C) | 0.682 | 0.233 | 0.210 | 0.648 | 119 |
| TP53(N) | WNT5A(C) | 0.012 | 0.565 | 0.224 | 0.128 | 125 |
| MYC(N) | RB1(N) | 0.576 | 0.114 | 0.232 | 0.744 | 121 |
| ABL1(C) | FEN1(C) | 0.436 | 0.420 | 0.235 | 0.687 | 123 |
| ABL1(C) | TP53(N) | 0.436 | 0.012 | 0.241 | 0.449 | 123 |
| ARK2(C) | RAD54B(N) | 0.395 | 0.304 | 0.242 | 0.002 | 121 |
| BRCA2(C) | RB1(N) | 0.778 | 0.114 | 0.244 | 0.538 | 118 |
| ARK2(C) | EGFR(C) | 0.395 | 0.233 | 0.250 | 0.602 | 120 |
| EGFR(C) | POLB(N) | 0.233 | 0.485 | 0.250 | 0.564 | 120 |
| CSNK1E(C) | PARP1(N) | 0.682 | 0.299 | 0.256 | 0.763 | 117 |
| EGFR(C) | FEN1(N) | 0.233 | 0.872 | 0.261 | 0.720 | 122 |
| POLB(N) | RAD54B(N) | 0.485 | 0.304 | 0.269 | 0.001 | 122 |
| RAD54B(N) | SGK2(C) | 0.304 | 0.549 | 0.277 | 0.001 | 119 |
| FEN1(C) | MYC(N) | 0.420 | 0.576 | 0.277 | 0.550 | 121 |
| FEN1(C) | POLB(N) | 0.420 | 0.485 | 0.282 | 0.260 | 124 |
| CDH1(M) | MYC(N) | 0.893 | 0.576 | 0.287 | 0.809 | 121 |
| FEN1(C) | SGK2(C) | 0.420 | 0.549 | 0.287 | 0.426 | 121 |
| PARP1(N) | SGK2(C) | 0.299 | 0.549 | 0.290 | 0.236 | 119 |
| MSH2(C) | TP53(N) | 0.820 | 0.012 | 0.296 | 0.538 | 126 |
| BRCA2(N) | RB1(N) | 0.802 | 0.114 | 0.303 | 0.682 | 118 |
| EGFR(C) | MSH2(N) | 0.233 | 0.680 | 0.310 | 0.349 | 122 |
| ABL1(C) | SGK2(C) | 0.436 | 0.549 | 0.311 | 0.191 | 121 |
| ARK2(C) | SGK2(C) | 0.395 | 0.549 | 0.315 | 0.239 | 121 |
| MSH2(C) | RAD54B(N) | 0.820 | 0.304 | 0.318 | 0.696 | 126 |
| EGFR(C) | SGK2(C) | 0.233 | 0.549 | 0.318 | 0.805 | 120 |
| BRCA1(C) | RAD54B(N) | 0.281 | 0.304 | 0.330 | 0.000 | 121 |
| BCR(C) | RB1(N) | 0.765 | 0.114 | 0.332 | 0.939 | 121 |
| FEN1(C) | WNT5A(C) | 0.420 | 0.565 | 0.333 | 0.799 | 125 |
| ABL1(C) | WNT5A(C) | 0.436 | 0.565 | 0.338 | 0.630 | 122 |
| BRCA2(C) | MYC(N) | 0.778 | 0.576 | 0.340 | 0.511 | 118 |
| MYC(N) | PARP1(N) | 0.576 | 0.299 | 0.346 | 0.315 | 119 |
| CSNK1E(C) | FEN1(C) | 0.682 | 0.420 | 0.352 | 0.225 | 119 |
| EGFR(C) | RAD54B(N) | 0.233 | 0.304 | 0.354 | 0.408 | 120 |
| BRCA1(C) | CSNK1E(C) | 0.281 | 0.682 | 0.361 | 0.907 | 117 |
| RB1(N) | WNT5A(C) | 0.114 | 0.565 | 0.362 | 0.351 | 122 |
| CSNK1E(C) | RAD54B(N) | 0.682 | 0.304 | 0.362 | 0.001 | 117 |
| BRCA1(C) | EGFR(C) | 0.281 | 0.233 | 0.376 | 0.976 | 119 |
| FEN1(N) | RB1(N) | 0.872 | 0.114 | 0.377 | 0.152 | 122 |
| MYC(N) | SGK2(C) | 0.576 | 0.549 | 0.388 | 0.369 | 120 |
| ARK2(C) | FEN1(C) | 0.395 | 0.420 | 0.394 | 0.203 | 123 |
| EGFR(C) | MYC(N) | 0.233 | 0.576 | 0.412 | 0.315 | 119 |
| ABL1(C) | CSNK1E(C) | 0.436 | 0.682 | 0.422 | 0.270 | 119 |
| MYC(N) | WNT5A(C) | 0.576 | 0.565 | 0.428 | 0.889 | 121 |
| ARK2(C) | CSNK1E(C) | 0.395 | 0.682 | 0.430 | 0.908 | 119 |
| BRCA2(N) | MSH2(C) | 0.802 | 0.820 | 0.442 | 0.893 | 122 |
| CSNK1E(C) | WNT5A(C) | 0.682 | 0.565 | 0.443 | 0.844 | 119 |
| POLB(N) | SGK2(C) | 0.485 | 0.549 | 0.448 | 0.156 | 121 |
| ABL1(C) | ARK2(C) | 0.436 | 0.395 | 0.448 | 0.313 | 122 |
| ABL1(C) | POLB(N) | 0.436 | 0.485 | 0.448 | 0.311 | 122 |
| BCR(C) | TP53(N) | 0.765 | 0.012 | 0.452 | 0.920 | 124 |
| FEN1(N) | RAD54B(N) | 0.872 | 0.304 | 0.453 | 0.000 | 126 |
| POLB(N) | WNT5A(C) | 0.485 | 0.565 | 0.453 | 0.545 | 124 |
| CSNK1E(C) | POLB(N) | 0.682 | 0.485 | 0.460 | 0.698 | 119 |
| SGK2(C) | WNT5A(C) | 0.549 | 0.565 | 0.461 | 0.333 | 121 |
| ABL1(C) | BRCA2(C) | 0.436 | 0.778 | 0.465 | 0.282 | 119 |
| MYC(N) | RAD54B(N) | 0.576 | 0.304 | 0.466 | 0.378 | 119 |
| BCR(C) | RAD54B(N) | 0.765 | 0.304 | 0.469 | 0.047 | 122 |
| BRCA1(C) | WNT5A(C) | 0.281 | 0.565 | 0.471 | 0.292 | 121 |
| RAD54B(N) | WNT5A(C) | 0.304 | 0.565 | 0.474 | 0.007 | 123 |
| MYC(N) | POLB(N) | 0.576 | 0.485 | 0.479 | 0.375 | 121 |
| ARK2(C) | WNT5A(C) | 0.395 | 0.565 | 0.481 | 0.301 | 123 |
| ARK2(C) | PARP1(N) | 0.395 | 0.299 | 0.485 | 0.246 | 121 |
| MSH2(N) | RAD54B(N) | 0.680 | 0.304 | 0.491 | 0.086 | 126 |
| PARP1(N) | WNT5A(C) | 0.299 | 0.565 | 0.503 | 0.720 | 122 |
| FEN1(N) | TP53(N) | 0.872 | 0.012 | 0.508 | 0.638 | 126 |
| ABL1(C) | PARP1(N) | 0.436 | 0.299 | 0.509 | 0.439 | 121 |
| BRCA2(C) | PARP1(N) | 0.778 | 0.299 | 0.517 | 0.721 | 120 |
| ARK2(C) | MYC(N) | 0.395 | 0.576 | 0.528 | 0.289 | 121 |
| CSNK1E(C) | SGK2(C) | 0.682 | 0.549 | 0.535 | 0.672 | 119 |
| BRCA2(N) | FEN1(N) | 0.802 | 0.872 | 0.536 | 0.683 | 122 |
| ARK2(C) | POLB(N) | 0.395 | 0.485 | 0.539 | 0.323 | 123 |
| ABL1(C) | CDH1(M) | 0.436 | 0.893 | 0.539 | 0.081 | 122 |
| BRCA2(C) | RAD54B(N) | 0.778 | 0.304 | 0.540 | 0.008 | 121 |
| CDH1(M) | RB1(N) | 0.893 | 0.114 | 0.547 | 0.479 | 122 |
| BCR(C) | EGFR(C) | 0.765 | 0.233 | 0.547 | 0.476 | 120 |
| CSNK1E(C) | MYC(N) | 0.682 | 0.576 | 0.556 | 0.161 | 119 |
| BRCA2(C) | WNT5A(C) | 0.778 | 0.565 | 0.568 | 0.989 | 120 |
| MSH2(N) | RB1(N) | 0.680 | 0.114 | 0.571 | 0.170 | 122 |
| BRCA1(C) | MYC(N) | 0.281 | 0.576 | 0.583 | 0.402 | 119 |
| MSH2(N) | TP53(N) | 0.680 | 0.012 | 0.585 | 0.608 | 126 |
| CDH1(M) | EGFR(C) | 0.893 | 0.233 | 0.602 | 0.443 | 120 |
| ARK2(C) | BRCA2(C) | 0.395 | 0.778 | 0.605 | 0.670 | 119 |
| ABL1(C) | RB1(N) | 0.436 | 0.114 | 0.612 | 0.970 | 122 |
| ABL1(C) | EGFR(C) | 0.436 | 0.233 | 0.632 | 0.625 | 121 |
| BRCA2(N) | RAD54B(N) | 0.802 | 0.304 | 0.632 | 0.266 | 121 |
| BRCA1(C) | FEN1(N) | 0.281 | 0.872 | 0.632 | 0.056 | 122 |
| ARK2(C) | MSH2(N) | 0.395 | 0.680 | 0.633 | 0.379 | 123 |
| BCR(C) | BRCA1(C) | 0.765 | 0.281 | 0.634 | 0.957 | 121 |
| BRCA2(N) | TP53(N) | 0.802 | 0.012 | 0.636 | 0.480 | 121 |
| FEN1(N) | PARP1(N) | 0.872 | 0.299 | 0.644 | 0.869 | 123 |
| EGFR(C) | WNT5A(C) | 0.233 | 0.565 | 0.658 | 0.277 | 121 |
| BCR(C) | FEN1(C) | 0.765 | 0.420 | 0.661 | 0.666 | 124 |
| ARK2(C) | BRCA2(N) | 0.395 | 0.802 | 0.674 | 0.748 | 119 |
| BRCA2(C) | FEN1(C) | 0.778 | 0.420 | 0.679 | 0.072 | 122 |
| FEN1(N) | MSH2(C) | 0.872 | 0.820 | 0.683 | 0.347 | 129 |
| BCR(C) | PARP1(N) | 0.765 | 0.299 | 0.709 | 0.763 | 122 |
| BRCA2(C) | POLB(N) | 0.778 | 0.485 | 0.709 | 0.643 | 120 |
| BCR(C) | BRCA2(C) | 0.765 | 0.778 | 0.716 | 0.760 | 121 |
| BRCA2(C) | FEN1(N) | 0.778 | 0.872 | 0.720 | 0.782 | 122 |
| ABL1(C) | BCR(C) | 0.436 | 0.765 | 0.721 | 0.383 | 122 |
| BRCA1(C) | BRCA2(C) | 0.281 | 0.778 | 0.729 | 0.343 | 119 |
| CSNK1E(C) | MSH2(N) | 0.682 | 0.680 | 0.731 | 0.038 | 119 |
| CDH1(M) | MSH2(N) | 0.893 | 0.680 | 0.739 | 0.057 | 123 |
| MSH2(N) | SGK2(C) | 0.680 | 0.549 | 0.741 | 0.104 | 121 |
| FEN1(C) | MSH2(N) | 0.420 | 0.680 | 0.742 | 0.048 | 129 |
| CDH1(M) | RAD54B(N) | 0.893 | 0.304 | 0.747 | 0.002 | 121 |
| CSNK1E(C) | FEN1(N) | 0.682 | 0.872 | 0.747 | 0.597 | 119 |
| ARK2(C) | BCR(C) | 0.395 | 0.765 | 0.749 | 0.935 | 122 |
| ABL1(C) | FEN1(N) | 0.436 | 0.872 | 0.751 | 0.923 | 123 |
| FEN1(N) | MSH2(N) | 0.872 | 0.680 | 0.757 | 0.309 | 129 |
| BCR(C) | SGK2(C) | 0.765 | 0.549 | 0.758 | 0.811 | 120 |
| FEN1(N) | POLB(N) | 0.872 | 0.485 | 0.761 | 0.458 | 124 |
| BCR(C) | WNT5A(C) | 0.765 | 0.565 | 0.761 | 0.865 | 123 |
| ABL1(C) | MYC(N) | 0.436 | 0.576 | 0.765 | 0.678 | 121 |
| CDH1(M) | WNT5A(C) | 0.893 | 0.565 | 0.771 | 0.627 | 123 |
| BRCA2(N) | SGK2(C) | 0.802 | 0.549 | 0.773 | 0.835 | 117 |
| BRCA2(N) | MSH2(N) | 0.802 | 0.680 | 0.774 | 0.547 | 122 |
| BCR(C) | POLB(N) | 0.765 | 0.485 | 0.776 | 0.717 | 123 |
| BRCA1(C) | BRCA2(N) | 0.281 | 0.802 | 0.780 | 0.398 | 119 |
| CDH1(M) | FEN1(C) | 0.893 | 0.420 | 0.789 | 0.319 | 123 |
| MSH2(N) | POLB(N) | 0.680 | 0.485 | 0.790 | 0.329 | 124 |
| ARK2(C) | FEN1(N) | 0.395 | 0.872 | 0.793 | 0.809 | 123 |
| BRCA2(C) | SGK2(C) | 0.778 | 0.549 | 0.798 | 0.317 | 117 |
| BRCA1(C) | CDH1(M) | 0.281 | 0.893 | 0.801 | 0.341 | 119 |
| CDH1(M) | CSNK1E(C) | 0.893 | 0.682 | 0.805 | 0.909 | 119 |
| CDH1(M) | POLB(N) | 0.893 | 0.485 | 0.809 | 0.602 | 123 |
| MSH2(C) | RB1(N) | 0.820 | 0.114 | 0.812 | 0.581 | 122 |
| CDH1(M) | PARP1(N) | 0.893 | 0.299 | 0.813 | 0.766 | 121 |
| ARK2(C) | MSH2(C) | 0.395 | 0.820 | 0.813 | 0.654 | 123 |
| BRCA2(C) | EGFR(C) | 0.778 | 0.233 | 0.817 | 0.619 | 117 |
| CDH1(M) | SGK2(C) | 0.893 | 0.549 | 0.818 | 0.895 | 121 |
| MSH2(N) | PARP1(N) | 0.680 | 0.299 | 0.822 | 0.693 | 123 |
| BRCA2(N) | MYC(N) | 0.802 | 0.576 | 0.824 | 0.408 | 118 |
| RB1(N) | SKP2(C) | 0.114 | 0.986 | 0.825 | 0.520 | 121 |
| MSH2(N) | MYC(N) | 0.680 | 0.576 | 0.835 | 0.057 | 121 |
| BRCA1(C) | MSH2(N) | 0.281 | 0.680 | 0.840 | 0.080 | 123 |
| ABL1(C) | RAD54B(N) | 0.436 | 0.304 | 0.845 | 0.009 | 121 |
| BCR(C) | CSNK1E(C) | 0.765 | 0.682 | 0.863 | 0.682 | 119 |
| ARK2(C) | CDH1(M) | 0.395 | 0.893 | 0.866 | 0.697 | 122 |
| BRCA2(C) | MSH2(C) | 0.778 | 0.820 | 0.867 | 0.860 | 122 |
| BRCA2(N) | CSNK1E(C) | 0.802 | 0.682 | 0.868 | 0.780 | 116 |
| MSH2(C) | POLB(N) | 0.820 | 0.485 | 0.870 | 0.980 | 124 |
| BRCA2(C) | CSNK1E(C) | 0.778 | 0.682 | 0.871 | 0.218 | 116 |
| BRCA1(C) | MSH2(C) | 0.281 | 0.820 | 0.877 | 0.422 | 123 |
| BRCA2(C) | MSH2(N) | 0.778 | 0.680 | 0.883 | 0.114 | 122 |
| FEN1(N) | WNT5A(C) | 0.872 | 0.565 | 0.886 | 0.986 | 125 |
| BRCA2(N) | POLB(N) | 0.802 | 0.485 | 0.891 | 0.975 | 120 |
| FEN1(N) | SGK2(C) | 0.872 | 0.549 | 0.896 | 0.558 | 121 |
| CSNK1E(C) | MSH2(C) | 0.682 | 0.820 | 0.909 | 0.320 | 119 |
| BCR(C) | MYC(N) | 0.765 | 0.576 | 0.909 | 0.704 | 121 |
| RAD54B(N) | SKP2(C) | 0.304 | 0.986 | 0.910 | 0.300 | 120 |
| MSH2(C) | WNT5A(C) | 0.820 | 0.565 | 0.912 | 0.317 | 125 |
| MSH2(C) | SGK2(C) | 0.820 | 0.549 | 0.915 | 0.505 | 121 |
| MSH2(C) | PARP1(N) | 0.820 | 0.299 | 0.917 | 0.960 | 123 |
| FEN1(C) | MSH2(C) | 0.420 | 0.820 | 0.919 | 0.069 | 129 |
| BRCA2(N) | PARP1(N) | 0.802 | 0.299 | 0.921 | 0.916 | 120 |
| BRCA2(C) | CDH1(M) | 0.778 | 0.893 | 0.927 | 0.191 | 119 |
| BCR(C) | CDH1(M) | 0.765 | 0.893 | 0.931 | 0.943 | 122 |
| BRCA2(N) | FEN1(C) | 0.802 | 0.420 | 0.933 | 0.935 | 122 |
| CDH1(M) | FEN1(N) | 0.893 | 0.872 | 0.936 | 0.184 | 123 |
| BRCA2(N) | WNT5A(C) | 0.802 | 0.565 | 0.937 | 0.588 | 120 |
| BCR(C) | SKP2(C) | 0.765 | 0.986 | 0.941 | 0.161 | 121 |
| BCR(C) | FEN1(N) | 0.765 | 0.872 | 0.958 | 0.057 | 124 |
| FEN1(N) | MYC(N) | 0.872 | 0.576 | 0.959 | 0.067 | 121 |
| MSH2(C) | MYC(N) | 0.820 | 0.576 | 0.964 | 0.590 | 121 |
| SKP2(C) | WNT5A(C) | 0.986 | 0.565 | 0.971 | 0.722 | 122 |
| FEN1(C) | SKP2(C) | 0.420 | 0.986 | 0.971 | 0.867 | 122 |
| BRCA1(C) | SKP2(C) | 0.281 | 0.986 | 0.972 | 0.890 | 118 |
| SGK2(C) | SKP2(C) | 0.549 | 0.986 | 0.972 | 0.480 | 121 |
| PARP1(N) | SKP2(C) | 0.299 | 0.986 | 0.976 | 0.470 | 120 |
| POLB(N) | SKP2(C) | 0.485 | 0.986 | 0.986 | 0.746 | 122 |
| ARK2(C) | SKP2(C) | 0.395 | 0.986 | 0.987 | 0.717 | 121 |
| CDH1(M) | MSH2(C) | 0.893 | 0.820 | 0.996 | 0.525 | 123 |
| ABL1(C) | CTNNB1(N) | 0.436 | 1.000 | 1.000 | 0.777 | 122 |
| ABL1(C) | BRCA2(N) | 0.436 | 0.802 | 1.000 | 0.275 | 119 |
| ABL1(C) | MSH2(C) | 0.436 | 0.820 | 1.000 | 0.223 | 123 |
| ABL1(C) | MSH2(N) | 0.436 | 0.680 | 1.000 | 0.149 | 123 |
| ABL1(C) | SKP2(C) | 0.436 | 0.986 | 1.000 | 0.085 | 121 |
| ARK2(C) | CTNNB1(N) | 0.395 | 1.000 | 1.000 | 0.559 | 122 |
| BCR(C) | CTNNB1(N) | 0.765 | 1.000 | 1.000 | 0.214 | 124 |
| BRCA1(C) | CTNNB1(N) | 0.281 | 1.000 | 1.000 | 0.488 | 121 |
| BRCA2(C) | CTNNB1(N) | 0.778 | 1.000 | 1.000 | 0.516 | 121 |
| BRCA2(N) | CTNNB1(N) | 0.802 | 1.000 | 1.000 | 0.061 | 121 |
| CSNK1E(C) | CTNNB1(N) | 0.682 | 1.000 | 1.000 | 0.404 | 119 |
| CTNNB1(N) | MYC(N) | 1.000 | 0.576 | 1.000 | 0.438 | 121 |
| CDH1(M) | CTNNB1(N) | 0.893 | 1.000 | 1.000 | 0.334 | 122 |
| CTNNB1(N) | EGFR(C) | 1.000 | 0.233 | 1.000 | 0.211 | 120 |
| CTNNB1(N) | MSH2(C) | 1.000 | 0.820 | 1.000 | 0.111 | 125 |
| CTNNB1(N) | MSH2(N) | 1.000 | 0.680 | 1.000 | 0.218 | 125 |
| CTNNB1(N) | TP53(N) | 1.000 | 0.012 | 1.000 | 0.481 | 124 |
| CTNNB1(N) | PARP1(N) | 1.000 | 0.299 | 1.000 | 0.562 | 122 |
| CTNNB1(N) | POLB(N) | 1.000 | 0.485 | 1.000 | 0.545 | 123 |
| CTNNB1(N) | RAD54B(N) | 1.000 | 0.304 | 1.000 | 0.201 | 122 |
| CTNNB1(N) | RB1(N) | 1.000 | 0.114 | 1.000 | 0.569 | 121 |
| CTNNB1(N) | SGK2(C) | 1.000 | 0.549 | 1.000 | 0.389 | 120 |
| CTNNB1(N) | SKP2(C) | 1.000 | 0.986 | 1.000 | 0.198 | 121 |
| CTNNB1(N) | WNT5A(C) | 1.000 | 0.565 | 1.000 | 0.247 | 123 |
| CTNNB1(N) | FEN1(C) | 1.000 | 0.420 | 1.000 | 0.858 | 125 |
| CTNNB1(N) | FEN1(N) | 1.000 | 0.872 | 1.000 | 0.456 | 125 |
| BCR(C) | BRCA2(N) | 0.765 | 0.802 | 1.000 | 0.721 | 121 |
| BCR(C) | MSH2(C) | 0.765 | 0.820 | 1.000 | 0.137 | 124 |
| BCR(C) | MSH2(N) | 0.765 | 0.680 | 1.000 | 0.414 | 124 |
| BRCA2(C) | SKP2(C) | 0.778 | 0.986 | 1.000 | 0.976 | 118 |
| BRCA2(N) | CDH1(M) | 0.802 | 0.893 | 1.000 | 0.302 | 119 |
| BRCA2(N) | EGFR(C) | 0.802 | 0.233 | 1.000 | 0.289 | 117 |
| BRCA2(N) | SKP2(C) | 0.802 | 0.986 | 1.000 | 0.275 | 118 |
| CSNK1E(C) | SKP2(C) | 0.682 | 0.986 | 1.000 | 0.852 | 119 |
| MYC(N) | SKP2(C) | 0.576 | 0.986 | 1.000 | 0.544 | 120 |
| CDH1(M) | SKP2(C) | 0.893 | 0.986 | 1.000 | 0.801 | 122 |
| EGFR(C) | MSH2(C) | 0.233 | 0.820 | 1.000 | 0.547 | 122 |
| EGFR(C) | SKP2(C) | 0.233 | 0.986 | 1.000 | 0.142 | 120 |
| MSH2(C) | SKP2(C) | 0.820 | 0.986 | 1.000 | 0.412 | 122 |
| MSH2(N) | WNT5A(C) | 0.680 | 0.565 | 1.000 | 0.178 | 125 |
| SKP2(C) | TP53(N) | 0.986 | 0.012 | 1.000 | 0.551 | 122 |
| FEN1(N) | SKP2(C) | 0.872 | 0.986 | 1.000 | 0.868 | 122 |
| MSH2(N) | SKP2(C) | 0.680 | 0.986 | NA | 0.746 | 122 |

| Protein1 | Protein2 | *p*-value | | | Log Rank | Total Number |
| --- | --- | --- | --- | --- | --- | --- |
|  |  | Protein1 | Protein2 | Protein.Pairs |  |  |
| C. Lymph node Metastasis | | | | | | |
| CDH1(M) | MSH2(C) | 0.472 | 0.084 | 0.007 | 0.525 | 123 |
| BRCA2(C) | MSH2(C) | 0.245 | 0.084 | 0.016 | 0.860 | 122 |
| FEN1(N) | MSH2(C) | 0.212 | 0.084 | 0.022 | 0.347 | 129 |
| BRCA1(C) | EGFR(C) | 0.355 | 0.103 | 0.033 | 0.976 | 119 |
| BRCA2(N) | TP53(N) | 0.190 | 0.351 | 0.045 | 0.480 | 121 |
| MSH2(C) | RB1(N) | 0.084 | 0.220 | 0.045 | 0.581 | 122 |
| BRCA1(C) | FEN1(N) | 0.355 | 0.212 | 0.056 | 0.056 | 122 |
| EGFR(C) | FEN1(C) | 0.103 | 0.585 | 0.057 | 0.514 | 122 |
| MSH2(C) | SGK2(C) | 0.084 | 0.539 | 0.062 | 0.505 | 121 |
| FEN1(N) | POLB(N) | 0.212 | 0.314 | 0.073 | 0.458 | 124 |
| CSNK1E(C) | MSH2(C) | 0.801 | 0.084 | 0.075 | 0.320 | 119 |
| EGFR(C) | PARP1(N) | 0.103 | 0.313 | 0.081 | 0.637 | 119 |
| EGFR(C) | RB1(N) | 0.103 | 0.220 | 0.083 | 0.588 | 120 |
| BRCA2(N) | PARP1(N) | 0.190 | 0.313 | 0.085 | 0.916 | 120 |
| FEN1(N) | RB1(N) | 0.212 | 0.220 | 0.086 | 0.152 | 122 |
| ARK2(C) | EGFR(C) | 0.968 | 0.103 | 0.088 | 0.602 | 120 |
| EGFR(C) | POLB(N) | 0.103 | 0.314 | 0.088 | 0.564 | 120 |
| BRCA2(N) | FEN1(N) | 0.190 | 0.212 | 0.089 | 0.683 | 122 |
| CDH1(M) | RB1(N) | 0.472 | 0.220 | 0.089 | 0.479 | 122 |
| BRCA2(N) | CSNK1E(C) | 0.190 | 0.801 | 0.094 | 0.780 | 116 |
| BRCA2(N) | RB1(N) | 0.190 | 0.220 | 0.095 | 0.682 | 118 |
| BCR(C) | EGFR(C) | 0.805 | 0.103 | 0.096 | 0.476 | 120 |
| BRCA2(N) | WNT5A(C) | 0.190 | 0.409 | 0.106 | 0.588 | 120 |
| BRCA2(C) | FEN1(C) | 0.245 | 0.585 | 0.111 | 0.072 | 122 |
| FEN1(N) | PARP1(N) | 0.212 | 0.313 | 0.116 | 0.869 | 123 |
| BRCA2(N) | FEN1(C) | 0.190 | 0.585 | 0.116 | 0.935 | 122 |
| FEN1(N) | SGK2(C) | 0.212 | 0.539 | 0.118 | 0.558 | 121 |
| BRCA2(C) | CDH1(M) | 0.245 | 0.472 | 0.129 | 0.191 | 119 |
| BRCA2(N) | SGK2(C) | 0.190 | 0.539 | 0.137 | 0.835 | 117 |
| CSNK1E(C) | EGFR(C) | 0.801 | 0.103 | 0.138 | 0.648 | 119 |
| MSH2(C) | PARP1(N) | 0.084 | 0.313 | 0.138 | 0.960 | 123 |
| POLB(N) | RB1(N) | 0.314 | 0.220 | 0.140 | 0.435 | 122 |
| PARP1(N) | RB1(N) | 0.313 | 0.220 | 0.149 | 0.140 | 120 |
| BRCA2(C) | FEN1(N) | 0.245 | 0.212 | 0.153 | 0.782 | 122 |
| BRCA2(N) | POLB(N) | 0.190 | 0.314 | 0.154 | 0.975 | 120 |
| BRCA2(C) | EGFR(C) | 0.245 | 0.103 | 0.154 | 0.619 | 117 |
| BRCA1(C) | RAD54B(N) | 0.355 | 0.547 | 0.163 | 0.000 | 121 |
| FEN1(C) | PARP1(N) | 0.585 | 0.313 | 0.163 | 0.475 | 123 |
| ABL1(C) | TP53(N) | 0.897 | 0.351 | 0.170 | 0.449 | 123 |
| BRCA2(C) | MSH2(N) | 0.245 | 0.432 | 0.172 | 0.114 | 122 |
| BRCA1(C) | PARP1(N) | 0.355 | 0.313 | 0.181 | 0.163 | 120 |
| PARP1(N) | POLB(N) | 0.313 | 0.314 | 0.184 | 0.318 | 122 |
| EGFR(C) | SGK2(C) | 0.103 | 0.539 | 0.185 | 0.805 | 120 |
| BRCA2(C) | TP53(N) | 0.245 | 0.351 | 0.185 | 0.761 | 121 |
| EGFR(C) | FEN1(N) | 0.103 | 0.212 | 0.191 | 0.720 | 122 |
| CDH1(M) | FEN1(N) | 0.472 | 0.212 | 0.196 | 0.184 | 123 |
| RB1(N) | SGK2(C) | 0.220 | 0.539 | 0.196 | 0.633 | 121 |
| BRCA1(C) | TP53(N) | 0.355 | 0.351 | 0.200 | 0.097 | 122 |
| SGK2(C) | TP53(N) | 0.539 | 0.351 | 0.201 | 0.494 | 121 |
| BRCA2(C) | POLB(N) | 0.245 | 0.314 | 0.202 | 0.643 | 120 |
| CSNK1E(C) | FEN1(N) | 0.801 | 0.212 | 0.204 | 0.597 | 119 |
| MSH2(C) | POLB(N) | 0.084 | 0.314 | 0.207 | 0.980 | 124 |
| BRCA1(C) | CDH1(M) | 0.355 | 0.472 | 0.218 | 0.341 | 119 |
| BRCA2(C) | PARP1(N) | 0.245 | 0.313 | 0.221 | 0.721 | 120 |
| BRCA1(C) | WNT5A(C) | 0.355 | 0.409 | 0.221 | 0.292 | 121 |
| FEN1(C) | RB1(N) | 0.585 | 0.220 | 0.223 | 0.658 | 122 |
| BRCA2(C) | WNT5A(C) | 0.245 | 0.409 | 0.225 | 0.989 | 120 |
| RB1(N) | TP53(N) | 0.220 | 0.351 | 0.231 | 0.173 | 122 |
| POLB(N) | TP53(N) | 0.314 | 0.351 | 0.232 | 0.204 | 124 |
| FEN1(C) | POLB(N) | 0.585 | 0.314 | 0.236 | 0.260 | 124 |
| BRCA1(C) | FEN1(C) | 0.355 | 0.585 | 0.247 | 0.771 | 122 |
| BRCA1(C) | MSH2(C) | 0.355 | 0.084 | 0.250 | 0.422 | 123 |
| EGFR(C) | WNT5A(C) | 0.103 | 0.409 | 0.257 | 0.277 | 121 |
| TP53(N) | WNT5A(C) | 0.351 | 0.409 | 0.258 | 0.128 | 125 |
| CSNK1E(C) | RB1(N) | 0.801 | 0.220 | 0.258 | 0.732 | 119 |
| BRCA1(C) | POLB(N) | 0.355 | 0.314 | 0.262 | 0.163 | 120 |
| BRCA1(C) | BRCA2(N) | 0.355 | 0.190 | 0.262 | 0.398 | 119 |
| BRCA2(C) | CSNK1E(C) | 0.245 | 0.801 | 0.266 | 0.218 | 116 |
| CDH1(M) | POLB(N) | 0.472 | 0.314 | 0.277 | 0.602 | 123 |
| BRCA1(C) | SGK2(C) | 0.355 | 0.539 | 0.278 | 0.405 | 118 |
| BRCA2(C) | SGK2(C) | 0.245 | 0.539 | 0.284 | 0.317 | 117 |
| BRCA1(C) | RB1(N) | 0.355 | 0.220 | 0.286 | 0.767 | 119 |
| ARK2(C) | RB1(N) | 0.968 | 0.220 | 0.293 | 0.479 | 122 |
| BRCA2(C) | RB1(N) | 0.245 | 0.220 | 0.311 | 0.538 | 118 |
| RAD54B(N) | RB1(N) | 0.547 | 0.220 | 0.312 | 0.024 | 120 |
| BRCA2(N) | CDH1(M) | 0.190 | 0.472 | 0.312 | 0.302 | 119 |
| FEN1(N) | WNT5A(C) | 0.212 | 0.409 | 0.325 | 0.986 | 125 |
| BRCA1(C) | MSH2(N) | 0.355 | 0.432 | 0.328 | 0.080 | 123 |
| PARP1(N) | SGK2(C) | 0.313 | 0.539 | 0.329 | 0.236 | 119 |
| EGFR(C) | MYC(N) | 0.103 | 0.934 | 0.331 | 0.315 | 119 |
| POLB(N) | SGK2(C) | 0.314 | 0.539 | 0.335 | 0.156 | 121 |
| BRCA2(N) | MSH2(C) | 0.190 | 0.084 | 0.336 | 0.893 | 122 |
| CDH1(M) | EGFR(C) | 0.472 | 0.103 | 0.338 | 0.443 | 120 |
| FEN1(C) | RAD54B(N) | 0.585 | 0.547 | 0.341 | 0.048 | 126 |
| FEN1(C) | MSH2(C) | 0.585 | 0.084 | 0.349 | 0.069 | 129 |
| FEN1(C) | TP53(N) | 0.585 | 0.351 | 0.350 | 0.607 | 126 |
| BRCA2(C) | RAD54B(N) | 0.245 | 0.547 | 0.358 | 0.008 | 121 |
| EGFR(C) | TP53(N) | 0.103 | 0.351 | 0.358 | 0.927 | 122 |
| RB1(N) | WNT5A(C) | 0.220 | 0.409 | 0.370 | 0.351 | 122 |
| ARK2(C) | MSH2(C) | 0.968 | 0.084 | 0.374 | 0.654 | 123 |
| RAD54B(N) | SGK2(C) | 0.547 | 0.539 | 0.374 | 0.001 | 119 |
| BRCA1(C) | BRCA2(C) | 0.355 | 0.245 | 0.385 | 0.343 | 119 |
| CDH1(M) | TP53(N) | 0.472 | 0.351 | 0.388 | 0.720 | 123 |
| CSNK1E(C) | TP53(N) | 0.801 | 0.351 | 0.393 | 0.628 | 119 |
| ARK2(C) | FEN1(C) | 0.968 | 0.585 | 0.394 | 0.203 | 123 |
| BCR(C) | FEN1(N) | 0.805 | 0.212 | 0.408 | 0.057 | 124 |
| FEN1(C) | WNT5A(C) | 0.585 | 0.409 | 0.415 | 0.799 | 125 |
| FEN1(N) | TP53(N) | 0.212 | 0.351 | 0.419 | 0.638 | 126 |
| CDH1(M) | SGK2(C) | 0.472 | 0.539 | 0.419 | 0.895 | 121 |
| BCR(C) | TP53(N) | 0.805 | 0.351 | 0.420 | 0.920 | 124 |
| FEN1(N) | RAD54B(N) | 0.212 | 0.547 | 0.422 | 0.000 | 126 |
| FEN1(C) | SGK2(C) | 0.585 | 0.539 | 0.425 | 0.426 | 121 |
| ARK2(C) | BRCA2(N) | 0.968 | 0.190 | 0.430 | 0.748 | 119 |
| RAD54B(N) | SKP2(C) | 0.547 | 0.767 | 0.444 | 0.300 | 120 |
| PARP1(N) | WNT5A(C) | 0.313 | 0.409 | 0.446 | 0.720 | 122 |
| MSH2(C) | MYC(N) | 0.084 | 0.934 | 0.448 | 0.590 | 121 |
| POLB(N) | WNT5A(C) | 0.314 | 0.409 | 0.449 | 0.545 | 124 |
| BRCA2(N) | MSH2(N) | 0.190 | 0.432 | 0.458 | 0.547 | 122 |
| MSH2(N) | MYC(N) | 0.432 | 0.934 | 0.461 | 0.057 | 121 |
| MSH2(C) | RAD54B(N) | 0.084 | 0.547 | 0.464 | 0.696 | 126 |
| BRCA2(N) | MYC(N) | 0.190 | 0.934 | 0.468 | 0.408 | 118 |
| ARK2(C) | FEN1(N) | 0.968 | 0.212 | 0.470 | 0.809 | 123 |
| EGFR(C) | SKP2(C) | 0.103 | 0.767 | 0.475 | 0.142 | 120 |
| MSH2(C) | SKP2(C) | 0.084 | 0.767 | 0.475 | 0.412 | 122 |
| EGFR(C) | RAD54B(N) | 0.103 | 0.547 | 0.476 | 0.408 | 120 |
| BRCA2(N) | RAD54B(N) | 0.190 | 0.547 | 0.481 | 0.266 | 121 |
| CDH1(M) | PARP1(N) | 0.472 | 0.313 | 0.485 | 0.766 | 121 |
| EGFR(C) | MSH2(C) | 0.103 | 0.084 | 0.488 | 0.547 | 122 |
| SGK2(C) | WNT5A(C) | 0.539 | 0.409 | 0.508 | 0.333 | 121 |
| RAD54B(N) | WNT5A(C) | 0.547 | 0.409 | 0.509 | 0.007 | 123 |
| CDH1(M) | RAD54B(N) | 0.472 | 0.547 | 0.516 | 0.002 | 121 |
| POLB(N) | RAD54B(N) | 0.314 | 0.547 | 0.518 | 0.001 | 122 |
| BCR(C) | WNT5A(C) | 0.805 | 0.409 | 0.519 | 0.865 | 123 |
| MSH2(N) | SGK2(C) | 0.432 | 0.539 | 0.535 | 0.104 | 121 |
| ABL1(C) | FEN1(N) | 0.897 | 0.212 | 0.536 | 0.923 | 123 |
| MSH2(C) | WNT5A(C) | 0.084 | 0.409 | 0.538 | 0.317 | 125 |
| ARK2(C) | TP53(N) | 0.968 | 0.351 | 0.538 | 0.159 | 123 |
| PARP1(N) | TP53(N) | 0.313 | 0.351 | 0.544 | 0.617 | 123 |
| RAD54B(N) | TP53(N) | 0.547 | 0.351 | 0.559 | 0.005 | 124 |
| CSNK1E(C) | MSH2(N) | 0.801 | 0.432 | 0.563 | 0.038 | 119 |
| ABL1(C) | RB1(N) | 0.897 | 0.220 | 0.564 | 0.970 | 122 |
| SKP2(C) | WNT5A(C) | 0.767 | 0.409 | 0.564 | 0.722 | 122 |
| FEN1(C) | SKP2(C) | 0.585 | 0.767 | 0.564 | 0.867 | 122 |
| MSH2(N) | TP53(N) | 0.432 | 0.351 | 0.566 | 0.608 | 126 |
| CDH1(M) | MSH2(N) | 0.472 | 0.432 | 0.576 | 0.057 | 123 |
| ABL1(C) | BRCA2(C) | 0.897 | 0.245 | 0.583 | 0.282 | 119 |
| BRCA1(C) | SKP2(C) | 0.355 | 0.767 | 0.585 | 0.890 | 118 |
| BCR(C) | BRCA1(C) | 0.805 | 0.355 | 0.585 | 0.957 | 121 |
| CSNK1E(C) | PARP1(N) | 0.801 | 0.313 | 0.586 | 0.763 | 117 |
| ARK2(C) | BRCA2(C) | 0.968 | 0.245 | 0.587 | 0.670 | 119 |
| CDH1(M) | CSNK1E(C) | 0.472 | 0.801 | 0.588 | 0.909 | 119 |
| CSNK1E(C) | FEN1(C) | 0.801 | 0.585 | 0.588 | 0.225 | 119 |
| PARP1(N) | RAD54B(N) | 0.313 | 0.547 | 0.599 | 0.000 | 122 |
| MSH2(N) | WNT5A(C) | 0.432 | 0.409 | 0.605 | 0.178 | 125 |
| BCR(C) | BRCA2(N) | 0.805 | 0.190 | 0.620 | 0.721 | 121 |
| ARK2(C) | RAD54B(N) | 0.968 | 0.547 | 0.622 | 0.002 | 121 |
| ARK2(C) | WNT5A(C) | 0.968 | 0.409 | 0.633 | 0.301 | 123 |
| FEN1(N) | SKP2(C) | 0.212 | 0.767 | 0.653 | 0.868 | 122 |
| PARP1(N) | SKP2(C) | 0.313 | 0.767 | 0.653 | 0.470 | 120 |
| MSH2(N) | RB1(N) | 0.432 | 0.220 | 0.653 | 0.170 | 122 |
| CSNK1E(C) | SGK2(C) | 0.801 | 0.539 | 0.654 | 0.672 | 119 |
| MSH2(C) | TP53(N) | 0.084 | 0.351 | 0.655 | 0.538 | 126 |
| BRCA2(C) | MYC(N) | 0.245 | 0.934 | 0.662 | 0.511 | 118 |
| CSNK1E(C) | WNT5A(C) | 0.801 | 0.409 | 0.665 | 0.844 | 119 |
| EGFR(C) | MSH2(N) | 0.103 | 0.432 | 0.678 | 0.349 | 122 |
| BCR(C) | FEN1(C) | 0.805 | 0.585 | 0.709 | 0.666 | 124 |
| ARK2(C) | BRCA1(C) | 0.968 | 0.355 | 0.720 | 0.307 | 120 |
| BRCA1(C) | CSNK1E(C) | 0.355 | 0.801 | 0.721 | 0.907 | 117 |
| SKP2(C) | TP53(N) | 0.767 | 0.351 | 0.727 | 0.551 | 122 |
| ARK2(C) | PARP1(N) | 0.968 | 0.313 | 0.730 | 0.246 | 121 |
| BRCA2(N) | CTNNB1(N) | 0.190 | 0.996 | 0.731 | 0.061 | 121 |
| ARK2(C) | MSH2(N) | 0.968 | 0.432 | 0.733 | 0.379 | 123 |
| MSH2(N) | RAD54B(N) | 0.432 | 0.547 | 0.736 | 0.086 | 126 |
| ARK2(C) | CDH1(M) | 0.968 | 0.472 | 0.738 | 0.697 | 122 |
| MYC(N) | TP53(N) | 0.934 | 0.351 | 0.747 | 0.636 | 121 |
| BRCA2(N) | EGFR(C) | 0.190 | 0.103 | 0.748 | 0.289 | 117 |
| CSNK1E(C) | POLB(N) | 0.801 | 0.314 | 0.753 | 0.698 | 119 |
| BRCA1(C) | MYC(N) | 0.355 | 0.934 | 0.766 | 0.402 | 119 |
| BCR(C) | RAD54B(N) | 0.805 | 0.547 | 0.767 | 0.047 | 122 |
| POLB(N) | SKP2(C) | 0.314 | 0.767 | 0.767 | 0.746 | 122 |
| BCR(C) | PARP1(N) | 0.805 | 0.313 | 0.771 | 0.763 | 122 |
| MSH2(N) | PARP1(N) | 0.432 | 0.313 | 0.772 | 0.693 | 123 |
| ARK2(C) | SKP2(C) | 0.968 | 0.767 | 0.776 | 0.717 | 121 |
| ARK2(C) | POLB(N) | 0.968 | 0.314 | 0.785 | 0.323 | 123 |
| MYC(N) | RB1(N) | 0.934 | 0.220 | 0.786 | 0.744 | 121 |
| CSNK1E(C) | RAD54B(N) | 0.801 | 0.547 | 0.787 | 0.001 | 117 |
| BCR(C) | POLB(N) | 0.805 | 0.314 | 0.788 | 0.717 | 123 |
| RB1(N) | SKP2(C) | 0.220 | 0.767 | 0.791 | 0.520 | 121 |
| ABL1(C) | BRCA1(C) | 0.897 | 0.355 | 0.800 | 0.149 | 120 |
| ABL1(C) | RAD54B(N) | 0.897 | 0.547 | 0.803 | 0.009 | 121 |
| SGK2(C) | SKP2(C) | 0.539 | 0.767 | 0.803 | 0.480 | 121 |
| ABL1(C) | SGK2(C) | 0.897 | 0.539 | 0.807 | 0.191 | 121 |
| MSH2(N) | POLB(N) | 0.432 | 0.314 | 0.823 | 0.329 | 124 |
| CDH1(M) | WNT5A(C) | 0.472 | 0.409 | 0.832 | 0.627 | 123 |
| ABL1(C) | PARP1(N) | 0.897 | 0.313 | 0.834 | 0.439 | 121 |
| BCR(C) | SKP2(C) | 0.805 | 0.767 | 0.835 | 0.161 | 121 |
| FEN1(C) | MYC(N) | 0.585 | 0.934 | 0.837 | 0.550 | 121 |
| BCR(C) | RB1(N) | 0.805 | 0.220 | 0.845 | 0.939 | 121 |
| FEN1(C) | MSH2(N) | 0.585 | 0.432 | 0.848 | 0.048 | 129 |
| BCR(C) | SGK2(C) | 0.805 | 0.539 | 0.850 | 0.811 | 120 |
| FEN1(N) | MYC(N) | 0.212 | 0.934 | 0.857 | 0.067 | 121 |
| ARK2(C) | BCR(C) | 0.968 | 0.805 | 0.859 | 0.935 | 122 |
| BCR(C) | CSNK1E(C) | 0.805 | 0.801 | 0.862 | 0.682 | 119 |
| ABL1(C) | MSH2(C) | 0.897 | 0.084 | 0.862 | 0.223 | 123 |
| ABL1(C) | MSH2(N) | 0.897 | 0.432 | 0.862 | 0.149 | 123 |
| BRCA2(N) | SKP2(C) | 0.190 | 0.767 | 0.865 | 0.275 | 118 |
| ABL1(C) | BRCA2(N) | 0.897 | 0.190 | 0.869 | 0.275 | 119 |
| ABL1(C) | EGFR(C) | 0.897 | 0.103 | 0.869 | 0.625 | 121 |
| ABL1(C) | CDH1(M) | 0.897 | 0.472 | 0.871 | 0.081 | 122 |
| ABL1(C) | MYC(N) | 0.897 | 0.934 | 0.879 | 0.678 | 121 |
| BCR(C) | BRCA2(C) | 0.805 | 0.245 | 0.879 | 0.760 | 121 |
| CSNK1E(C) | SKP2(C) | 0.801 | 0.767 | 0.881 | 0.852 | 119 |
| ABL1(C) | CSNK1E(C) | 0.897 | 0.801 | 0.887 | 0.270 | 119 |
| ABL1(C) | ARK2(C) | 0.897 | 0.968 | 0.888 | 0.313 | 122 |
| ABL1(C) | POLB(N) | 0.897 | 0.314 | 0.888 | 0.311 | 122 |
| ABL1(C) | FEN1(C) | 0.897 | 0.585 | 0.892 | 0.687 | 123 |
| ABL1(C) | WNT5A(C) | 0.897 | 0.409 | 0.898 | 0.630 | 122 |
| CDH1(M) | FEN1(C) | 0.472 | 0.585 | 0.899 | 0.319 | 123 |
| ARK2(C) | SGK2(C) | 0.968 | 0.539 | 0.899 | 0.239 | 121 |
| ABL1(C) | BCR(C) | 0.897 | 0.805 | 0.911 | 0.383 | 122 |
| MYC(N) | SGK2(C) | 0.934 | 0.539 | 0.924 | 0.369 | 120 |
| CDH1(M) | SKP2(C) | 0.472 | 0.767 | 0.926 | 0.801 | 122 |
| CDH1(M) | CTNNB1(N) | 0.472 | 0.996 | 0.928 | 0.334 | 122 |
| CTNNB1(N) | FEN1(C) | 0.996 | 0.585 | 0.930 | 0.858 | 125 |
| BRCA2(C) | SKP2(C) | 0.245 | 0.767 | 0.932 | 0.976 | 118 |
| MYC(N) | SKP2(C) | 0.934 | 0.767 | 0.932 | 0.544 | 120 |
| MYC(N) | RAD54B(N) | 0.934 | 0.547 | 0.933 | 0.378 | 119 |
| FEN1(N) | MSH2(N) | 0.212 | 0.432 | 0.935 | 0.309 | 129 |
| BCR(C) | MYC(N) | 0.805 | 0.934 | 0.936 | 0.704 | 121 |
| MYC(N) | POLB(N) | 0.934 | 0.314 | 0.938 | 0.375 | 121 |
| MYC(N) | PARP1(N) | 0.934 | 0.313 | 0.946 | 0.315 | 119 |
| CSNK1E(C) | MYC(N) | 0.801 | 0.934 | 0.954 | 0.161 | 119 |
| ARK2(C) | CSNK1E(C) | 0.968 | 0.801 | 0.955 | 0.908 | 119 |
| CDH1(M) | MYC(N) | 0.472 | 0.934 | 0.956 | 0.809 | 121 |
| ARK2(C) | MYC(N) | 0.968 | 0.934 | 0.956 | 0.289 | 121 |
| CTNNB1(N) | RB1(N) | 0.996 | 0.220 | 0.965 | 0.569 | 121 |
| MYC(N) | WNT5A(C) | 0.934 | 0.409 | 0.975 | 0.889 | 121 |
| ABL1(C) | SKP2(C) | 0.897 | 0.767 | 0.982 | 0.085 | 121 |
| CTNNB1(N) | PARP1(N) | 0.996 | 0.313 | 0.990 | 0.562 | 122 |
| CTNNB1(N) | POLB(N) | 0.996 | 0.314 | 0.990 | 0.545 | 123 |
| ARK2(C) | CTNNB1(N) | 0.968 | 0.996 | 0.991 | 0.559 | 122 |
| BCR(C) | CDH1(M) | 0.805 | 0.472 | 0.993 | 0.943 | 122 |
| CTNNB1(N) | SGK2(C) | 0.996 | 0.539 | 0.996 | 0.389 | 120 |
| CSNK1E(C) | CTNNB1(N) | 0.801 | 0.996 | 0.996 | 0.404 | 119 |
| ABL1(C) | CTNNB1(N) | 0.897 | 0.996 | 1.000 | 0.777 | 122 |
| BCR(C) | CTNNB1(N) | 0.805 | 0.996 | 1.000 | 0.214 | 124 |
| BRCA1(C) | CTNNB1(N) | 0.355 | 0.996 | 1.000 | 0.488 | 121 |
| BRCA2(C) | CTNNB1(N) | 0.245 | 0.996 | 1.000 | 0.516 | 121 |
| CTNNB1(N) | MYC(N) | 0.996 | 0.934 | 1.000 | 0.438 | 121 |
| CTNNB1(N) | EGFR(C) | 0.996 | 0.103 | 1.000 | 0.211 | 120 |
| CTNNB1(N) | MSH2(C) | 0.996 | 0.084 | 1.000 | 0.111 | 125 |
| CTNNB1(N) | MSH2(N) | 0.996 | 0.432 | 1.000 | 0.218 | 125 |
| CTNNB1(N) | TP53(N) | 0.996 | 0.351 | 1.000 | 0.481 | 124 |
| CTNNB1(N) | RAD54B(N) | 0.996 | 0.547 | 1.000 | 0.201 | 122 |
| CTNNB1(N) | SKP2(C) | 0.996 | 0.767 | 1.000 | 0.198 | 121 |
| CTNNB1(N) | WNT5A(C) | 0.996 | 0.409 | 1.000 | 0.247 | 123 |
| CTNNB1(N) | FEN1(N) | 0.996 | 0.212 | 1.000 | 0.456 | 125 |
| BCR(C) | MSH2(C) | 0.805 | 0.084 | 1.000 | 0.137 | 124 |
| BCR(C) | MSH2(N) | 0.805 | 0.432 | 1.000 | 0.414 | 124 |
| MSH2(N) | SKP2(C) | 0.432 | 0.767 | NA | 0.746 | 122 |

| Protein1 | Protein2 | *p*-value | | | Log Rank | Total Number |
| --- | --- | --- | --- | --- | --- | --- |
|  |  | Protein1 | Protein2 | Protein.Pairs |  |  |
| D. Grade | | | | | | |
| ABL1(C) | CTNNB1(N) | 0.781 | 0.169 | 0.021 | 0.777 | 123 |
| ARK2(C) | BRCA2(N) | 0.380 | 0.108 | 0.031 | 0.748 | 120 |
| BRCA2(N) | POLB(N) | 0.108 | 0.384 | 0.039 | 0.975 | 121 |
| MSH2(C) | MYC(N) | 0.058 | 0.448 | 0.047 | 0.590 | 122 |
| BRCA1(C) | BRCA2(N) | 0.407 | 0.108 | 0.049 | 0.398 | 120 |
| ARK2(C) | MSH2(C) | 0.380 | 0.058 | 0.052 | 0.654 | 124 |
| MSH2(C) | POLB(N) | 0.058 | 0.384 | 0.059 | 0.980 | 125 |
| BRCA1(C) | CTNNB1(N) | 0.407 | 0.169 | 0.064 | 0.488 | 122 |
| BRCA2(N) | MYC(N) | 0.108 | 0.448 | 0.067 | 0.408 | 119 |
| BRCA2(N) | MSH2(C) | 0.108 | 0.058 | 0.075 | 0.893 | 123 |
| RAD54B(N) | RB1(N) | 0.380 | 0.356 | 0.083 | 0.024 | 121 |
| MSH2(C) | WNT5A(C) | 0.058 | 0.681 | 0.089 | 0.317 | 126 |
| BCR(C) | BRCA2(N) | 0.870 | 0.108 | 0.091 | 0.721 | 122 |
| BRCA2(N) | CSNK1E(C) | 0.108 | 0.501 | 0.093 | 0.780 | 117 |
| MSH2(C) | RB1(N) | 0.058 | 0.356 | 0.093 | 0.581 | 123 |
| BRCA2(N) | PARP1(N) | 0.108 | 0.570 | 0.097 | 0.916 | 121 |
| MSH2(C) | SGK2(C) | 0.058 | 0.936 | 0.098 | 0.505 | 122 |
| BRCA2(N) | RB1(N) | 0.108 | 0.356 | 0.101 | 0.682 | 119 |
| BRCA1(C) | MSH2(C) | 0.407 | 0.058 | 0.101 | 0.422 | 124 |
| CSNK1E(C) | MSH2(C) | 0.501 | 0.058 | 0.104 | 0.320 | 120 |
| BRCA2(N) | SGK2(C) | 0.108 | 0.936 | 0.105 | 0.835 | 118 |
| CTNNB1(N) | POLB(N) | 0.169 | 0.384 | 0.118 | 0.545 | 124 |
| ARK2(C) | CTNNB1(N) | 0.380 | 0.169 | 0.119 | 0.559 | 123 |
| CTNNB1(N) | PARP1(N) | 0.169 | 0.570 | 0.119 | 0.562 | 123 |
| MSH2(C) | PARP1(N) | 0.058 | 0.570 | 0.121 | 0.960 | 124 |
| MSH2(C) | RAD54B(N) | 0.058 | 0.380 | 0.132 | 0.696 | 127 |
| CTNNB1(N) | SGK2(C) | 0.169 | 0.936 | 0.156 | 0.389 | 121 |
| CSNK1E(C) | CTNNB1(N) | 0.501 | 0.169 | 0.158 | 0.404 | 120 |
| BCR(C) | CTNNB1(N) | 0.870 | 0.169 | 0.169 | 0.214 | 125 |
| BCR(C) | MSH2(C) | 0.870 | 0.058 | 0.169 | 0.137 | 125 |
| ARK2(C) | CSNK1E(C) | 0.380 | 0.501 | 0.172 | 0.908 | 120 |
| BRCA2(N) | CTNNB1(N) | 0.108 | 0.169 | 0.173 | 0.061 | 122 |
| CTNNB1(N) | SKP2(C) | 0.169 | 0.693 | 0.173 | 0.198 | 122 |
| ARK2(C) | POLB(N) | 0.380 | 0.384 | 0.204 | 0.323 | 124 |
| ARK2(C) | BRCA1(C) | 0.380 | 0.407 | 0.228 | 0.307 | 121 |
| BRCA1(C) | RB1(N) | 0.407 | 0.356 | 0.237 | 0.767 | 120 |
| POLB(N) | RB1(N) | 0.384 | 0.356 | 0.237 | 0.435 | 123 |
| CSNK1E(C) | POLB(N) | 0.501 | 0.384 | 0.240 | 0.698 | 120 |
| BRCA2(C) | MSH2(C) | 0.697 | 0.058 | 0.241 | 0.860 | 123 |
| CTNNB1(N) | MYC(N) | 0.169 | 0.448 | 0.249 | 0.438 | 122 |
| BRCA2(N) | RAD54B(N) | 0.108 | 0.380 | 0.249 | 0.266 | 122 |
| ABL1(C) | BRCA2(N) | 0.781 | 0.108 | 0.252 | 0.275 | 120 |
| CSNK1E(C) | RAD54B(N) | 0.501 | 0.380 | 0.253 | 0.001 | 118 |
| BRCA2(N) | SKP2(C) | 0.108 | 0.693 | 0.254 | 0.275 | 119 |
| ARK2(C) | RB1(N) | 0.380 | 0.356 | 0.255 | 0.479 | 123 |
| CTNNB1(N) | MSH2(C) | 0.169 | 0.058 | 0.261 | 0.111 | 126 |
| FEN1(N) | RB1(N) | 0.891 | 0.356 | 0.263 | 0.152 | 123 |
| FEN1(N) | RAD54B(N) | 0.891 | 0.380 | 0.279 | 0.000 | 127 |
| PARP1(N) | RAD54B(N) | 0.570 | 0.380 | 0.282 | 0.000 | 123 |
| CSNK1E(C) | RB1(N) | 0.501 | 0.356 | 0.284 | 0.732 | 120 |
| BRCA1(C) | POLB(N) | 0.407 | 0.384 | 0.287 | 0.163 | 121 |
| ARK2(C) | RAD54B(N) | 0.380 | 0.380 | 0.289 | 0.002 | 122 |
| CDH1(M) | RAD54B(N) | 0.933 | 0.380 | 0.292 | 0.002 | 122 |
| FEN1(N) | MSH2(C) | 0.891 | 0.058 | 0.302 | 0.347 | 130 |
| POLB(N) | RAD54B(N) | 0.384 | 0.380 | 0.303 | 0.001 | 123 |
| FEN1(C) | RAD54B(N) | 0.914 | 0.380 | 0.304 | 0.048 | 127 |
| BRCA2(C) | MYC(N) | 0.697 | 0.448 | 0.311 | 0.511 | 119 |
| ABL1(C) | MSH2(C) | 0.781 | 0.058 | 0.314 | 0.223 | 124 |
| CTNNB1(N) | WNT5A(C) | 0.169 | 0.681 | 0.314 | 0.247 | 124 |
| FEN1(C) | MSH2(C) | 0.914 | 0.058 | 0.343 | 0.069 | 130 |
| RB1(N) | SGK2(C) | 0.356 | 0.936 | 0.345 | 0.633 | 122 |
| BRCA1(C) | CSNK1E(C) | 0.407 | 0.501 | 0.357 | 0.907 | 118 |
| MYC(N) | WNT5A(C) | 0.448 | 0.681 | 0.365 | 0.889 | 122 |
| BRCA2(C) | RAD54B(N) | 0.697 | 0.380 | 0.371 | 0.008 | 122 |
| PARP1(N) | RB1(N) | 0.570 | 0.356 | 0.373 | 0.140 | 121 |
| MYC(N) | POLB(N) | 0.448 | 0.384 | 0.378 | 0.375 | 122 |
| CSNK1E(C) | PARP1(N) | 0.501 | 0.570 | 0.380 | 0.763 | 118 |
| BRCA1(C) | PARP1(N) | 0.407 | 0.570 | 0.381 | 0.163 | 121 |
| BRCA2(C) | CTNNB1(N) | 0.697 | 0.169 | 0.382 | 0.516 | 122 |
| MYC(N) | SGK2(C) | 0.448 | 0.936 | 0.387 | 0.369 | 121 |
| MYC(N) | PARP1(N) | 0.448 | 0.570 | 0.395 | 0.315 | 120 |
| RAD54B(N) | WNT5A(C) | 0.380 | 0.681 | 0.400 | 0.007 | 124 |
| BRCA1(C) | MSH2(N) | 0.407 | 0.469 | 0.404 | 0.080 | 124 |
| BRCA1(C) | MYC(N) | 0.407 | 0.448 | 0.405 | 0.402 | 120 |
| MYC(N) | RAD54B(N) | 0.448 | 0.380 | 0.413 | 0.378 | 120 |
| BRCA1(C) | RAD54B(N) | 0.407 | 0.380 | 0.415 | 0.000 | 122 |
| CSNK1E(C) | MYC(N) | 0.501 | 0.448 | 0.419 | 0.161 | 120 |
| ARK2(C) | MYC(N) | 0.380 | 0.448 | 0.425 | 0.289 | 122 |
| BRCA1(C) | WNT5A(C) | 0.407 | 0.681 | 0.426 | 0.292 | 122 |
| ARK2(C) | PARP1(N) | 0.380 | 0.570 | 0.431 | 0.246 | 122 |
| BRCA2(N) | FEN1(N) | 0.108 | 0.891 | 0.437 | 0.683 | 123 |
| ARK2(C) | BRCA2(C) | 0.380 | 0.697 | 0.438 | 0.670 | 120 |
| BCR(C) | BRCA2(C) | 0.870 | 0.697 | 0.439 | 0.760 | 122 |
| ABL1(C) | SKP2(C) | 0.781 | 0.693 | 0.440 | 0.085 | 122 |
| MSH2(N) | WNT5A(C) | 0.469 | 0.681 | 0.459 | 0.178 | 126 |
| PARP1(N) | POLB(N) | 0.570 | 0.384 | 0.467 | 0.318 | 123 |
| BRCA2(C) | POLB(N) | 0.697 | 0.384 | 0.471 | 0.643 | 121 |
| BRCA2(C) | RB1(N) | 0.697 | 0.356 | 0.477 | 0.538 | 119 |
| FEN1(C) | RB1(N) | 0.914 | 0.356 | 0.483 | 0.658 | 123 |
| BRCA2(N) | TP53(N) | 0.108 | 0.867 | 0.493 | 0.480 | 122 |
| BRCA2(C) | SKP2(C) | 0.697 | 0.693 | 0.502 | 0.976 | 119 |
| RAD54B(N) | TP53(N) | 0.380 | 0.867 | 0.503 | 0.005 | 125 |
| RAD54B(N) | SGK2(C) | 0.380 | 0.936 | 0.507 | 0.001 | 120 |
| MYC(N) | RB1(N) | 0.448 | 0.356 | 0.538 | 0.744 | 122 |
| BRCA2(N) | FEN1(C) | 0.108 | 0.914 | 0.538 | 0.935 | 123 |
| BCR(C) | SKP2(C) | 0.870 | 0.693 | 0.541 | 0.161 | 122 |
| BRCA2(N) | WNT5A(C) | 0.108 | 0.681 | 0.545 | 0.588 | 121 |
| RB1(N) | TP53(N) | 0.356 | 0.867 | 0.558 | 0.173 | 123 |
| ABL1(C) | BRCA1(C) | 0.781 | 0.407 | 0.575 | 0.149 | 121 |
| BRCA2(N) | MSH2(N) | 0.108 | 0.469 | 0.582 | 0.547 | 123 |
| CDH1(M) | TP53(N) | 0.933 | 0.867 | 0.594 | 0.720 | 124 |
| ARK2(C) | SGK2(C) | 0.380 | 0.936 | 0.596 | 0.239 | 122 |
| BRCA2(C) | PARP1(N) | 0.697 | 0.570 | 0.604 | 0.721 | 121 |
| RB1(N) | WNT5A(C) | 0.356 | 0.681 | 0.609 | 0.351 | 123 |
| BRCA2(C) | CSNK1E(C) | 0.697 | 0.501 | 0.626 | 0.218 | 117 |
| SGK2(C) | SKP2(C) | 0.936 | 0.693 | 0.626 | 0.480 | 122 |
| POLB(N) | SGK2(C) | 0.384 | 0.936 | 0.632 | 0.156 | 122 |
| BRCA1(C) | SKP2(C) | 0.407 | 0.693 | 0.636 | 0.890 | 119 |
| BRCA1(C) | SGK2(C) | 0.407 | 0.936 | 0.639 | 0.405 | 119 |
| MSH2(N) | TP53(N) | 0.469 | 0.867 | 0.644 | 0.608 | 127 |
| TP53(N) | WNT5A(C) | 0.867 | 0.681 | 0.661 | 0.128 | 126 |
| CSNK1E(C) | WNT5A(C) | 0.501 | 0.681 | 0.661 | 0.844 | 120 |
| EGFR(C) | RB1(N) | 0.927 | 0.356 | 0.666 | 0.588 | 121 |
| PARP1(N) | SKP2(C) | 0.570 | 0.693 | 0.666 | 0.470 | 121 |
| CSNK1E(C) | SKP2(C) | 0.501 | 0.693 | 0.669 | 0.852 | 120 |
| BRCA2(C) | WNT5A(C) | 0.697 | 0.681 | 0.669 | 0.989 | 121 |
| BRCA2(C) | SGK2(C) | 0.697 | 0.936 | 0.681 | 0.317 | 118 |
| PARP1(N) | WNT5A(C) | 0.570 | 0.681 | 0.689 | 0.720 | 123 |
| POLB(N) | SKP2(C) | 0.384 | 0.693 | 0.693 | 0.746 | 123 |
| ARK2(C) | SKP2(C) | 0.380 | 0.693 | 0.696 | 0.717 | 122 |
| ARK2(C) | FEN1(N) | 0.380 | 0.891 | 0.697 | 0.809 | 124 |
| CSNK1E(C) | SGK2(C) | 0.501 | 0.936 | 0.698 | 0.672 | 120 |
| CDH1(M) | WNT5A(C) | 0.933 | 0.681 | 0.702 | 0.627 | 124 |
| BCR(C) | BRCA1(C) | 0.870 | 0.407 | 0.705 | 0.957 | 122 |
| MSH2(N) | POLB(N) | 0.469 | 0.384 | 0.717 | 0.329 | 125 |
| BRCA2(C) | FEN1(C) | 0.697 | 0.914 | 0.721 | 0.072 | 123 |
| MSH2(N) | RB1(N) | 0.469 | 0.356 | 0.723 | 0.170 | 123 |
| POLB(N) | WNT5A(C) | 0.384 | 0.681 | 0.725 | 0.545 | 125 |
| BCR(C) | RB1(N) | 0.870 | 0.356 | 0.727 | 0.939 | 122 |
| MYC(N) | TP53(N) | 0.448 | 0.867 | 0.728 | 0.636 | 122 |
| PARP1(N) | SGK2(C) | 0.570 | 0.936 | 0.732 | 0.236 | 120 |
| ABL1(C) | BRCA2(C) | 0.781 | 0.697 | 0.733 | 0.282 | 120 |
| ARK2(C) | TP53(N) | 0.380 | 0.867 | 0.735 | 0.159 | 124 |
| CSNK1E(C) | TP53(N) | 0.501 | 0.867 | 0.736 | 0.628 | 120 |
| CSNK1E(C) | FEN1(N) | 0.501 | 0.891 | 0.736 | 0.597 | 120 |
| ARK2(C) | WNT5A(C) | 0.380 | 0.681 | 0.737 | 0.301 | 124 |
| FEN1(C) | PARP1(N) | 0.914 | 0.570 | 0.737 | 0.475 | 124 |
| ABL1(C) | CSNK1E(C) | 0.781 | 0.501 | 0.738 | 0.270 | 120 |
| POLB(N) | TP53(N) | 0.384 | 0.867 | 0.744 | 0.204 | 125 |
| FEN1(N) | POLB(N) | 0.891 | 0.384 | 0.744 | 0.458 | 125 |
| BRCA1(C) | TP53(N) | 0.407 | 0.867 | 0.747 | 0.097 | 123 |
| BRCA2(C) | TP53(N) | 0.697 | 0.867 | 0.747 | 0.761 | 122 |
| ARK2(C) | MSH2(N) | 0.380 | 0.469 | 0.748 | 0.379 | 124 |
| ABL1(C) | SGK2(C) | 0.781 | 0.936 | 0.750 | 0.191 | 122 |
| FEN1(N) | PARP1(N) | 0.891 | 0.570 | 0.754 | 0.869 | 124 |
| CDH1(M) | RB1(N) | 0.933 | 0.356 | 0.760 | 0.479 | 123 |
| CSNK1E(C) | FEN1(C) | 0.501 | 0.914 | 0.763 | 0.225 | 120 |
| ABL1(C) | POLB(N) | 0.781 | 0.384 | 0.766 | 0.311 | 123 |
| MSH2(C) | TP53(N) | 0.058 | 0.867 | 0.770 | 0.538 | 127 |
| ABL1(C) | PARP1(N) | 0.781 | 0.570 | 0.770 | 0.439 | 122 |
| CDH1(M) | MYC(N) | 0.933 | 0.448 | 0.770 | 0.809 | 122 |
| CDH1(M) | MSH2(C) | 0.933 | 0.058 | 0.773 | 0.525 | 124 |
| CDH1(M) | FEN1(C) | 0.933 | 0.914 | 0.776 | 0.319 | 124 |
| FEN1(C) | POLB(N) | 0.914 | 0.384 | 0.781 | 0.260 | 125 |
| ABL1(C) | ARK2(C) | 0.781 | 0.380 | 0.785 | 0.313 | 123 |
| BRCA2(C) | MSH2(N) | 0.697 | 0.469 | 0.799 | 0.114 | 123 |
| BRCA1(C) | FEN1(N) | 0.407 | 0.891 | 0.801 | 0.056 | 123 |
| MSH2(N) | SGK2(C) | 0.469 | 0.936 | 0.802 | 0.104 | 122 |
| BRCA1(C) | BRCA2(C) | 0.407 | 0.697 | 0.807 | 0.343 | 120 |
| CSNK1E(C) | MSH2(N) | 0.501 | 0.469 | 0.808 | 0.038 | 120 |
| ABL1(C) | MYC(N) | 0.781 | 0.448 | 0.822 | 0.678 | 122 |
| ARK2(C) | FEN1(C) | 0.380 | 0.914 | 0.824 | 0.203 | 124 |
| FEN1(C) | MYC(N) | 0.914 | 0.448 | 0.824 | 0.550 | 122 |
| EGFR(C) | FEN1(N) | 0.927 | 0.891 | 0.828 | 0.720 | 123 |
| BCR(C) | SGK2(C) | 0.870 | 0.936 | 0.829 | 0.811 | 121 |
| BCR(C) | CSNK1E(C) | 0.870 | 0.501 | 0.833 | 0.682 | 120 |
| CDH1(M) | FEN1(N) | 0.933 | 0.891 | 0.836 | 0.184 | 124 |
| FEN1(C) | TP53(N) | 0.914 | 0.867 | 0.842 | 0.607 | 127 |
| FEN1(N) | WNT5A(C) | 0.891 | 0.681 | 0.847 | 0.986 | 126 |
| BRCA1(C) | FEN1(C) | 0.407 | 0.914 | 0.847 | 0.771 | 123 |
| ABL1(C) | BCR(C) | 0.781 | 0.870 | 0.855 | 0.383 | 123 |
| ARK2(C) | BCR(C) | 0.380 | 0.870 | 0.864 | 0.935 | 123 |
| BCR(C) | MYC(N) | 0.870 | 0.448 | 0.873 | 0.704 | 122 |
| BCR(C) | POLB(N) | 0.870 | 0.384 | 0.873 | 0.717 | 124 |
| CDH1(M) | PARP1(N) | 0.933 | 0.570 | 0.876 | 0.766 | 122 |
| SGK2(C) | WNT5A(C) | 0.936 | 0.681 | 0.876 | 0.333 | 122 |
| BCR(C) | PARP1(N) | 0.870 | 0.570 | 0.877 | 0.763 | 123 |
| EGFR(C) | FEN1(C) | 0.927 | 0.914 | 0.880 | 0.514 | 123 |
| FEN1(C) | WNT5A(C) | 0.914 | 0.681 | 0.881 | 0.799 | 126 |
| BRCA1(C) | EGFR(C) | 0.407 | 0.927 | 0.887 | 0.976 | 120 |
| PARP1(N) | TP53(N) | 0.570 | 0.867 | 0.887 | 0.617 | 124 |
| EGFR(C) | SGK2(C) | 0.927 | 0.936 | 0.889 | 0.805 | 121 |
| ARK2(C) | CDH1(M) | 0.380 | 0.933 | 0.892 | 0.697 | 123 |
| FEN1(N) | SGK2(C) | 0.891 | 0.936 | 0.898 | 0.558 | 122 |
| EGFR(C) | POLB(N) | 0.927 | 0.384 | 0.901 | 0.564 | 121 |
| BRCA2(C) | CDH1(M) | 0.697 | 0.933 | 0.903 | 0.191 | 120 |
| CSNK1E(C) | EGFR(C) | 0.501 | 0.927 | 0.903 | 0.648 | 120 |
| CDH1(M) | POLB(N) | 0.933 | 0.384 | 0.907 | 0.602 | 124 |
| FEN1(C) | SGK2(C) | 0.914 | 0.936 | 0.909 | 0.426 | 122 |
| CDH1(M) | CSNK1E(C) | 0.933 | 0.501 | 0.911 | 0.909 | 120 |
| ARK2(C) | EGFR(C) | 0.380 | 0.927 | 0.912 | 0.602 | 121 |
| EGFR(C) | PARP1(N) | 0.927 | 0.570 | 0.914 | 0.637 | 120 |
| BRCA2(C) | FEN1(N) | 0.697 | 0.891 | 0.918 | 0.782 | 123 |
| SGK2(C) | TP53(N) | 0.936 | 0.867 | 0.925 | 0.494 | 122 |
| ABL1(C) | WNT5A(C) | 0.781 | 0.681 | 0.935 | 0.630 | 123 |
| BRCA1(C) | CDH1(M) | 0.407 | 0.933 | 0.937 | 0.341 | 120 |
| FEN1(N) | MYC(N) | 0.891 | 0.448 | 0.944 | 0.067 | 122 |
| FEN1(N) | TP53(N) | 0.891 | 0.867 | 0.962 | 0.638 | 127 |
| CDH1(M) | SGK2(C) | 0.933 | 0.936 | 0.982 | 0.895 | 122 |
| ABL1(C) | CDH1(M) | 0.781 | 0.933 | 1.000 | 0.081 | 123 |
| ABL1(C) | EGFR(C) | 0.781 | 0.927 | 1.000 | 0.625 | 122 |
| ABL1(C) | MSH2(N) | 0.781 | 0.469 | 1.000 | 0.149 | 124 |
| ABL1(C) | TP53(N) | 0.781 | 0.867 | 1.000 | 0.449 | 124 |
| ABL1(C) | RAD54B(N) | 0.781 | 0.380 | 1.000 | 0.009 | 122 |
| ABL1(C) | RB1(N) | 0.781 | 0.356 | 1.000 | 0.970 | 123 |
| ABL1(C) | FEN1(C) | 0.781 | 0.914 | 1.000 | 0.687 | 124 |
| ABL1(C) | FEN1(N) | 0.781 | 0.891 | 1.000 | 0.923 | 124 |
| CDH1(M) | CTNNB1(N) | 0.933 | 0.169 | 1.000 | 0.334 | 123 |
| CTNNB1(N) | EGFR(C) | 0.169 | 0.927 | 1.000 | 0.211 | 121 |
| CTNNB1(N) | MSH2(N) | 0.169 | 0.469 | 1.000 | 0.218 | 126 |
| CTNNB1(N) | TP53(N) | 0.169 | 0.867 | 1.000 | 0.481 | 125 |
| CTNNB1(N) | RAD54B(N) | 0.169 | 0.380 | 1.000 | 0.201 | 123 |
| CTNNB1(N) | RB1(N) | 0.169 | 0.356 | 1.000 | 0.569 | 122 |
| CTNNB1(N) | FEN1(C) | 0.169 | 0.914 | 1.000 | 0.858 | 126 |
| CTNNB1(N) | FEN1(N) | 0.169 | 0.891 | 1.000 | 0.456 | 126 |
| BCR(C) | CDH1(M) | 0.870 | 0.933 | 1.000 | 0.943 | 123 |
| BCR(C) | EGFR(C) | 0.870 | 0.927 | 1.000 | 0.476 | 121 |
| BCR(C) | MSH2(N) | 0.870 | 0.469 | 1.000 | 0.414 | 125 |
| BCR(C) | TP53(N) | 0.870 | 0.867 | 1.000 | 0.920 | 125 |
| BCR(C) | RAD54B(N) | 0.870 | 0.380 | 1.000 | 0.047 | 123 |
| BCR(C) | WNT5A(C) | 0.870 | 0.681 | 1.000 | 0.865 | 124 |
| BCR(C) | FEN1(C) | 0.870 | 0.914 | 1.000 | 0.666 | 125 |
| BCR(C) | FEN1(N) | 0.870 | 0.891 | 1.000 | 0.057 | 125 |
| BRCA2(C) | EGFR(C) | 0.697 | 0.927 | 1.000 | 0.619 | 118 |
| BRCA2(N) | CDH1(M) | 0.108 | 0.933 | 1.000 | 0.302 | 120 |
| BRCA2(N) | EGFR(C) | 0.108 | 0.927 | 1.000 | 0.289 | 118 |
| EGFR(C) | MYC(N) | 0.927 | 0.448 | 1.000 | 0.315 | 120 |
| MSH2(N) | MYC(N) | 0.469 | 0.448 | 1.000 | 0.057 | 122 |
| MYC(N) | SKP2(C) | 0.448 | 0.693 | 1.000 | 0.544 | 121 |
| CDH1(M) | EGFR(C) | 0.933 | 0.927 | 1.000 | 0.443 | 121 |
| CDH1(M) | MSH2(N) | 0.933 | 0.469 | 1.000 | 0.057 | 124 |
| CDH1(M) | SKP2(C) | 0.933 | 0.693 | 1.000 | 0.801 | 123 |
| EGFR(C) | MSH2(C) | 0.927 | 0.058 | 1.000 | 0.547 | 123 |
| EGFR(C) | MSH2(N) | 0.927 | 0.469 | 1.000 | 0.349 | 123 |
| EGFR(C) | TP53(N) | 0.927 | 0.867 | 1.000 | 0.927 | 123 |
| EGFR(C) | RAD54B(N) | 0.927 | 0.380 | 1.000 | 0.408 | 121 |
| EGFR(C) | SKP2(C) | 0.927 | 0.693 | 1.000 | 0.142 | 121 |
| EGFR(C) | WNT5A(C) | 0.927 | 0.681 | 1.000 | 0.277 | 122 |
| MSH2(C) | SKP2(C) | 0.058 | 0.693 | 1.000 | 0.412 | 123 |
| MSH2(N) | PARP1(N) | 0.469 | 0.570 | 1.000 | 0.693 | 124 |
| MSH2(N) | RAD54B(N) | 0.469 | 0.380 | 1.000 | 0.086 | 127 |
| FEN1(C) | MSH2(N) | 0.914 | 0.469 | 1.000 | 0.048 | 130 |
| FEN1(N) | MSH2(N) | 0.891 | 0.469 | 1.000 | 0.309 | 130 |
| SKP2(C) | TP53(N) | 0.693 | 0.867 | 1.000 | 0.551 | 123 |
| RAD54B(N) | SKP2(C) | 0.380 | 0.693 | 1.000 | 0.300 | 121 |
| RB1(N) | SKP2(C) | 0.356 | 0.693 | 1.000 | 0.520 | 122 |
| SKP2(C) | WNT5A(C) | 0.693 | 0.681 | 1.000 | 0.722 | 123 |
| FEN1(C) | SKP2(C) | 0.914 | 0.693 | 1.000 | 0.867 | 123 |
| FEN1(N) | SKP2(C) | 0.891 | 0.693 | 1.000 | 0.868 | 123 |
| MSH2(N) | SKP2(C) | 0.469 | 0.693 | NA | 0.746 | 123 |

| Protein1 | Protein2 | *p*-value | | | Log Rank | Total Number |
| --- | --- | --- | --- | --- | --- | --- |
|  |  | Protein1 | Protein2 | Protein.Pairs |  |  |
| E. Stage | | | | | | |
| CDH1(M) | RB1(N) | 0.346 | 0.107 | 0.012 | 0.479 | 122 |
| EGFR(C) | RB1(N) | 0.268 | 0.107 | 0.031 | 0.588 | 120 |
| FEN1(N) | MSH2(C) | 0.538 | 0.395 | 0.033 | 0.347 | 129 |
| BRCA1(C) | TP53(N) | 0.217 | 0.144 | 0.038 | 0.097 | 122 |
| PARP1(N) | RB1(N) | 0.381 | 0.107 | 0.048 | 0.140 | 120 |
| POLB(N) | RB1(N) | 0.230 | 0.107 | 0.051 | 0.435 | 122 |
| CDH1(M) | MSH2(C) | 0.346 | 0.395 | 0.059 | 0.525 | 123 |
| MSH2(C) | RAD54B(N) | 0.395 | 0.267 | 0.062 | 0.696 | 126 |
| RB1(N) | SGK2(C) | 0.107 | 0.476 | 0.063 | 0.633 | 121 |
| RB1(N) | TP53(N) | 0.107 | 0.144 | 0.073 | 0.173 | 122 |
| BRCA2(C) | MSH2(C) | 0.374 | 0.395 | 0.079 | 0.860 | 122 |
| BRCA2(C) | RB1(N) | 0.374 | 0.107 | 0.083 | 0.538 | 118 |
| BRCA1(C) | RAD54B(N) | 0.217 | 0.267 | 0.087 | 0.000 | 121 |
| BRCA1(C) | RB1(N) | 0.217 | 0.107 | 0.096 | 0.767 | 119 |
| MSH2(C) | RB1(N) | 0.395 | 0.107 | 0.101 | 0.581 | 122 |
| POLB(N) | TP53(N) | 0.230 | 0.144 | 0.105 | 0.204 | 124 |
| BRCA1(C) | POLB(N) | 0.217 | 0.230 | 0.108 | 0.163 | 120 |
| BRCA1(C) | PARP1(N) | 0.217 | 0.381 | 0.110 | 0.163 | 120 |
| BRCA2(N) | FEN1(N) | 0.785 | 0.538 | 0.111 | 0.683 | 122 |
| CSNK1E(C) | TP53(N) | 0.759 | 0.144 | 0.116 | 0.628 | 119 |
| BRCA2(C) | TP53(N) | 0.374 | 0.144 | 0.117 | 0.761 | 121 |
| SGK2(C) | TP53(N) | 0.476 | 0.144 | 0.121 | 0.494 | 121 |
| MSH2(C) | PARP1(N) | 0.395 | 0.381 | 0.128 | 0.960 | 123 |
| ARK2(C) | RB1(N) | 0.818 | 0.107 | 0.138 | 0.479 | 122 |
| RAD54B(N) | RB1(N) | 0.267 | 0.107 | 0.144 | 0.024 | 120 |
| BRCA1(C) | SGK2(C) | 0.217 | 0.476 | 0.147 | 0.405 | 118 |
| CDH1(M) | POLB(N) | 0.346 | 0.230 | 0.169 | 0.602 | 123 |
| FEN1(C) | RAD54B(N) | 0.558 | 0.267 | 0.172 | 0.048 | 126 |
| EGFR(C) | FEN1(C) | 0.268 | 0.558 | 0.179 | 0.514 | 122 |
| BRCA2(C) | CDH1(M) | 0.374 | 0.346 | 0.187 | 0.191 | 119 |
| BRCA1(C) | WNT5A(C) | 0.217 | 0.471 | 0.195 | 0.292 | 121 |
| CSNK1E(C) | EGFR(C) | 0.759 | 0.268 | 0.202 | 0.648 | 119 |
| FEN1(C) | MSH2(C) | 0.558 | 0.395 | 0.205 | 0.069 | 129 |
| CSNK1E(C) | RB1(N) | 0.759 | 0.107 | 0.207 | 0.732 | 119 |
| BRCA1(C) | FEN1(C) | 0.217 | 0.558 | 0.218 | 0.771 | 122 |
| CDH1(M) | SGK2(C) | 0.346 | 0.476 | 0.219 | 0.895 | 121 |
| MSH2(C) | POLB(N) | 0.395 | 0.230 | 0.219 | 0.980 | 124 |
| RAD54B(N) | TP53(N) | 0.267 | 0.144 | 0.219 | 0.005 | 124 |
| PARP1(N) | POLB(N) | 0.381 | 0.230 | 0.222 | 0.318 | 122 |
| ARK2(C) | MSH2(C) | 0.818 | 0.395 | 0.224 | 0.654 | 123 |
| CDH1(M) | TP53(N) | 0.346 | 0.144 | 0.230 | 0.720 | 123 |
| EGFR(C) | FEN1(N) | 0.268 | 0.538 | 0.232 | 0.720 | 122 |
| BRCA2(C) | PARP1(N) | 0.374 | 0.381 | 0.236 | 0.721 | 120 |
| BRCA2(C) | POLB(N) | 0.374 | 0.230 | 0.239 | 0.643 | 120 |
| BRCA2(N) | TP53(N) | 0.785 | 0.144 | 0.241 | 0.480 | 121 |
| MSH2(C) | SGK2(C) | 0.395 | 0.476 | 0.243 | 0.505 | 121 |
| CSNK1E(C) | MSH2(C) | 0.759 | 0.395 | 0.243 | 0.320 | 119 |
| BRCA1(C) | CDH1(M) | 0.217 | 0.346 | 0.243 | 0.341 | 119 |
| BRCA2(C) | WNT5A(C) | 0.374 | 0.471 | 0.247 | 0.989 | 120 |
| POLB(N) | SGK2(C) | 0.230 | 0.476 | 0.248 | 0.156 | 121 |
| EGFR(C) | PARP1(N) | 0.268 | 0.381 | 0.254 | 0.637 | 119 |
| MSH2(N) | RAD54B(N) | 0.923 | 0.267 | 0.256 | 0.086 | 126 |
| BRCA1(C) | EGFR(C) | 0.217 | 0.268 | 0.258 | 0.976 | 119 |
| ARK2(C) | EGFR(C) | 0.818 | 0.268 | 0.268 | 0.602 | 120 |
| EGFR(C) | POLB(N) | 0.268 | 0.230 | 0.268 | 0.564 | 120 |
| BRCA1(C) | FEN1(N) | 0.217 | 0.538 | 0.268 | 0.056 | 122 |
| RB1(N) | WNT5A(C) | 0.107 | 0.471 | 0.276 | 0.351 | 122 |
| RAD54B(N) | SGK2(C) | 0.267 | 0.476 | 0.278 | 0.001 | 119 |
| ABL1(C) | TP53(N) | 0.813 | 0.144 | 0.282 | 0.449 | 123 |
| BRCA2(C) | RAD54B(N) | 0.374 | 0.267 | 0.292 | 0.008 | 121 |
| FEN1(C) | RB1(N) | 0.558 | 0.107 | 0.299 | 0.658 | 122 |
| MSH2(C) | TP53(N) | 0.395 | 0.144 | 0.301 | 0.538 | 126 |
| TP53(N) | WNT5A(C) | 0.144 | 0.471 | 0.310 | 0.128 | 125 |
| BRCA2(C) | FEN1(C) | 0.374 | 0.558 | 0.321 | 0.072 | 122 |
| PARP1(N) | TP53(N) | 0.381 | 0.144 | 0.325 | 0.617 | 123 |
| ARK2(C) | BRCA1(C) | 0.818 | 0.217 | 0.326 | 0.307 | 120 |
| POLB(N) | RAD54B(N) | 0.230 | 0.267 | 0.330 | 0.001 | 122 |
| FEN1(N) | RB1(N) | 0.538 | 0.107 | 0.336 | 0.152 | 122 |
| BCR(C) | TP53(N) | 0.847 | 0.144 | 0.339 | 0.920 | 124 |
| FEN1(C) | POLB(N) | 0.558 | 0.230 | 0.372 | 0.260 | 124 |
| BCR(C) | EGFR(C) | 0.847 | 0.268 | 0.372 | 0.476 | 120 |
| BRCA2(N) | WNT5A(C) | 0.785 | 0.471 | 0.377 | 0.588 | 120 |
| PARP1(N) | SGK2(C) | 0.381 | 0.476 | 0.387 | 0.236 | 119 |
| BRCA2(C) | FEN1(N) | 0.374 | 0.538 | 0.396 | 0.782 | 122 |
| FEN1(C) | PARP1(N) | 0.558 | 0.381 | 0.398 | 0.475 | 123 |
| PARP1(N) | RAD54B(N) | 0.381 | 0.267 | 0.400 | 0.000 | 122 |
| FEN1(C) | TP53(N) | 0.558 | 0.144 | 0.402 | 0.607 | 126 |
| EGFR(C) | TP53(N) | 0.268 | 0.144 | 0.405 | 0.927 | 122 |
| SGK2(C) | WNT5A(C) | 0.476 | 0.471 | 0.408 | 0.333 | 121 |
| CDH1(M) | CSNK1E(C) | 0.346 | 0.759 | 0.412 | 0.909 | 119 |
| BRCA2(C) | SGK2(C) | 0.374 | 0.476 | 0.419 | 0.317 | 117 |
| ARK2(C) | RAD54B(N) | 0.818 | 0.267 | 0.425 | 0.002 | 121 |
| FEN1(N) | POLB(N) | 0.538 | 0.230 | 0.430 | 0.458 | 124 |
| BRCA1(C) | CSNK1E(C) | 0.217 | 0.759 | 0.431 | 0.907 | 117 |
| EGFR(C) | SGK2(C) | 0.268 | 0.476 | 0.436 | 0.805 | 120 |
| POLB(N) | WNT5A(C) | 0.230 | 0.471 | 0.440 | 0.545 | 124 |
| BRCA2(C) | CSNK1E(C) | 0.374 | 0.759 | 0.443 | 0.218 | 116 |
| CSNK1E(C) | POLB(N) | 0.759 | 0.230 | 0.461 | 0.698 | 119 |
| ARK2(C) | TP53(N) | 0.818 | 0.144 | 0.463 | 0.159 | 123 |
| CDH1(M) | PARP1(N) | 0.346 | 0.381 | 0.469 | 0.766 | 121 |
| ARK2(C) | BRCA2(C) | 0.818 | 0.374 | 0.477 | 0.670 | 119 |
| CSNK1E(C) | WNT5A(C) | 0.759 | 0.471 | 0.477 | 0.844 | 119 |
| CSNK1E(C) | PARP1(N) | 0.759 | 0.381 | 0.483 | 0.763 | 117 |
| FEN1(C) | SGK2(C) | 0.558 | 0.476 | 0.486 | 0.426 | 121 |
| CSNK1E(C) | RAD54B(N) | 0.759 | 0.267 | 0.498 | 0.001 | 117 |
| MSH2(C) | SKP2(C) | 0.395 | 0.834 | 0.508 | 0.412 | 122 |
| EGFR(C) | MSH2(C) | 0.268 | 0.395 | 0.512 | 0.547 | 122 |
| EGFR(C) | SKP2(C) | 0.268 | 0.834 | 0.513 | 0.142 | 120 |
| EGFR(C) | WNT5A(C) | 0.268 | 0.471 | 0.514 | 0.277 | 121 |
| CDH1(M) | MYC(N) | 0.346 | 0.931 | 0.514 | 0.809 | 121 |
| RAD54B(N) | SKP2(C) | 0.267 | 0.834 | 0.519 | 0.300 | 120 |
| BRCA2(N) | RAD54B(N) | 0.785 | 0.267 | 0.519 | 0.266 | 121 |
| RAD54B(N) | WNT5A(C) | 0.267 | 0.471 | 0.522 | 0.007 | 123 |
| EGFR(C) | RAD54B(N) | 0.268 | 0.267 | 0.524 | 0.408 | 120 |
| FEN1(C) | WNT5A(C) | 0.558 | 0.471 | 0.533 | 0.799 | 125 |
| FEN1(N) | PARP1(N) | 0.538 | 0.381 | 0.537 | 0.869 | 123 |
| CSNK1E(C) | FEN1(N) | 0.759 | 0.538 | 0.537 | 0.597 | 119 |
| FEN1(N) | RAD54B(N) | 0.538 | 0.267 | 0.539 | 0.000 | 126 |
| BRCA1(C) | BRCA2(C) | 0.217 | 0.374 | 0.547 | 0.343 | 119 |
| BRCA2(N) | RB1(N) | 0.785 | 0.107 | 0.552 | 0.682 | 118 |
| BRCA1(C) | MSH2(C) | 0.217 | 0.395 | 0.557 | 0.422 | 123 |
| CSNK1E(C) | FEN1(C) | 0.759 | 0.558 | 0.561 | 0.225 | 119 |
| CDH1(M) | RAD54B(N) | 0.346 | 0.267 | 0.564 | 0.002 | 121 |
| ARK2(C) | FEN1(C) | 0.818 | 0.558 | 0.567 | 0.203 | 123 |
| PARP1(N) | WNT5A(C) | 0.381 | 0.471 | 0.573 | 0.720 | 122 |
| CDH1(M) | FEN1(N) | 0.346 | 0.538 | 0.581 | 0.184 | 123 |
| ABL1(C) | FEN1(C) | 0.813 | 0.558 | 0.582 | 0.687 | 123 |
| CSNK1E(C) | SGK2(C) | 0.759 | 0.476 | 0.583 | 0.672 | 119 |
| CDH1(M) | WNT5A(C) | 0.346 | 0.471 | 0.587 | 0.627 | 123 |
| BCR(C) | BRCA1(C) | 0.847 | 0.217 | 0.592 | 0.957 | 121 |
| ABL1(C) | BRCA1(C) | 0.813 | 0.217 | 0.595 | 0.149 | 120 |
| ARK2(C) | CDH1(M) | 0.818 | 0.346 | 0.599 | 0.697 | 122 |
| FEN1(N) | SGK2(C) | 0.538 | 0.476 | 0.608 | 0.558 | 121 |
| FEN1(N) | TP53(N) | 0.538 | 0.144 | 0.625 | 0.638 | 126 |
| ARK2(C) | WNT5A(C) | 0.818 | 0.471 | 0.628 | 0.301 | 123 |
| CDH1(M) | EGFR(C) | 0.346 | 0.268 | 0.637 | 0.443 | 120 |
| BRCA2(N) | MSH2(C) | 0.785 | 0.395 | 0.642 | 0.893 | 122 |
| ABL1(C) | SGK2(C) | 0.813 | 0.476 | 0.642 | 0.191 | 121 |
| MSH2(N) | TP53(N) | 0.923 | 0.144 | 0.648 | 0.608 | 126 |
| SKP2(C) | WNT5A(C) | 0.834 | 0.471 | 0.649 | 0.722 | 122 |
| FEN1(C) | SKP2(C) | 0.558 | 0.834 | 0.649 | 0.867 | 122 |
| ABL1(C) | CDH1(M) | 0.813 | 0.346 | 0.659 | 0.081 | 122 |
| ABL1(C) | WNT5A(C) | 0.813 | 0.471 | 0.659 | 0.630 | 122 |
| BRCA2(C) | EGFR(C) | 0.374 | 0.268 | 0.661 | 0.619 | 117 |
| BCR(C) | RAD54B(N) | 0.847 | 0.267 | 0.664 | 0.047 | 122 |
| CDH1(M) | MSH2(N) | 0.346 | 0.923 | 0.668 | 0.057 | 123 |
| ABL1(C) | RB1(N) | 0.813 | 0.107 | 0.669 | 0.970 | 122 |
| EGFR(C) | MYC(N) | 0.268 | 0.931 | 0.669 | 0.315 | 119 |
| BRCA1(C) | SKP2(C) | 0.217 | 0.834 | 0.671 | 0.890 | 118 |
| ARK2(C) | SGK2(C) | 0.818 | 0.476 | 0.672 | 0.239 | 121 |
| ARK2(C) | POLB(N) | 0.818 | 0.230 | 0.673 | 0.323 | 123 |
| MYC(N) | RB1(N) | 0.931 | 0.107 | 0.682 | 0.744 | 121 |
| EGFR(C) | MSH2(N) | 0.268 | 0.923 | 0.703 | 0.349 | 122 |
| FEN1(N) | SKP2(C) | 0.538 | 0.834 | 0.703 | 0.868 | 122 |
| FEN1(N) | MSH2(N) | 0.538 | 0.923 | 0.708 | 0.309 | 129 |
| BCR(C) | WNT5A(C) | 0.847 | 0.471 | 0.716 | 0.865 | 123 |
| ABL1(C) | CSNK1E(C) | 0.813 | 0.759 | 0.723 | 0.270 | 119 |
| BRCA2(N) | SGK2(C) | 0.785 | 0.476 | 0.730 | 0.835 | 117 |
| PARP1(N) | SKP2(C) | 0.381 | 0.834 | 0.736 | 0.470 | 120 |
| BRCA2(N) | PARP1(N) | 0.785 | 0.381 | 0.742 | 0.916 | 120 |
| MYC(N) | TP53(N) | 0.931 | 0.144 | 0.752 | 0.636 | 121 |
| ARK2(C) | PARP1(N) | 0.818 | 0.381 | 0.756 | 0.246 | 121 |
| SKP2(C) | TP53(N) | 0.834 | 0.144 | 0.760 | 0.551 | 122 |
| MSH2(N) | RB1(N) | 0.923 | 0.107 | 0.762 | 0.170 | 122 |
| BRCA1(C) | BRCA2(N) | 0.217 | 0.785 | 0.762 | 0.398 | 119 |
| BCR(C) | RB1(N) | 0.847 | 0.107 | 0.772 | 0.939 | 121 |
| ARK2(C) | FEN1(N) | 0.818 | 0.538 | 0.790 | 0.809 | 123 |
| FEN1(N) | WNT5A(C) | 0.538 | 0.471 | 0.793 | 0.986 | 125 |
| MSH2(C) | MYC(N) | 0.395 | 0.931 | 0.794 | 0.590 | 121 |
| BRCA2(N) | CSNK1E(C) | 0.785 | 0.759 | 0.794 | 0.780 | 116 |
| ABL1(C) | ARK2(C) | 0.813 | 0.818 | 0.801 | 0.313 | 122 |
| ABL1(C) | POLB(N) | 0.813 | 0.230 | 0.801 | 0.311 | 122 |
| BRCA2(C) | MYC(N) | 0.374 | 0.931 | 0.802 | 0.511 | 118 |
| BRCA2(C) | MSH2(N) | 0.374 | 0.923 | 0.809 | 0.114 | 122 |
| BRCA2(N) | POLB(N) | 0.785 | 0.230 | 0.810 | 0.975 | 120 |
| BRCA1(C) | MYC(N) | 0.217 | 0.931 | 0.816 | 0.402 | 119 |
| MSH2(N) | SGK2(C) | 0.923 | 0.476 | 0.819 | 0.104 | 121 |
| CSNK1E(C) | MSH2(N) | 0.759 | 0.923 | 0.820 | 0.038 | 119 |
| BCR(C) | PARP1(N) | 0.847 | 0.381 | 0.820 | 0.763 | 122 |
| ARK2(C) | CSNK1E(C) | 0.818 | 0.759 | 0.825 | 0.908 | 119 |
| MSH2(C) | WNT5A(C) | 0.395 | 0.471 | 0.826 | 0.317 | 125 |
| ARK2(C) | MSH2(N) | 0.818 | 0.923 | 0.828 | 0.379 | 123 |
| CDH1(M) | FEN1(C) | 0.346 | 0.558 | 0.832 | 0.319 | 123 |
| RB1(N) | SKP2(C) | 0.107 | 0.834 | 0.833 | 0.520 | 121 |
| BCR(C) | POLB(N) | 0.847 | 0.230 | 0.834 | 0.717 | 123 |
| POLB(N) | SKP2(C) | 0.230 | 0.834 | 0.834 | 0.746 | 122 |
| BCR(C) | CSNK1E(C) | 0.847 | 0.759 | 0.841 | 0.682 | 119 |
| ARK2(C) | SKP2(C) | 0.818 | 0.834 | 0.842 | 0.717 | 121 |
| MSH2(N) | MYC(N) | 0.923 | 0.931 | 0.842 | 0.057 | 121 |
| MSH2(N) | PARP1(N) | 0.923 | 0.381 | 0.845 | 0.693 | 123 |
| ABL1(C) | PARP1(N) | 0.813 | 0.381 | 0.846 | 0.439 | 121 |
| BCR(C) | FEN1(C) | 0.847 | 0.558 | 0.853 | 0.666 | 124 |
| BCR(C) | SGK2(C) | 0.847 | 0.476 | 0.853 | 0.811 | 120 |
| ABL1(C) | BRCA2(C) | 0.813 | 0.374 | 0.857 | 0.282 | 119 |
| SGK2(C) | SKP2(C) | 0.476 | 0.834 | 0.858 | 0.480 | 121 |
| FEN1(C) | MYC(N) | 0.558 | 0.931 | 0.864 | 0.550 | 121 |
| MYC(N) | POLB(N) | 0.931 | 0.230 | 0.865 | 0.375 | 121 |
| ABL1(C) | RAD54B(N) | 0.813 | 0.267 | 0.870 | 0.009 | 121 |
| BRCA2(N) | FEN1(C) | 0.785 | 0.558 | 0.874 | 0.935 | 122 |
| BCR(C) | FEN1(N) | 0.847 | 0.538 | 0.875 | 0.057 | 124 |
| MYC(N) | PARP1(N) | 0.931 | 0.381 | 0.877 | 0.315 | 119 |
| FEN1(C) | MSH2(N) | 0.558 | 0.923 | 0.878 | 0.048 | 129 |
| BCR(C) | SKP2(C) | 0.847 | 0.834 | 0.879 | 0.161 | 121 |
| BRCA2(N) | CDH1(M) | 0.785 | 0.346 | 0.880 | 0.302 | 119 |
| BRCA2(N) | SKP2(C) | 0.785 | 0.834 | 0.884 | 0.275 | 118 |
| ARK2(C) | BRCA2(N) | 0.818 | 0.785 | 0.887 | 0.748 | 119 |
| ABL1(C) | EGFR(C) | 0.813 | 0.268 | 0.887 | 0.625 | 121 |
| MYC(N) | SGK2(C) | 0.931 | 0.476 | 0.890 | 0.369 | 120 |
| ABL1(C) | BCR(C) | 0.813 | 0.847 | 0.891 | 0.383 | 122 |
| ARK2(C) | BCR(C) | 0.818 | 0.847 | 0.892 | 0.935 | 122 |
| MSH2(N) | POLB(N) | 0.923 | 0.230 | 0.892 | 0.329 | 124 |
| ABL1(C) | MSH2(C) | 0.813 | 0.395 | 0.893 | 0.223 | 123 |
| ABL1(C) | MSH2(N) | 0.813 | 0.923 | 0.893 | 0.149 | 123 |
| ABL1(C) | BRCA2(N) | 0.813 | 0.785 | 0.893 | 0.275 | 119 |
| MYC(N) | RAD54B(N) | 0.931 | 0.267 | 0.900 | 0.378 | 119 |
| MSH2(N) | WNT5A(C) | 0.923 | 0.471 | 0.900 | 0.178 | 125 |
| ARK2(C) | MYC(N) | 0.818 | 0.931 | 0.902 | 0.289 | 121 |
| BCR(C) | BRCA2(N) | 0.847 | 0.785 | 0.908 | 0.721 | 121 |
| CSNK1E(C) | MYC(N) | 0.759 | 0.931 | 0.911 | 0.161 | 119 |
| CSNK1E(C) | SKP2(C) | 0.759 | 0.834 | 0.913 | 0.852 | 119 |
| BRCA2(N) | MSH2(N) | 0.785 | 0.923 | 0.926 | 0.547 | 122 |
| BCR(C) | BRCA2(C) | 0.847 | 0.374 | 0.927 | 0.760 | 121 |
| ABL1(C) | FEN1(N) | 0.813 | 0.538 | 0.936 | 0.923 | 123 |
| BRCA1(C) | MSH2(N) | 0.217 | 0.923 | 0.936 | 0.080 | 123 |
| ABL1(C) | MYC(N) | 0.813 | 0.931 | 0.936 | 0.678 | 121 |
| CDH1(M) | SKP2(C) | 0.346 | 0.834 | 0.948 | 0.801 | 122 |
| BRCA2(C) | SKP2(C) | 0.374 | 0.834 | 0.948 | 0.976 | 118 |
| MYC(N) | SKP2(C) | 0.931 | 0.834 | 0.948 | 0.544 | 120 |
| MYC(N) | WNT5A(C) | 0.931 | 0.471 | 0.956 | 0.889 | 121 |
| BCR(C) | CDH1(M) | 0.847 | 0.346 | 0.958 | 0.943 | 122 |
| BCR(C) | MYC(N) | 0.847 | 0.931 | 0.973 | 0.704 | 121 |
| BRCA2(N) | MYC(N) | 0.785 | 0.931 | 0.976 | 0.408 | 118 |
| ABL1(C) | SKP2(C) | 0.813 | 0.834 | 0.988 | 0.085 | 121 |
| FEN1(N) | MYC(N) | 0.538 | 0.931 | 0.993 | 0.067 | 121 |
| ABL1(C) | CTNNB1(N) | 0.813 | 1.000 | 1.000 | 0.777 | 122 |
| ARK2(C) | CTNNB1(N) | 0.818 | 1.000 | 1.000 | 0.559 | 122 |
| BCR(C) | CTNNB1(N) | 0.847 | 1.000 | 1.000 | 0.214 | 124 |
| BRCA1(C) | CTNNB1(N) | 0.217 | 1.000 | 1.000 | 0.488 | 121 |
| BRCA2(C) | CTNNB1(N) | 0.374 | 1.000 | 1.000 | 0.516 | 121 |
| BRCA2(N) | CTNNB1(N) | 0.785 | 1.000 | 1.000 | 0.061 | 121 |
| CSNK1E(C) | CTNNB1(N) | 0.759 | 1.000 | 1.000 | 0.404 | 119 |
| CTNNB1(N) | MYC(N) | 1.000 | 0.931 | 1.000 | 0.438 | 121 |
| CDH1(M) | CTNNB1(N) | 0.346 | 1.000 | 1.000 | 0.334 | 122 |
| CTNNB1(N) | EGFR(C) | 1.000 | 0.268 | 1.000 | 0.211 | 120 |
| CTNNB1(N) | MSH2(C) | 1.000 | 0.395 | 1.000 | 0.111 | 125 |
| CTNNB1(N) | MSH2(N) | 1.000 | 0.923 | 1.000 | 0.218 | 125 |
| CTNNB1(N) | TP53(N) | 1.000 | 0.144 | 1.000 | 0.481 | 124 |
| CTNNB1(N) | PARP1(N) | 1.000 | 0.381 | 1.000 | 0.562 | 122 |
| CTNNB1(N) | POLB(N) | 1.000 | 0.230 | 1.000 | 0.545 | 123 |
| CTNNB1(N) | RAD54B(N) | 1.000 | 0.267 | 1.000 | 0.201 | 122 |
| CTNNB1(N) | RB1(N) | 1.000 | 0.107 | 1.000 | 0.569 | 121 |
| CTNNB1(N) | SGK2(C) | 1.000 | 0.476 | 1.000 | 0.389 | 120 |
| CTNNB1(N) | SKP2(C) | 1.000 | 0.834 | 1.000 | 0.198 | 121 |
| CTNNB1(N) | WNT5A(C) | 1.000 | 0.471 | 1.000 | 0.247 | 123 |
| CTNNB1(N) | FEN1(C) | 1.000 | 0.558 | 1.000 | 0.858 | 125 |
| CTNNB1(N) | FEN1(N) | 1.000 | 0.538 | 1.000 | 0.456 | 125 |
| BCR(C) | MSH2(C) | 0.847 | 0.395 | 1.000 | 0.137 | 124 |
| BCR(C) | MSH2(N) | 0.847 | 0.923 | 1.000 | 0.414 | 124 |
| BRCA2(N) | EGFR(C) | 0.785 | 0.268 | 1.000 | 0.289 | 117 |
| MSH2(N) | SKP2(C) | 0.923 | 0.834 | NA | 0.746 | 122 |

**Table S3. The sorted p-values of log-rank-test for the predicted synthetic lethal pairs of lung adenocarcinoma**

| Protein1 | Protein2 | Log Rank | Total Number |
| --- | --- | --- | --- |
|
| Overall Survival (<3 years versus >=3 years) | | | |
| BRCA1(C) | RAD54B(N) | 0.0001 | 112 |
| FEN1(N) | RAD54B(N) | 0.0002 | 117 |
| PARP1(N) | RAD54B(N) | 0.0004 | 113 |
| BRCA1(C) | FEN1(N) | 0.056 | 113 |
| BRCA1(C) | TP53(N) | 0.097 | 113 |
| PARP1(N) | RB1(N) | 0.140 | 111 |
| RB1(N) | TP53(N) | 0.173 | 113 |
| POLB(N) | TP53(N) | 0.204 | 115 |
| FEN1(N) | MSH2(C) | 0.347 | 120 |
| BRCA1(C) | BRCA2(N) | 0.398 | 110 |
| CDH1(M) | RB1(N) | 0.479 | 113 |
| BRCA2(N) | TP53(N) | 0.480 | 112 |
| CDH1(M) | MSH2(C) | 0.525 | 114 |
| MSH2(C) | RB1(N) | 0.581 | 113 |
| EGFR(C) | RB1(N) | 0.588 | 111 |
| MSH2(C) | MYC(N) | 0.590 | 112 |
| PARP1(N) | TP53(N) | 0.617 | 114 |
| CSNK1E(C) | TP53(N) | 0.628 | 110 |
| ARK2(C) | BRCA2(N) | 0.748 | 110 |
| BRCA1(C) | RB1(N) | 0.767 | 110 |
| ABL1(C) | CTNNB1(N) | 0.777 | 113 |
| BRCA2(C) | MSH2(C) | 0.860 | 113 |
| BRCA2(N) | POLB(N) | 0.975 | 111 |
| BRCA1(C) | EGFR(C) | 0.976 | 110 |

**Table S4. Overall survival of 131 lung adenocarcinoma patients in relation to immunohistochemistry of the predicted synthetic lethal pairs analyzed by Univariate Cox regression; three IHC pairs predicted clinical outcome of the patients significantly (P < 0.05).**

| **No.** | **Protein 1** | **Protein 2** | **Subset** | **Hazard ratio (95% C.I.)** | ***p*-value** |
| --- | --- | --- | --- | --- | --- |
| 1 | BRCA1(C) | RAD54B(N) | (－,－)/otherwise | 2.81 ( 1.66 - 4.75 ) | 0.000 |
| 2 | FEN1(N) | RAD54B(N) | (－,－)/otherwise | 2.61 ( 1.53 - 4.43 ) | 0.000 |
| 3 | PARP1(N) | RAD54B(N) | (－,－)/otherwise | 2.44 ( 1.47 - 4.06 ) | 0.001 |
| 4 | BRCA1(C) | FEN1(N) | (－,－)/otherwise | 1.62 ( 0.98 - 2.69 ) | 0.059 |
| 5 | BRCA1(C) | TP53(N) | (－,－)/otherwise | 1.52 ( 0.92 - 2.52 ) | 0.100 |
| 6 | PARP1(N) | RB1(N) | (－,－)/otherwise | 1.46 ( 0.88 - 2.42 ) | 0.143 |
| 7 | RB1(N) | TP53(N) | (－,－)/otherwise | 1.45 ( 0.85 - 2.49 ) | 0.176 |
| 8 | POLB(N) | TP53(N) | (－,－)/otherwise | 1.37 ( 0.84 - 2.24 ) | 0.206 |
| 9 | FEN1(N) | MSH2(C) | (－,－)/otherwise | 1.42 ( 0.68 - 2.99 ) | 0.349 |
| 10 | BRCA1(C) | BRCA2(N) | (－,－)/otherwise | 0.73 ( 0.36 - 1.51 ) | 0.400 |
| 11 | CDH1(M) | RB1(N) | (－,－)/otherwise | 1.2 ( 0.72 - 2.01 ) | 0.479 |
| 12 | BRCA2(N) | TP53(N) | (－,－)/otherwise | 0.66 ( 0.21 - 2.11 ) | 0.483 |
| 13 | CDH1(M) | MSH2(C) | (－,－)/otherwise | 0.78 ( 0.35 - 1.7 ) | 0.526 |
| 14 | MSH2(C) | RB1(N) | (－,－)/otherwise | 0.83 ( 0.42 - 1.63 ) | 0.581 |
| 15 | EGFR(C) | RB1(N) | (－,－)/otherwise | 1.24 ( 0.56 - 2.74 ) | 0.589 |
| 16 | MSH2(C) | MYC(N) | (－,－)/otherwise | 0.78 ( 0.31 - 1.95 ) | 0.591 |
| 17 | PARP1(N) | TP53(N) | (－,－)/otherwise | 1.13 ( 0.69 - 1.85 ) | 0.617 |
| 18 | CSNK1E(C) | TP53(N) | (－,－)/otherwise | 1.13 ( 0.68 - 1.88 ) | 0.628 |
| 19 | ARK2(C) | BRCA2(N) | (－,－)/otherwise | 1.12 ( 0.55 - 2.28 ) | 0.748 |
| 20 | BRCA1(C) | RB1(N) | (－,－)/otherwise | 1.08 ( 0.65 - 1.8 ) | 0.767 |
| 21 | ABL1(C) | CTNNB1(N) | (－,－)/otherwise | 1.23 ( 0.3 - 5.02 ) | 0.777 |
| 22 | BRCA2(C) | MSH2(C) | (－,－)/otherwise | 0.95 ( 0.54 - 1.68 ) | 0.86 |
| 23 | BRCA2(N) | POLB(N) | (－,－)/otherwise | 1.01 ( 0.51 - 2.02 ) | 0.975 |
| 24 | BRCA1(C) | EGFR(C) | (－,－)/otherwise | 0.99 ( 0.49 - 2.02 ) | 0.976 |

**Table S5** (A) The variance inflation factors between any two of the four markers

|  | RAD54B | BRCA1-RAD54B | FEN1(N)-RAD5B | PARP1-RAD54B |
| --- | --- | --- | --- | --- |
| RAD54B |  | 3.50 | 2.43 | 14.72 |
| BRCA1-RAD54B |  |  | 2.02 | 2.75 |
| FEN1(N)-RAD54B |  |  |  | 2.30 |
| PARP1-RAD54B |  |  |  |  |

**Table S5** (B) Adjusted hazard ratios (HRs) of the four markers by three clinical covariates, in the three external datasets.

| **Dataset**  **Variable** | **GSE13213** | | **HLM** | | **TCGA** | |
| --- | --- | --- | --- | --- | --- | --- |
|  | **HR (95% CI*****)** | **p-value** | **HR (95% CI)** | **p-value** | **HR (95% CI)** | **p-value** |
| **BRCA1-RAD54B and three clinical factors** | | | | | | |
| BRCA1-RAD54B | NA^†^ | NA | 3.86 ( 0.46 - 32.36 ) | 0.213 | 0.69 ( 0.20 - 2.35 ) | 0.553 |
| Age | 1.29 ( 0.72 - 2.31 ) | 0.391 | 1.12 ( 0.64 - 1.94 ) | 0.691 | 1.47 ( 0.90 – 2.38 ) | 0.122 |
| Sex | 1.52 ( 0.85 - 2.69 ) | 0.156 | 0.78 ( 0.46 - 1.31 ) | 0.339 | 0.79 ( 0.49 - 1.27 ) | 0.321 |
| Stage | 3.14 ( 1.74 - 5.67 ) | $1.5\times10^{- 4}$ | 2.17 ( 1.18 – 4.00 ) | 0.012 | 2.78 ( 1.73 - 4.48 ) | $2.5\times10^{- 5}$ |
| **RAD54B and three clinical factors** | | | | | | |
| RAD54B | 15.87 ( 3.39 - 74.19 ) | $4.4\times10^{- 4}$ | 3.86 ( 0.46 - 32.36 ) | 0.213 | 1.94 ( 1.09 - 3.45 ) | 0.024 |
| Age | 1.37 ( 0.76 - 2.48 ) | 0.290 | 1.12 ( 0.64 - 1.94 ) | 0.691 | 1.49 ( 0.92 - 2.41 ) | 0.107 |
| Sex | 1.56 ( 0.88 - 2.77 ) | 0.129 | 0.78 ( 0.46 - 1.31 ) | 0.339 | 0.71 ( 0.44 - 1.15 ) | 0.167 |
| Stage | 3.17 ( 1.76 - 5.72 ) | $1.3\times10^{- 4}$ | 2.17 ( 1.18 - 4.00) | 0.012 | 2.43 ( 1.50 - 3.93 ) | $2.9\times10^{- 4}$ |
| **FEN1(N)-RAD54B and three clinical factors** | | | | | | |
| FEN1(N)-RAD54B | NA | NA | NA | NA | 1.74 ( 1.08 - 2.81 ) | 0.024 |
| Age | 1.29 ( 0.72 - 2.31 ) | 0.391 | 1.14 ( 0.66 - 1.97 ) | 0.647 | 1.49 ( 0.92 - 2.43 ) | 0.105 |
| Sex | 1.52 ( 0.85 - 2.69 ) | 0.156 | 0.76 ( 0.45 - 1.28 ) | 0.300 | 0.76 ( 0.47 - 1.22 ) | 0.249 |
| Stage | 3.14 ( 1.74 - 5.67 ) | $1.5\times10^{- 4}$ | 2.28 ( 1.26 - 4.13 ) | 0.007 | 2.61 ( 1.62 - 4.19 ) | $7.4\times10^{- 5}$ |
| **PARP1-RAD54B and three clinical factors** | | | | | | |
| PARP1-RAD54B | NA | NA | NA | NA | 1.83 ( 1.12 – 3.00 ) | 0.016 |
| Age | 1.29 ( 0.72 - 2.31 ) | 0.391 | 1.14 ( 0.66 - 1.97 ) | 0.647 | 1.44 ( 0.89 - 2.33 ) | 0.138 |
| Sex | 1.52 ( 0.85 - 2.69 ) | 0.156 | 0.76 ( 0.45 - 1.28 ) | 0.300 | 0.67 ( 0.41 - 1.10 ) | 0.118 |
| Stage | 3.14 ( 1.74 - 5.67 ) | $1.5\times10^{- 4}$ | 2.28 ( 1.26 - 4.13 ) | 0.007 | 2.58 ( 1.60 - 4.15 ) | $9.4\times10^{- 5}$ |

*CI denotes confidence interval; ^†^ the gene(s) analyzed was (were) not 2-fold over- or under-expressed.
